# Genome-wide expressions in autologous eutopic and ectopic endometrium of fertile women with endometriosis

**DOI:** 10.1186/1477-7827-10-84

**Published:** 2012-09-24

**Authors:** Meraj A Khan, Jayasree Sengupta, Suneeta Mittal, Debabrata Ghosh

**Affiliations:** 1 Department of Physiology, All India Institute of Medical Sciences, New Delhi, India; 2 Department of Obstetrics and Gynecology, All India Institute of Medical Sciences, New Delhi, India

**Keywords:** Computational analysis, Endometriosis, Differential display, Gene expression, GSEA

## Abstract

**Background:**

In order to obtain a lead of the pathophysiology of endometriosis, genome-wide expressional analyses of eutopic and ectopic endometrium have earlier been reported, however, the effects of stages of severity and phases of menstrual cycle on expressional profiles have not been examined. The effect of genetic heterogeneity and fertility history on transcriptional activity was also not considered. In the present study, a genome-wide expression analysis of autologous, paired eutopic and ectopic endometrial samples obtained from fertile women (n = 18) suffering from moderate (stage 3; n = 8) or severe (stage 4; n = 10) ovarian endometriosis during proliferative (n = 13) and secretory (n = 5) phases of menstrual cycle was performed.

**Methods:**

Individual pure RNA samples were subjected to Agilent’s Whole Human Genome 44K microarray experiments. Microarray data were validated (P < 0.01) by estimating transcript copy numbers by performing real time RT-PCR of seven (7) arbitrarily selected genes in all samples. The data obtained were subjected to differential expression (DE) and differential co-expression (DC) analyses followed by networks and enrichment analysis, and gene set enrichment analysis (GSEA). The reproducibility of prediction based on GSEA implementation of DC results was assessed by examining the relative expressions of twenty eight (28) selected genes in RNA samples obtained from fresh pool of eutopic and ectopic samples from confirmed ovarian endometriosis patients with stages 3 and 4 (n = 4/each) during proliferative and secretory (n = 4/each) phases.

**Results:**

Higher clustering effect of pairing (cluster distance, cd = 0.1) in samples from same individuals on expressional arrays among eutopic and ectopic samples was observed as compared to that of clinical stages of severity (cd = 0.5) and phases of menstrual cycle (cd = 0.6). *Post hoc* analysis revealed anomaly in the expressional profiles of several genes associated with immunological, neuracrine and endocrine functions and gynecological cancers however with no overt oncogenic potential in endometriotic tissue. Dys-regulation of three (CLOCK, ESR1, and MYC) major transcription factors appeared to be significant causative factors in the pathogenesis of ovarian endometriosis. A novel cohort of twenty-eight (28) genes representing potential marker for ovarian endometriosis in fertile women was discovered.

**Conclusions:**

Dysfunctional expression of immuno-neuro-endocrine behaviour in endometrium appeared critical to endometriosis. Although no overt oncogenic potential was evident, several genes associated with gynecological cancers were observed to be high in the expressional profiles in endometriotic tissue.

## Background

**Figure 1:**
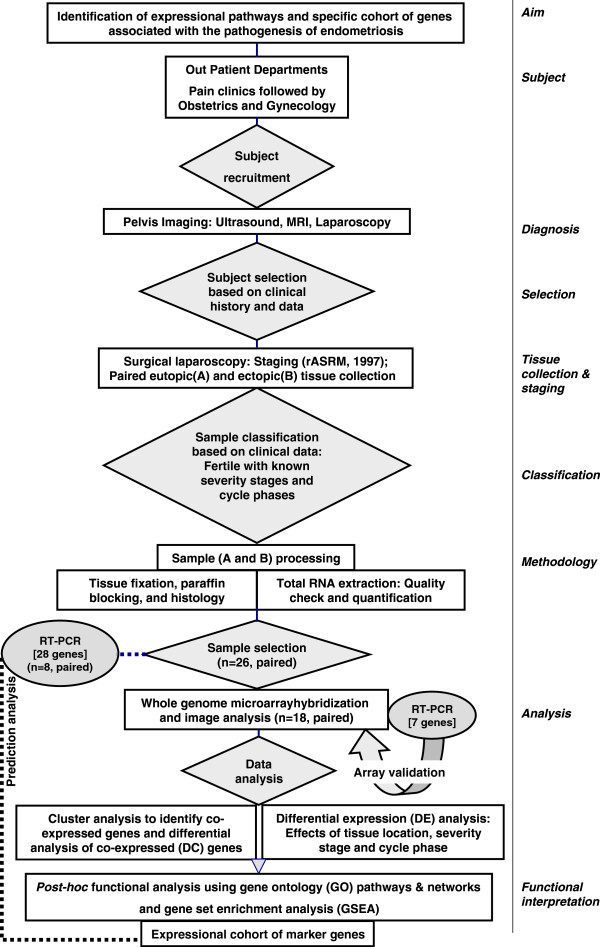
Flow diagram of the experimental design showing overall aim and work plan of the present study.

Endometriosis is a complex disorder involving pathogenesis and clinical presentation of ectopically implanted endometrium [1]. It is generally assumed that elucidation of molecular expressional specificities of eutopic and ectopic endometrium may provide leads towards a better understanding of the pathophysiology of the disorder [2]. To this end, several studies exploring the differential expression of genes between autologous eutopic and ectopic endometrium from patients with endometriosis have been reported, however, with no specific comparison for stages of severity, fertility history and phases of menstrual cycle [3-7], except a recent report [8]. More over, it is notable that two types of endometriosis, namely ovarian endometriosis and peritoneal endometriosis reportedly show differential characteristics [4,9]. Furthermore, there is evidence to support the idea that deep infiltrating endometriosis also show differential pathophysiology as compared to ovarian and peritoneal endometriosis [10,11]. In the present study, we examined a genome-wide large-scale transcript survey of autologous, paired eutopic and ectopic endometrial samples obtained from fertile women suffering from moderate to severe ovarian endometriosis, and excluded cases of peritoneal endometriosis and deep infiltrating endometriosis. We assumed that the present model of subject selection would reduce the impact of biological noise derived from genetic and pathogenetic heterogeneity and subfertility-associated variability on the transcriptional activity in the target tissue. We report here for the first time that clustering effect of expressional arrays among eutopic and ectopic samples was higher for genetic homogeneity (i.e. pairing of eutopic and ectopic samples from same individuals) than that of clinical stages of severity and phases of menstrual cycle. Based on the present transcriptomics data, we have also hypothesized that dysfunctional immuno-neuro-endocrine behaviour in endometrium was associated with the pathogenesis of endometriosis. Additionally, we did not observe an overt oncogenic potential in the expressional profiles in endometriotic tissue, however, several genes associated with gynecological cancers were highly expressed in the eutopic and ectopic endometrium. Finally, a novel cohort of 28 genes was identified, the expression of which carry potential marker value for endometriosis in fertile women. A flow diagram of the experimental design is shown in Figure 1.

## Methods

### Subjects and tissue samples

**Figure 2:**
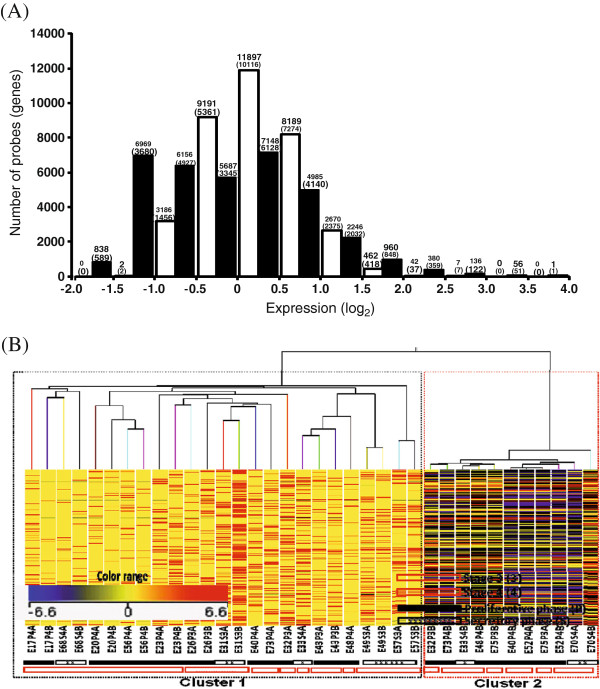
**General descriptive characteristics of expressions in eutopic and ectopic endometrium.** (**A**) Histogram of frequency distribution of probes (genes) for different groups of expression levels (in log_2_) in eighteen (18) autologous, paired eutopic (*blank bar*) and ectopic (*hatched bar*) endometrial samples. (**B**) Two-way representation of unsupervised hierarchical cluster analysis (HCA) of the expression levels (in logarithmic scale) of all the target probes/genes (Y-axis) in each sample (each column), eutopic labelled as *A* and ectopic labelled as *B* from all subjects (n = 18) and their clustering based on expressional distance (Pearson correlation coefficient) between samples in dendrogram formation (X-axis). Each horizontal line represents a single probe, and each column represents a single sample. Relative expression of each probe is colour-coded: high (*red*) and low (*blue*), as indicated in the colour legend. Categorical annotations of each sample are shown in the X-axis. The samples cluster by cycle phase and severity stages, as shown by the bar at the bottom of the heat map: proliferative phase (*black bar*), secretory phase (*crossed white bar*), stage 3 (*red crossed bar*) and stage 4 (*blank red bar*). A, eutopic; B, ectopic; P, proliferative phase; S, secretory phase; 3, stage 3; 4, stage 4.

The present study was approved by the Ethics Committee on the Use of Human Subjects, All India Institute of Medical Sciences (AIIMS), New Delhi. The patients enrolled in the Department of Obstetrics and Gynecology – AIIMS and showing evidence of endometriotic lesions, adhesions and endometriotic cyst were selected to participate in the present study. All the patients were reportedly fertile and referred from the Pain Clinics, and had voluntarily agreed to donate their samples after understanding the purpose of the proposed study. Signed informed consent was obtained from each participant of this study. As shown in Figure 1, twenty-six (26) normally cycling and proven fertile women (age: 24–45 y) with history of pregnancy and with at least one living biological offspring, and body mass indices within normal ranges (20–22 k/m^2^) having ovarian endometriosis were selected for the present study. Confirmation of ovarian endometriosis and exclusion of other types of endometriosis was achieved from reports of pelvic imaging based on ultrasound, MRI and/or diagnostic laparoscopy as described elsewhere [8]. Severity stages 3 and 4 of the disease condition were defined at the time of surgical laparoscopy [8] according to rASRM protocol [12]. Selected subjects (n = 18; shown as ‘E’ in Additional file 1: Table S1) contributed their eutopic (shown as ‘A’ in Figure 2) and ectopic (shown as ‘B’ in Figure 2) samples during proliferative (days 9–14) phase (n = 17) and secretory (days 17–24) phase (n = 8) of menstrual cycle as described elsewhere [8]. Additional paired samples collected from different group of subjects (n = 8; shown as ‘Ep’ in Additional file 1: Table S1) with confirmed ovarian endometriosis as described above and with classified menstrual (proliferative: n = 4; secretory: n = 4) phases and severity stages 3 (n = 4) and 4 (n = 4) were employed for validating the prediction as described below. A small piece from each specimen was processed for chemical fixation in neutral buffered formaldehyde (4%, w/v) for subsequent confirmation of phase of cycle, state of pathology and cell types from eutopic and ectopic samples, and the residual portions were transported on ice to the laboratory within 10 minutes of collection for further processing for RNA extraction.

### Experimental procedure

The methodological details of RNA extraction followed by the estimation of its yield and purity using standard electrophoretic and spectrophometric protocols and its RIN score using the Agilent 2100 Bioanalyzer, RNA 6000 Nano LabChip kit and Agilent 2100 Expert Software (Agilent Technologies, Santa Clara, CA, USA) have been given elsewhere [8,13]. Individual RNA samples from eutopic and ectopic tissue samples (n = 18) from confirmed stages 3 (n = 8) and 4 (n = 10) collected during proliferative (n = 13) and secretory (n = 5) phases and having RIN scores >8.0 were subjected to whole transcriptome array experiment using the Agilent Whole Human Genome 60-mer 4X44K microarray according to the manufacturer’s recommendations. Thus, seven (7) samples could not be used either for insufficient RNA yield or RIN scores (see Additional file 1: Table S1 for the subject details of the selected samples). Hybridized arrays were scanned with Agilent’s G2505B microarray scanner system and the raw data were imported into GeneSpring 11.5.0 software (Agilent Technologies, Santa Clara, CA, USA) for further analysis. Pearson’s correlation coefficients done to assess the reliability of data obtained from two separate hybridization runs for same RNA preparation for four (4) eutopic and ectopic samples confirmed the reproducibility assurance (P < 0.01) among hybridizations. Analysis of the data retrieved from separate chips with the same RNA samples yielded QC statistics highly concordant with that of the manufacturer, and it revealed more than 95% confidence level.

### Data analysis

Unsupervised and supervised hierarchical clustering analysis (HCA), and non-hierarchical K-mean cluster analysis of expression arrays were performed with the help of GeneSpring software 11.5.0. Analysis of variance followed by pair-wise differential (>3-fold at P < 0.01) expression (DE) for specific genes between eutopic and ectopic samples, as well as, between proliferative and secretory phases, and between clinical stage 3 and stage 4 of severity for eutopic endometrium, and for ectopic endometrium, respectively were done using multiple comparison tests as described elsewhere [14].

### Post-hoc analysis

Networks and enrichment analysis were done using gene lists obtained from the above analyses and based on *a priori* setting of a cut-off threshold (pFDR(p) = 0.05) with the help of the GeneSpring11.5.0 software and Metacore platform (GeneGo, St. Joseph, MI, USA). The K-mean clusters were further used for differential co-expression (DC) analyses and analyzed in terms of Gene Ontology (GO) enriched categories using GeneSpring11.5.0 software. Gene Set Enrichment Analysis (GSEA) version 3.7 was applied to each of the K-mean clusters independently to examine at FDR ≤ 0.25 for not less than 10 genes for a set with a maximum of 1000 permutation whether pre-annotated BROAD gene sets [15]: C1 (cytogenetic sets), C2 (functional sets), C3 (regulatory sets), C4 (cancer neighborhood sets), and C5 (gene ontology sets) could identify any interesting information in the DC sets [16].

### Quantification of candidate gene expression by real time RT-PCR

In order to validate the microarray data, relative expression of arbitrarily chosen seven (7) selected genes (*ATX*, *DDHD1*, *DYNLT1*, *FTH1*, *LAMR1*, *MIER2*, and *WDR87*) in eutopic and ectopic samples collected from all patients were performed using Taqman multiplexing technology on iCycler iQTm real time RT-PCR detection system (BioRad, Hercules, CA, USA). GAPDH was selected as an endogenous control based on its observed expressional consistency in arrays on data analysis. Primers and probes were designed on Beacon Designer software7 (Labware Scientific Inc., Milipitas, CA, USA) and obtained from Qiagen (Cologne, Germany) (see Additional file 2: Table S2 for the details). The ratio of estimated efficiency of the primers for the selected genes and GAPDH was ~1.0. An optimized kit (QuantiTect multiplex PCR kit, Qiagen, Cologne, Germany) was used to synthesize cDNA from respective RNA (5 μg) samples. Relative expression ratios between groups were calculated by using 2^-ΔΔCt^ method [17]. Quantification of copy numbers for target transcripts in complex RNA samples was obtained as described elsewhere [18]. Comparison between fold change data obtained from real time RT-PCR and microarray image analysis for selected seven (7) genes revealed a high degree of concordance and pattern similarity in expression profile. Concordance correlation test between real time RT-PCR based quantitative data and microarray data for the seven (7) genes showed a high degree of correlation (P < 0.01) [8].

In order to test the reproducibility of prediction derived from analysis of microarray results, the relative expressions of twenty eight (28) selected genes in individual RNA samples obtained from eight (8) additional subjects giving paired eutopic and ectopic samples with confirmed endometriosis stages 3 (n = 4) and 4 (n = 4) during proliferative (n = 4) and secretory (n = 4) were examined using real time RT-PCR technology. The subject details are shown in Additional file 1: Table S1. The genes selected based on GSEA implementation of DC results were employed to test the predictability function of the expression of those genes. The details of RNA methodologies are given above. GAPDH was selected as an endogenous control based on its observed expressional consistency in arrays on data analysis. All primers were designed on the Beacon Designer software7.0 (Labware Scientific Inc., Milipitas, CA, USA) based on SYBR green chemistry and obtained from Qiagen (Cologne, Germany). QuantiTect Reverse Transcription kit for cDNA synthesis and QuantiFast SYBR green PCR kit for PCR amplification from Qiagen (Cologne, Germany) were used according to the protocol given by the manufacturer. The estimates of relative expression ratios between groups and copy numbers for target transcripts in complex RNA samples were obtained as described above.

## Results

The data sets are available at NCBI-GEO website [19].

A distribution histogram of the number of probes and genes for different ranges of expression in autologous, paired eutopic and ectopic samples obtained from eighteen (18) fertile women with confirmed ovarian endometriosis is shown in Figure 2A. Total numbers and per cent estimates of probes/genes expressed in eutopic and ectopic samples in optimized scale are shown in Table 1. On average, ~75% and ~50% of expressed genes showed marked signal in eutopic and ectopic samples, respectively. Unsupervised HCA yielded marked segregation of samples into two major clustering branches with clustering cohesion being highest (cluster distance, cd: 0.1) between paired samples from same subjects. However, clustering cohesion was only moderate in samples which were classified based on either severity stages (cd: 0.5) or phases of menstrual cycle (cd: 0.6) (Fig. 2B). Supervised HCA revealed that the ectopic location of tissue had a higher clustering effect (cd: 0.2) than that of phases of cycle (cd: 0.3), but not than that of the clinical stages of severity (cd: 0.1).

**Table 1:** Descriptive analysis of array data

**Parameter**	**Estimate**	
	**Per chip**	**Per cent**
**Number of probes**	41000	
(genes)	(29421)	
**Number of hybridized probes (genes)**^**a**^		
Eutopic	35646	87
	(25987)	(88)
Ectopic	35587	87
	(26222)	(89)
**Number of high expressed probes (genes)**^**b**^		
Eutopic	23267	65
	(19168)	(74)
Ectopic	15912	45
	(13681)	(52)

^a^Hybridization signal more than mean optimized background signal ± 2SD.

^b^ > 0 in normalized log_2_ scale.

### Differential expression (DE)

**Figure 3:**
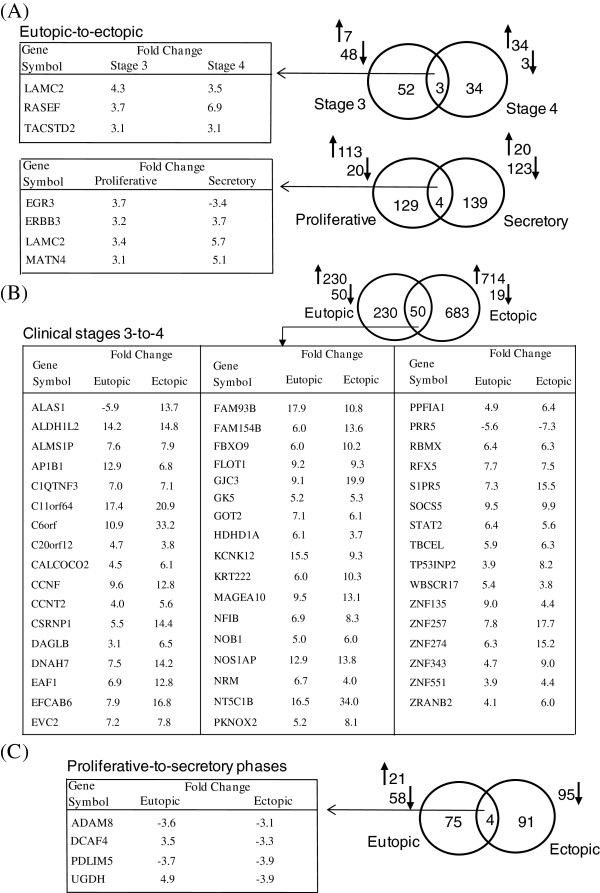
**Venn analysis of distribution of differentially expressed (DE) genes in eutopic-to-ectopic analysis.** Distribution of DE genes in (**A**) eutopic and ectopic samples of stage 3 and stage 4, and proliferative and secretory phases, (**B**) stage 3-to-stage 4 for eutopic and ectopic samples, and (**C**) proliferative-to-secretory phases for eutopic and ectopic samples. Common genes among comparative groups are detailed in respective tables along with the vector of regulation and fold changes. The number of genes with relative up-regulation and down-regulation are shown by respective *arrows*. For details of DE genes, see Additional file 3: Table S3. Note that the areas in the Venn distribution analysis are not drawn to scale.

Additional file 3: Table S3 gives the list of the genes along with their differential expression (DE) patterns under different categories based on expressional arrays in autologous, paired eutopic and ectopic samples obtained from 18 fertile women with ovarian endometriosis. Figure 3 shows the number of genes with DE in different categories of comparison and the lists of common genes in it. Table 2 highlights the enriched categories of pathways for the common genes from above-mentioned DE analysis between eutopic and ectopic endometrium. It appeared that different signaling pathways associated with immune response, several neuronal processes, and ERBB family signaling pathways were commonly selected. A summary of DE analysis of the non-common genes showing differential display under different categories and their enrichment analysis are shown in Table 3. Collectively, it appeared that informational flow for a wide array of pathways involving cellular signaling, apoptosis and survival, cytoskeleton remodeling, chemotaxis, cell adhesion, immune response and several neurophysiological processes were affected.

**Table 2:** **Enriched common genes showing differential changes under different categories of comparisons**^a^

**Description of comparison (Number of genes)**	**Gene in enriched category (Gene symbol)**	**Enriched pathways**	**(p-value)**
**Eutopic-to-ectopic**	ERBB3	Activation of astroglia proliferation	(0)
Stage 3 & Stage 4 (3)^b^		CDK5 mediated cell death and survival	(0)
Proliferative & Secretory (4)^b^		ERBB family signaling	(0)
		Membrane bound ESR1 interaction with growth factor signaling	(<0.01)
		Ligand-independent activation of ESR1 and ESR2	(<0.01)
	ERBB3, LAMC2	Alpha6/beta-4 integrins in carcinoma progression	(<0.01)
**Stages 3-to-4**	STAT2	Immune response involving IL-15 and IFN signaling	(<0.02)
Eutopic & Ectopic (50)^c^			
**Proliferative-to-Secretory**		Angiotensin signaling via STATs	(<0.03)
Eutopic & Ectopic (4)^d^	NOS1AP	nNOS signaling in neuronal process	(<0.03)
	AP1B1	Immune response involving regulation of T cell function by CTLA-4	(<0.04)
	SOCS5	Immune response involving IL-4 signaling	(<0.04)
	GOT2	GABA biosynthesis and metabolism	(<0.05)

^a^see Figure 2, ^b^A, ^c^B and ^d^C.

**Table 3:** **Estimates and enriched categories of differentially**^**a**^**regulated non-common genes**

**Specific analysis**	***Nature of differential change [Number of genes]***	**Top enriched pathways**	**[Gene symbol(s) of major candidate(s)]**	**(p-value)**
**Pooled**	*Up-regulated [50]*	WNT signaling	[NRCAM, WNT16]	(0)
		DNA damage-induced responses and apoptosis	[CHEK1]	(<0.01)
		Role of 14-3-3 proteins in cell cycle regulation	[CHEK1]	(<0.02)
		Cadherins mediated cell adhesion	[CHP]	(<0.03)
		Endothelial cell contacts by non-junctional mechanisms	[CHP]	(<0.03)
		Role of SCF complex in cell cycle regulation	[CHEK1]	(<0.03)
		ATM/ATR regulation of cell cycle	[CHEK1]	(<0.04)
		nNOS signaling in neuronal synapses	[RASD1]	(<0.03)
		Activation of astroglial cell proliferation by ACM3	[ERBB3]	(<0.04)
		G-protein signaling in RhoA regulation pathway	[ARHGAP26]	(<0.04)
		CDK5 in apoptosis and survival	[ERBB3]	(<0.04)
		ERBB-family signaling	[ERBB3]	(<0.05)
		Regulation of ElF2 activity associated with translation	[CSNK1G1]	(<0.05)
		Ligand-independent activation of ESR1 and ESR2	[ERBB3]	(<0.05)
		Non-genomic action of androgen receptor	[WNT16]	(<0.05)
	*Down-regulated [41]*	Regulation of glucose and lipid metabolism	[APOE]	(0)
		GDNF signaling	[ITGB1]	(<0.04)
		Immune response involving antigen presentation by MHC class I	[HLA-C]	(0.05)
		Chemotaxis involving CCR4-induced leukocyte adhesion	[ITGB1]	(<0.05)
**Stage 3**	*Up-regulated [4]*	No specific enriched category identified		
	*Down-regulated [48]*	Cytoskeleton remodeling involving RalB and RalA regulation pathway	[RALGDS]	(<0.01)
		Clathrin coated vesicle formation	[MYO1D]	(<0.02)
		Transcriptional silencing involving HP1 family	[PFDN5]	(<0.02)
		G-protein signaling involving interaction among Ras-family GTPases and K-RAS/N-RAS/H_RAS regulation pathway	[RALGDS]	(<0.03)
**Stage 4**	*Up-regulated [31]*	Cell contraction involving relaxin and GPCRs	[ADCY6, EDNRA, RXFP1]	(0)
		Development involving endothelin-1/EDNRA signaling	[ADCY6, EDNRA]	(0)
		DNA damage induced apoptosis and DNA repair	[NBN]	(<0.01)
		Beta-2 adrenergic dependent CFTR expression	[ADCY6]	(<0.01)
		Regulation of lipid metabolism	[PPARA]	(<0.02)
		Alpha-1 adrenergic receptor signaling	[ADCY6]	(<0.02)
		Mu- and kappa-type opioid receptor mediated physiological process	[ADCY6]	(<0.03)
		Mucin expression via IL-6, IL-17 signaling pathways	[TRAF3IP2]	(<0.04)
		G-protein signaling	[ADCY6]	(<0.04)
	*Down-regulated [3]*	Transport from Golgi and ER to the apical membrane	[PPIA]	(0)
		Intracellular cholesterol and sphingolipids transport	[PPIA]	(<0.01)
**Proliferative phase**	*Up-regulated [109]*	RAS regulation pathway	[BCR, RASGRF1]	(0)
		TC21 regulation pathway	[BCR, RASGRF1]	(0)
		Regulation of CDC42 activity	[BCR, FGFR1]	(<0.01)
		Sin3 and NuRD mediated transcription regulation	[CHD3, SIN3A]	(<0.01)
		GDNF family signaling	[GFRA2, NRTN]	(<0.01)
		Phospholipid metabolism	[GPD2, NRTN]	(<0.02)
		Immune response involving CD40 signaling	[IRF1, TRAF3IP2]	(<0.02)
	*Down-regulated [20]*	Cytoskeleton remodeling involving α-1A adrenergic receptor		
		Dependent inhibition of PI3K and regulation of actin by Rho GTPases	[LAMB1, MYL12B]	(<0.01)
		Cell contraction involving δ-type opioid receptor, S1P2 receptor, ACM	[MYL12B]	(<0.01)
		Development associated MAG dependent inhibition of neurite outgrowth	[MYL12B]	(<0.01)
		Development associated with TGF-beta dependent induction of EMT via RhoA, PI3K and ILK	[TPM1]	(<0.01)
		Cell adhesion involving histamine H1 receptor	[MYL12B]	(<0.01)
		Cell adhesion and chemotaxis involving integrin	[LAMB1, MYL12B]	(<0.01)
		Chemotaxis involving inhibitory action of lipoxins on IL-8 and leukotriene B4-induced neutrophil migration	[MYL12B]	(<0.01)
		GPCRs in platelet aggregation	[MYL12B]	(<0.02)
		Immune response involving CCR3 signaling in eosinophils	[MYL12B]	(<0.02)
		Oxidative phosphorylation	[UQCR11]	(<0.03)
**Secretory phase**	*Up-regulated [17]*	Transport involving RAN regulation pathway	[TNPO1]	(<0.01)
		Immune response involving MIF-JAB1 signaling	[PGR]	(<0.01)
		nNOS signaling in neuronal synapses and circadian rhythm	[RASD1]	(<0.02)
		Cell cycle associated spindle assembly and chromosome separation	[TNPO1]	(<0.02)
		Regulation of lipid metabolism	[TNPO1]	(<0.02)
		Regulation of glycogen metabolism	[AGL]	(<0.02)
		Progesterone mediated maturation	[PGR]	(<0.02)
		Cell adhesion associated ECM remodeling	[MME]	(<0.03)
		TGF-beta receptor signaling in development	[TNPO1]	(<0.03)
	*Down-regulated [122]*	Cell contraction involving δ-type opioid receptor	[MYL9]	(0)
		Development associated Slit-Robo signaling	[DPYSL2, ROBO3]	(<0)
		Insulin mediated regulation of translation	[ElF4EBP1, PPP1CC]	(<0.01)
		Leukotriene 4 biosynthesis and metabolism	[GGT5, LTA4H]	(<0.01)
		Chemotaxis involving inhibitory action of lipoxins on IL-8 and leukotriene B4-induced neutrophil migration	[MYL9, RAC2)	(<0.01)
		Endoplasmic reticulum stress response pathway	[ATF4, PPP1CC]	(<0.01)
		Immune response involving CCR3 signaling in eosinophils	[MYL9, RAC2]	(<0.03)
		GTP-XTP metabolism	[GUK1, NME3, POLR3H]	(<0.03)
		Cytoskeleton remodeling via RalB regulation pathway	[RALGDS]	(<0.04)
**Eutopic**	*Up-regulated [182]*	Cytoskeleton remodeling involving ACM3 and ACM4	[CHRM4, GNAQ]	(0)
		G-protein signaling involving regulation of cAMP levels by ACM	[CHRM4, GNAQ]	(0)
		Transcription involving Tubby signaling and HP1 family	[GNAQ]	(0)
		Regulation of lipid metabolism involving G-alpha(q) regulation	[GNAQ, PTGS2]	(0)
		Cell contacts by non-junctional mechanisms	[ITGA5, MAG1, PECAM1]	(<0.01)
		NMDA –dependent neurophysiological process	[GNAQ, GRIN2A]	(<0.01)
		Cell cycle at metaphase check point	[CBX3, INCENP]	(<0.01)
		G-protein signaling involving Rap1A regulation pathways	[MAGI1, RAPGEF1]	(<0.02)
		Regulation of translation through EIF4F activity	[EIF4A2]	(<0.03)
		Regulation of translation by alpha-1 adrenergic receptors	[EIF4A2, GNAQ]	(<0.03)
		Development involving endothelin-1/EDNRA signaling	[GNAQ, NPPB]	(<0.03)
		Cytoskeleton remodeling via FAK signalin	[GNAQ, RAPGEF1]	(<0.04)
		Immune response involving PGE2 common pathways	[GNAQ, PTGS2]	(<0.03)
		Immune response involving IL-17 signaling pathways	[CXCL3, PTGS2]	(<0.04)
		Cell contraction via oxytocin signaling	[GNAQ, PTGS2]	(<0.04)
		Transcription via PPAR pathway	[MED1, PTGS2]	(<0.04)
		Regulation of lipid metabolism through alpha-1 adrenergic receptors signaling via arachidonic acid	[GNAQ, PTGS2]	(<0.05)
	*Down-regulated [48]*	Cell cycle regulation involving SCF complex	[CDC34, NEDD8, UBA52]	(0)
		p53 regulation involving SUMO	[UBA52]	(<0.02)
		Regulation of degradation and traffic of CFTR	[DYNLL1, UBA52]	(<0.02)
		WNT signaling pathway involving degradation of bete-catenin	[UBA52]	(<0.03)
		Transcriptional silencing involving HP1 family	[PFDN5]	(<0.03)
		Immune response involving IL-12 and MIF-JAB1 signaling pathways	[UBA52]	(<0.03)
		Angiotensin signaling via beta-arrestin	[CLTA, UBA52]	(<0.03)
		ATM/ATR regulation of G1/S and G2/M checkpoints	[UBA52]	(<0.04)
		NGF signaling for apoptosis and survival	[EPB41L1]	
		and activation of NF-kB	[UBA52]	(<0.04)
		Neurophysiological process involving GABA-A receptor life cycle	[CLTA]	(<0.04)
		Regulation of translation initiation	[EIF1, RPL7, RPL12, RPL15, RPL21, RPL22, RPL29, RPS3A, RPS10, RPS14, UBA52]	(<0.04)
		Transition and termination of DNA replication	[UBA52]	(<0.04)
		Activin A signaling regulation	[UBA52]	(<0.05)
**Ectopic**	*Up-regulated [665]*	Beta-2 adrenergic-dependent CFTR expression	[ADCY2, ADRB3, CREB1, PRKAR1B, PRKAR2B]	(0)
		Mu-type opioid receptor mediated neurophysiological process	[ADCY2, ADCY5, CREB1, HPCA, PRKAR1B, PRKAR2B]	(0)
		Development involving alpha-1 and beta-adrenergic receptors signaling via cAMP and PIP3 signaling	[ADCY2, ADCY5, AKT3, CREB1, FOXO3, GAB1, PRKAR1B, PRKAR2B, YWHAE]	(0)
		Cell adhesion involving ephrin signaling	[ADAM10, EFNA5, EPHA4, EPHB6]	(0)
		Transport involving RAB3 regulation pathway	[DMXL2, RAB3B]	(0)
		Neurophysiological process involving corticoliberin signaling via CRHR1	[ADCY2, ADCY5, CACNA1C, CREB1, PRKAR2B, IVL]	(0)
		Signal transduction involving cAMP and PKA signaling	[ADCY2, ADCY5, CACNA1C, CREB1, PRKAR1B, PRKAR2B, PCTK1]	(0)
		G-protein signaling involving G-Protein beta/gamma signaling cascades	[ADCY2, ADCY5, AKT3, PRKAR1B, PRKAR2B]	(0)
		G-protein signaling involving RhoA regulation pathway	[ARHGEF2, EFNA5, EPHA4, MCF2L]	(0)
		eNOS activity in cell contraction	[ADCY5, CACNA1C, PRKAR1B,PRKAR2B, PRKG1]	(0)
		MAG-dependent inhibition of neurite outgrowth	[NGFR, PSEN2, RASGRF1]	(0)
		Neurophysiological process involving delta-type opioid receptor	[ADCY2, CREB1, HPCA, PRKAR1B, PRKAR2B]	(<0.01)
		Neurophysiological process involving HTR1A receptor signaling	[ADCY2, ADCY5, HPCA, HTR1A, PRKAR1B, PRKAR2B]	(<0.01)
		Neurophysiological process involving melatonin signaling	[ADCY2, ADCY5, CREB1, PRKAR1B, PRKAR2B, RORA]	(<0.01)
		Neurophysiological process involving dopamine D2 receptor signaling	[ADCY2, ADCY5, CACNA1C, PRKAR1B, PRKAR2B]	(<0.01)
		Neurophysiological process in circadian rhythm	[ADCAY1, CLOCK, CREB1, CACNA1C, RORA]	(<0.01)
		Alpha-2 adrenergic receptor regulation of ion channels	[ADCY5, AKT3, CACNA1C, PRKAR1B, PRKAR2B]	(<0.01)
		NGF signaling pathway in apoptosis and survival	[AKT3, CAD, GAB1]	(<0.01)
		Transcription involving CREB pathway		(<0.01)
		Role of activin A in cell differentiation and proliferation	[ADCY2, ADCY5, CREB1, NR5A1/SF1, PRKAR1B, PRKAR2B]	(<0.01)
		GH-RH signaling	[ADCY2, ADCY5, CACNA1C, CREB1, PRKAR1B, PRKAR2B]	(<0.01)
		ZNF202 in regulation of expression of genes involved in atherosclerosis	[ADRB3, APOL2, LPL]	(<0.01)
		Regulation of lipid metabolism by niacin and isoprenaline	[ADCY2, ADCY5, ADRB3, PRKAR1B, PRKAR2B]	(<0.01)
		Ligand-independent activation of ESR1 and ESR2	[ADCY2, ADCY5, AKT3, PRKAR1B, PRKAR2B]	(<0.01)
		Relaxin signaling pathway	[ADCY5, AKT3, CREB1, PRKAR1B, PRKAR2B]	(<0.01)
		Melanocyte development and pigmentation	[AKT3, CREB1, PRKAR1B, PRKAR2B, PRKG1]	(<0.01)
	*Down-regulated [18]*	Immune response involving antigen presentation by MHC class I	[PDIA3]	(<0.01)
		Vitamin B6 metabolism	[PHPT1]	(<0.02)
		Blood coagulation and platelet degranulation	[F13A1]	(<0.03)
		Cholesterol and sphingolipids intracellular transport	[PPIA]	(<0.03)
		GSL metabolism		(<0.05)
**Eutopic**	*Up-regulated [19]*	Cell cycle at initiation of mitosis and regulation of G1/S transition	[LMNB2, PPP2R3A]	(<0.01)
		Dopamine D2 receptor transactivation of PDGF receptor	[PPP2R3A]	(<0.01)
		Apoptosis and survival involving caspase cascade, FAS signaling cascade and HTR1A signaling and anti-apoptosis by external signals via NF-kB	[LMNB2, PPP2R3A]	(<0.01)
		G-protein signaling involving regulation CDC42 activity	[ARHGAP17]	(<0.01)
		Gultamate regulation of Dopamine D1A receptor signaling	[PPP2R3A]	(<0.01)
		PKA signaling	[PPP2R3A]	(<0.02)
	*Down-regulated [56]*	Translation involving regulation of ElF2	[PPP1CC]	(0)
		DNA damage involving NHEJ mechanisms of DSBs repair	[CSNK2A2]	(<0.02)
		Cytoskeleton remodeling involving activin A	[FNTA]	(<0.02)
		Olfactory transduction	[OR2H1]	(<0.02)
		Cell cycle involving chromosome condensation in prometaphase, sister chromatic cohesion, regulation of S phase and initiation of mitosis	[HIST1H1C]	(<0.03)
		Cadherin mediated cell adhesion	[PTPRF]	(<0.03)
		GABA-A receptor mediated neurophysiological process	[PPP1C]	(<0.03)
		MAG-dependent inhibition of neurite outgrowth	[MAG]	(<0.05)
		Cell adhesion via PLAU signaling	[CSNK2A2]	(<0.05)
**Ectopic**	*Up-regulated [0]*			
	*Down-regulated [91]*	G-protein signaling involving Rap2A regulation pathway and G-protein alpha-s signaling cascade	[PRKAR2B, RAPGEF3]	(0)
		Glycolysis and gluconeogenesis	[ENO2]	(0)
		cAMP-Ca+2-dependent signal transduction	[PRKAR2B, RAPGEF3]	(<0.01)
		G protein mediated regulation of MAPK-ERK signaling	[PRKAR2B, RAPGEF3]	(<0.01)
		Development involving MAG, PACAP signaling, activin A, A2A and A2B receptor signaling and Hedgehog signaling	[MYH14, NANOG, NTF3, PRKAR2B, RAPGEF3]	(<0.01)
		Regulation of eNOS activity	[PRKAR2B]	(<0.01)
		CCR3 signaling in eosinophils	[FGR, MYH14]	(<0.02)
		NMDA dependent neurophysiological process	[PRKAR2B, RAPGEF3]	(<0.03)
				(<0.03)
		CFTR expression, maturation and activity	[HSPA6, PRKAR2B]	(<0.03)

^a^ > 3-fold at P < 0.01.

### K-mean clusters and differential co-expression (DC)

**Figure 4:**
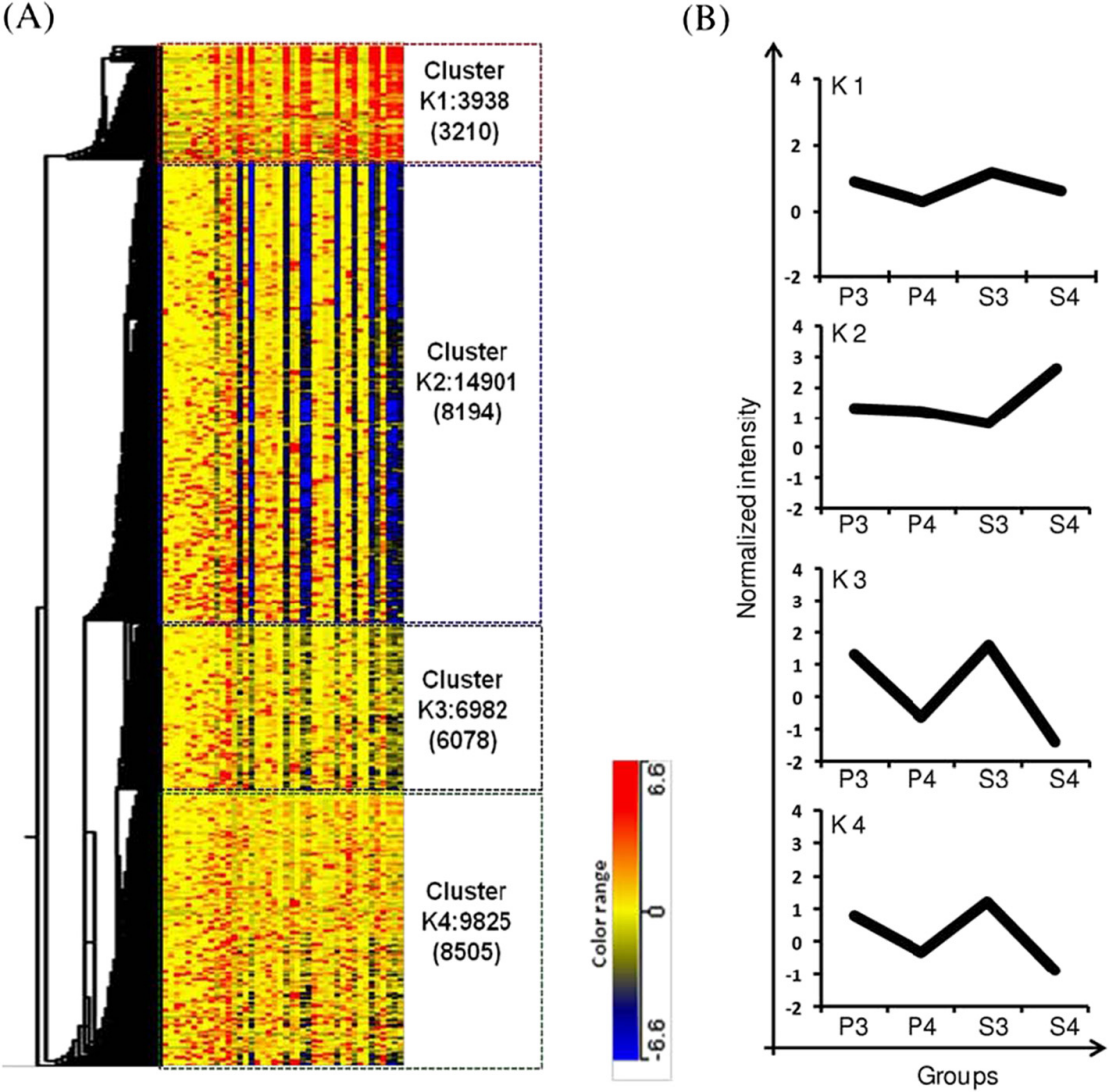
**K-means classification for all the expressed probes across eighteen (18) autologous, paired eutopic and ectopic endometrial samples.** The groups of genes showing similar expression pattern across the samples are clustered in four clusters (K1-K4) (**A**). Cluster K1 contains 3938 probes (3210 genes), cluster K2 contains 14901 probes (8194 genes), cluster K3 contains 6982 probes (6078 genes) and cluster K4 contains 9825 probes (8505 genes). The right panel (**B**) shows the average expression patterns of co-expressed probes each cluster across the annotated groups.

As shown in Figure 4, K-mean cluster analysis identified four clusters of expression patterns and profiles based on normalized hybridization signals for all expressed genes in all samples. The genes in cluster 1 (K1) did not show any specific expression pattern, while other three clusters showed overt patterns for menstrual cycle phases and severity stages. A large number of genes belonging to cluster 2 (K2) showed over-expression in severity stage 4 secretory phase endometrium (Fig. 4B). The co-expressed genes in cluster 3 (K3) and cluster 4 (K4) showed very similar patterns with an overall higher expression in stage 3 as compared to stage 4 endometrium samples irrespective of cycle phases.

Table 4 shows the pathways-based enrichment analysis of groups of genes in four (4) K-mean clusters revealing differential co-expression (DC) profiles between paired eutopic and ectopic endometrial tissues. It essentially substantiated the observation obtained from DE analysis that transcriptomic signals related to cell cycle, signal transduction, cytoskeleton remodeling, apoptosis and survival, chemotaxis, cell adhesion, and immune response were affected in the pathogenesis process of endometriosis.

**Table 4:** Estimates and description of top scored enriched categories of differentially regulated (eutopic-to-ectopic) co-expressed (DC) genes

**Cluster identity (Total number of genes) [Number of genes with differential display: Up-regulated/Down-regulated]**	
**Enriched category (p-value)**	**Gene symbols of major candidate genes (Vector of differential)**
**Cluster 1, K1 (3210) [208: 120/88]**	
Cell cycle (<0.01)	CCNB1, CDC45L, CENPF, NCAPD3, RAD51 (up);
	CCND2, MYL9, ORC6L, TNPO1 (down)
IGF-1 receptor signalling (<0.01)	IGF2 (up), IGFBP5, CCND2 (down)
Cytoskeleton remodelling involving regulation of actin (<0.02)	MYH9, MYL9 (down)
O-glycan biosynthesis (<0.02)	GALNT1, GALNT7 (down)
Immune response(<0.02)	PGR (up), CCND2 (down)
G-protein signalling in TC21 regulation pathway (<0.02)	RRAS2 (up), RASGRP2 (down)
DNA damage (<0.03)	NBN, RAD51 (up), CCND2 (down)
Neurite outgrowth (<0.05)	MYH9, MYL9 (down)
Regulation of glucose and lipid metabolism (<0.05)	CYP51A1, FOXA (up), TNPO1 (down)
G-protein signalling in Rap1 regulation pathway (<0.05)	KRIT1 (up), RASGRP2 (down)
Progesterone action (<0.05)	CCNB1, PGR (up)
Signal transduction:	
Erk interactions (<0.04)	DUSP4 (up)
AKT signaling (<0.05)	HSP90AA1 (up), CCDD2 (down)
**Cluster 2, K2 (8194) [148: 0/148]**	
Immune response (0)	ACTG1, C3, C4B, C1QB, HLA-C, PTPN11/SHP2 (down)
Cell adhesion (0)	ACTG1, ITGB1, PTPN11/SHP2 (down)
Regulation of CFTR (0)	ACTG1, HSPA8, PSMB1, TSG101, UBC (down)
Signal transduction involving activin A signalling regulation (0)	BAMBI, H3F3A, UBC (down)
Slit-Robo signaling (<0.01)	ACTG1, ROBO3 (down)
Cytoskeletal remodelling (<0.01)	ACTG1, DSTN, RALGDS (down)
Glutathione metabolism (<0.01)	GSTM5, MGST3 (down)
Chemotaxis (<0.02)	ACTG1, GNG4, ITGB1 (down)
Apoptosis and survival (<0.02)	HSPA8, ACTG1 (down)
Glucose and lipid metabolism (<0.03)	APOE (down)
	
Neurite outgrowth (<0.03)	ACTG1, DSTN (down)
N-glycan biosynthesis (<0.04)	GALT, GANAB (down)
**Cluster 3, K3 (6078) [102: 102/0]**	
Differentiation (0)	PRKCG (up)
Transcriptional regulation of amino acid metabolism (0)	MAX, PRKCG (up)
Androgen receptor nuclear signaling (<0.01)	NCOA2/GRIP1, WNT16 (up)
Neurophysiological process associated with PGE2-induced pain processing (<0.01)	GLRA3 (up)

### Gene-set enrichment analysis (GSEA)

Table 5 provides a summary of the results of GSEA implementation on co-expressed genes with differential display (DC) in the four K-mean clusters. In K4, one (1) cytoband i.e. C1 set and two (2) gene ontology i.e. C5 sets were selected. More over, three (3) DC gene sets – one each in K1, K2 and K4, respectively – were selected under BROAD regulatory gene motif sets, C3. It is notable that two (2) selected regulatory motif sets belonging to K1 and K2 were significantly (p < 0.0001) associated with ectopic sample as evident from their negative normalized enrichment scores (NES). Further, four (4) DC gene sets – two (2) each in K1 and K4, respectively – were selected under BROAD cancer gene neighborhood sets, C4. Table 5 also shows the major gene families selected in GSEA and names of the genes showing differential display in the comparison between eutopic and ectopic endometrium.

**Table 5:** Summary of GSEA results for K-mean clusters

**BROAD gene sets (Set description)**		
**Cluster identity**	**Number of selection (set identity; p value; qFDR; NES)**	**Total number of genes1; number of genes in classified gene families1,2**
		
**C1 (Cytogenetics)**		
K1	0	
K2	0	
K3	0	
K4	1 (Chr4q12; 0; 0.11; 1.8)	47; 6 (Cgf); 1 (Ong); 2 (Tf)
**C2 (Functional)**		
K1	0	
K2	0	
K3	0	
K4	0	
**C3 (Regulatory motif)**		
K1	1 (RRCCGTTA_UN; 0; 0.25; -1.8);	55; 1 (Cgf); 2 (Cdm); 2 (Ong); 3 (Pk); 11 (Tf)
K2	1 (V$AP4_q6; 0; 0.05; −2.0)	173; 1 (Cdm); 9 (Cgf); 7 (Hdp); 9 (Ong); 11(Pk);
		34 (Tf); 1 (Ts)
K3	0	
K4	1 (GCAAGA,MIR-431; 0; 0.04; 1.9)	39; 1 (Cdm); 2 (Cgf); 1 (Hdp); 2 (Ong); 1 (Pk); 6 (Tf); 2 (Ts)
**C4 (Cancer gene neighborhood)**		
K1	2 (MORF_MYC; (0; 0.00; 2.3)	72; 1 (Cdm); 2 (Cgf); 5 (Hdp); 5 (Onc); 4 (Pk); 14 (Tf)
	(MORF_ESR1; 0; 0.04; 2.0)	160; 3 (Cdm); 7 (Cgf); 7 (Hdp); 8 (Ong); 9 (Pk); 36 (Tf); 3 (Ts)
K2	0	
K3	0	
K4	2 (MORF_RAD23B; 0; 0.18; 1.8)	164; 1 (Cgf); 2 (Ong); 1 (Pk); 8 (Tf); 2 (Ts)
	(GNF2_CCNA2; 0.02; 0.23; 1.8)	62; 1 (Cdm); 6 (Pk); 3 (Tf); 1 (Ts)
**C5 (Gene ontology)**		
K1	0	
K2	0	
K3	0	
K4	2 (PATTERN_ BINDING; 0; 0.07; 2.1)	45; 2 (Cdm); 6 (Cgf); 1 (Tf)
	(POLYSACCHARIDE_BINDING; 0; 0.18; 1.9)	36; 6 (Cgf); 1 (Tf)

^1^Archived from GSEA data base [15].

^2^Based on common feature in homology and biochemical activities, not necessarily having common origin. FDR ≤0.25, minimally 10 genes in a set and maximum 1000 permutation. NES, Normalized enrichment score. The major gene families in selected gene sets in GSEA are: ***Cdm, cell differentiation markers*** (CD6, CD14, CD34, HMMR, NRCAM, PSG1, PVR, PVRL3, TLR2, and TNFSF4); ***Cgf, cytokines and growth factors*** (BMP10, CXCL5, FGF8, FGF10, FGF12, GMFB, HBEGF, HDGF, HTN3, IL13, IL16, INHA, LTBP4, MDK, MLN, NLN, NPFF, NPPA, NRG2, NRTN, PDGFRA, PF4, PF4V1, PGF, PPBP, PROK2, STC2, TNFSF4, TOR2A, VEGFA, VGF, and XCL2); ***Hdp, homeodomain proteins*** (BARX2, HOXA6, HOXB5, HOXC4, HOXC12, HOXD4, LBX1, LHX8, MSX1, PAX7, PAX8, PITX3, POU6F1, SIX3, and SIX5); Ong, oncogenes (ACT, ARNT, BCL2, BCL11B, BFA2T3, CNBP, ELF4, ERC1, EWSR1, GAS7, LCK, MSI2, MYC, NCKIPSD, NR4A3, PAX7, PAX8, PDGFRA, PICALM, RUNX1, TPM3, and TRIM24); ***Pk, protein kinases*** (ADRBK1, AURKA, AURKB, BUB1B, CAMK2G, DAPK2, EPHA2, ERN1, FLT1, GRK4, IKBKE, KSR2, LCK, MAP3K13, MAPK2K4, MAPK6, MELK, MYLK, NRK, OBSCN, PLK1, PRKACA, PRKCQ, PTK7, RPS6KB2, SNG1, STK16, STK17A, TRIM24, TTK, and WEE1); ***Tf, transcription factors*** (AFF2, ARNT, ATF7IP, ATRX, BACH1, BARX2, BCL11B, BRD1, CBFA2T3, CBX6, CLOCK, CNBP, COPS5, CREB3L1, EGR3, ELF4, EP400, ESR1, ESRRA, ETB3, EYA1, EYA3, EZH2, EZH2, FOSL1, FOSL2, FOXD3, FOXM1, GATA3, HDSE4, HMGB2, HMGN2, HOXA6, HOXB5, HOXC4, HOXC12, HOXD4, ID1, ID3, ILF3, IRF2, KLF13, LBX1, LHX8, MAZ, MBD4, MGA, MORF4L2, MSX1, MTA1, MYC, NEUROG3, NF1, NFIA, NFIC, NFRKB, NFX1, NR1I2, NR3C2, NR4A3, PAX7, PAX8, PAX9, PAXIP1, PHTF1, PIAS2, PITX3, POU6F1, PTF3L1, REST, RUNX1, RXRG, SART3, SF1 (NR5A1), SIX3, SIX3, SIX5, SMAD4, SMARCA2, SMARCA5, SOX12, SP3, TAF2, TBX15, TBX19, TBX5, TCF7, TFAP4, TFDP2, TRIM24, UBTF, VPS72, WT1, ZBTB22, ZNF133, ZNF134, ZNF146, ZNF157, ZNF238, and ZNF592); ***Ts, tumor suppressors*** (BRCA1, BUB1B, GATA3, MAPK2K4, NF1, SDHC, SMAD4, WT1, and XPC). The genes that showed differential expression between eutopic and ectopic samples in the present study are marked by underlines.

### Expressional cohort of marker genes

Table 6 provides the list of selected twenty eight (28) genes that appeared significant from combined analysis of GSEA-selected gene sets followed by DC analysis and from DE analysis of microarray data of 18 paired samples. Table 6 also shows that the validity of the prediction value of the expressional cohort based on quantitative analysis in a different set of autologous, paired eutopic and ectopic samples obtained from a separate group of 8 subjects was markedly high.

**Table 6:** Selected genes expression of which bear predictable leads to ovarian endometriosis among Indian women

**Expression characteristics**		
**Gene symbol (GenBank ID) [Gene name]**	**Fold change**^**a**^	
	**Microarray**^**b**^	**RT-PCR**^**c**^
**Up-regulated in eutopic tissue**		
General		
3HMGN2 (NM_005517)	4.8+1.0	2.4++0.3
*[High-mobility group nucleosomal binding domain 2]*		
MKI67 (NM_002417 )	3.4+0.9	4.6+1.8
*[Ki-67-like antigen]*		
NRCAM (NM_001037132)	4.5+1.6	2.1+0.5
[Neuronal cell adhesion molecule]*		
PARG (NM_003631)	3.8+2.1	3.4+1.5
*[Poly (adp-ribose) glycohydrolase]*		
TMPO (NM_001032283)	3.1+1.9	2.8+1.1
*[Thymopoietin]*		
Stage 3		
ATP2A2 (NM_001681)	5.1+1.9	8.6+3.5
*[ATPase, Ca++ transporting, cardiac muscle,slow twitch 2]*		
CHIA (NM_021797)	3.4+1.3	7.3+1.5
*[Chitinase, acidic]*		
DAPK2 (NM_014326)	6.2+2.3	9.7+1.8
[Death-associated protein kinase 2]*		
ERC1 (NM_178040)	6.8+1.5	10.8+3.0
[Elks/rab6-interacting family member 1]*		
TACC2 (NM_206862)	13.7+3.2	6.4+2.0
*[Transforming, acidic coiled-coil containing protein 2]*		
ZBTB22 (NM_005453)	5.2+2.7	12.3+3.1
[Zinc finger and BTB domain containing 22]*		
Proliferative phase		
BAG5 (NM_001015049)	4.2+1.9	6.5+4.1
*[Bcl2-associated athanogene 5]*		
CDCA3 (NM_031299)	3.9+1.5	9.9+6.6
*[Cell division cycle associated 3]*		
EGR3 (NM_004430)	3.7+1.0	5.3+1.4
[Early growth response 3]*		
FGFBP1 (NM_005130)	5.7+2.1	7.3+2.7
*[Fibroblast growth factor binding protein 1]*		
TPM3 (NM_001043352)	3.4+1.9	6.3+3.4
*[Tropomyosin 3]*		
Secretory phase		
DHRS3 (NM_004753)	3.6+1.7	5.9+1.9
*[Dehydrogenase/reductase (SDR family) member]*		
**Up-regulated in ectopic tissue**		
General		
BAP1 (NM_004656)	12.6+3.3	13.9+3.4
[BRCA1 associated protein-1]*		
CBLL1 (NM_024814)	6.2+2.5	6.1+2.5
*[Cas-Br-M (murine) ecotropic retroviral transforming sequence-like 1]*		
CLOCK (NM_004898)	9.0+2.6	5.3+0.6
[Clock homolog (mouse)]*		
EPB41L1 (NM_012156)	3.1+1.6	2.6+1.2
*[Erythrocyte membrane protein band 4.1-like 1]*		
LPL (NM_000237)	5.5+2.9	7.2+2.9
*[Lipoprotein lipase]*		
PPAT (NM_002703)	5.8+2.9	5.6+3.4
*[Phosphoribosyl pyrophosphate amidotransferase]*		
SCAMP1 (NM_004866)	10.5+3.2	6.0+2.6
*[Secretory carrier membrane protein 1]*		
SFRS1 (NM_001078166)	8.7+3.6	6.6+2.2
*[Splicing factor, arginine/serine-rich 1]*		
USP46 (NM_022832)	4.0+1.8	3.7+1.1
*[Ubiquitin specific peptidase 46]*		
YWHAE (NM_006761)	3.4+2.7	3.3+1.8
*[Tyrosine 3-monooxygenase/tryptophan 5-monooxygenase activation protein, epsilon]*		
ZNF644 (NM_201269)	4.4+1.0	5.1+2.9
*[Zinc finger protein 644]*		

^a^Between eutopic and ectopic samples, shown as mean ± SD.

^b^Based on GSEA implementation on DC results and their DE analysis (n = 18).

^c^Based on transcript numbers from RT-PCR analysis of RNA samples from a different set of autologous paired eutopic and ectopic tissues (n = 8).

*Genes selected in gene families identified in GSEA gene sets (see Table 5).

## Discussion

The awareness that whole genome expression array analysis may yield high dimension knowledge towards deciphering patho-etiology of complex diseases [20,21] has prompted several groups of investigators to employ this approach to examine the transcriptomics basis of endometriosis using eutopic and ectopic samples [3-9]. Although significant and interesting observations have emerged from these reports, these studies did not include the possible impact of one or more of the factors like the demographic characteristics, position of endometriosis, fertility history, severity stages and phases of menstrual cycle influencing the genomic expression in eutopic and ectopic tissues [2,8,22]. In the present study, we have examined the whole genome transcriptomics of autologous, paired eutopic and ectopic samples obtained from fertile Indian women with ovarian endometriosis of known clinical severity and phases of menstrual cycle but with no history of previous treatment for endometriosis at the time of tissue collection. We analyzed the expression profiles to delineate the impact of stages of severity and phases of cycle in eutopic and ectopic samples. We observed that clustering effect on expression arrays was maximum in paired samples, followed by stages of clinical severity and positional cue. The phase of menstrual cycle exhibited minimal clustering effect on expressional profiles in the experimental samples.

Generally, we observed that eutopic tissue yielded a normal frequency distribution histogram of gene expressions for different levels of expression and that an overall higher numbers of genes in eutopic endometrium expressed higher transcriptomic signals as compared to ectopic samples; ectopic samples yielded a truncated frequency distribution histogram. Furthermore, higher numbers of genes bearing expression levels at the high and at the low ends of the frequency distribution were observed in ectopic tissues as compared to eutopic tissues. We believe that implementation of appropriate computational models based on Shannon’s noise-signal entities and probability of size of loss of signals may yield in the future new leads about the global genomic expression pattern in the ectopic tissue [23,24]. It is notable in this regard that: (i) a large number (~0.7 K) of genes were silenced in the ectopic tissue at stage 4 condition as compared to stage 3, and (ii) expressional clustering cohesion was very high (cd: 0.07) between the eutopic and ectopic endometrium in stage 4 disease condition. Taken together, it is suggestive of high degree of pathognomonicity in stage 4 eutopic endometrium [8,25].

Major highlights in the previous studies on large scale expressional array analysis were to explore the gene-specific DE in paired analysis between eutopic and ectopic endometrium with an assumption that a 2-fold change at P < 0.05 between two groups of tissue samples was sufficiently significant for further analysis. As pointed out elsewhere, this may give rise to different sets of biases and inadequacies in interpretation and discovery [26,27]. To circumvent these acknowledged insufficiencies, we have employed a 3-fold change at P < 0.01 as the pre-set filter for DE of individual genes between groups followed by pathway networks based enrichment analysis, and for DC analysis of K-mean based expressional clusters followed by gene set enrichment analysis (GSEA) model [16] to interpret the present transcriptomics data.

*Post-hoc* analysis of expressional signals in eutopic and ectopic tissues under different sets of categorical comparison revealed that several signaling pathways related to immune response were commonly affected in eutopic and ectopic endometrium. The results from the present study support the observation made by Zhao et al. based on GSEA of archival transcriptomics data sets of endometriosis that the main canonical pathways putatively involved in the process of endometriosis were related to that of immune and inflammatory diseases [27]. Additionally, we hypothesize from the results of the present study that functional connectivity between over-expression of CLOCK and inflammatory disorder, as well as, between over-expression of genes associated with lipid metabolism and inflammation at the local tissue level are operative in the pathogenesis of endometriosis [28-30].

We also observed that neuronal processes involving nNOS signaling pathways and GABA synthesis and metabolism were commonly expressed in both tissue types and a large number of genes involving several signaling pathways (corticoliberin, opioid receptors, serotonin receptors, melatonin, dopamine receptor, neuronal cell adhesion, NGF) associated with neurophysiological processes were up-regulated in stage 3 ectopic endometrium. Earlier the possible involvement of neuroendocrine processes in the pathogenesis of endometriosis has been implicated [31-34]. Pathways and networks based enrichment analysis of DE of individual genes and DC of gene cohorts in four clusters in the present study revealed that expression of genes in pathways directly and indirectly associated with cell apoptosis and survival, cytoskeleton remodeling, chemotaxis and cell adhesion were differentially affected in eutopic and ectopic samples. Involvement of these pathways in endometriosis has earlier been reported by several groups based on different experimental models [35-38].

The pathogenesis of endometriosis has also been associated with excessive production of estrogens by up-regulated expression of aromatase and 17β-HSD type 1, and suppression of 17β-HSD types 2 and 4, collectively resulting in an increased ratio of estradiol-17β to estrone in ectopic tissues [39-42]. It has however been reported by others that mRNA and protein expressions of aromatase were minimal in ectopic tissues [42,43]. Our transcriptomics data also failed to identify any overt change in the expression of genes for aromatase (CYP19A1) and 17β-HSD (HSD17B1-B17) in endometriosis. However, our observation that genes (NR5A1, STAR) for steroidogenic factor (SF)1 and steroidogenic acute regulatory protein (StAR), which are known to be significant regulators of steroidogenesis [44,45] were highly expressed in the ectopic endometrium substantiates previous reports [46-48].

An over-expression of ERBB3 in proliferative phase eutopic endometrium and secretory phase ectopic endometrium was seen in the present study; it has been associated with ligand-independent activation of estrogen receptors (ESR1 and ESR2) in target tissues [49]. However, ERBB3 was up-regulated in eutopic tissues as compared to ectopic tissues in pooled analysis. The activation of ESRs and relative down-regulation of progesterone receptor (PGR) in the secretory phase ectopic endometrium is suggestive of relative suppression of progesterone action in the ectopic endometrium. Indeed, the phenomenon of progesterone resistance in the ectopic endometrium has earlier been documented by other groups [8,30,50].

**Figure 5:**
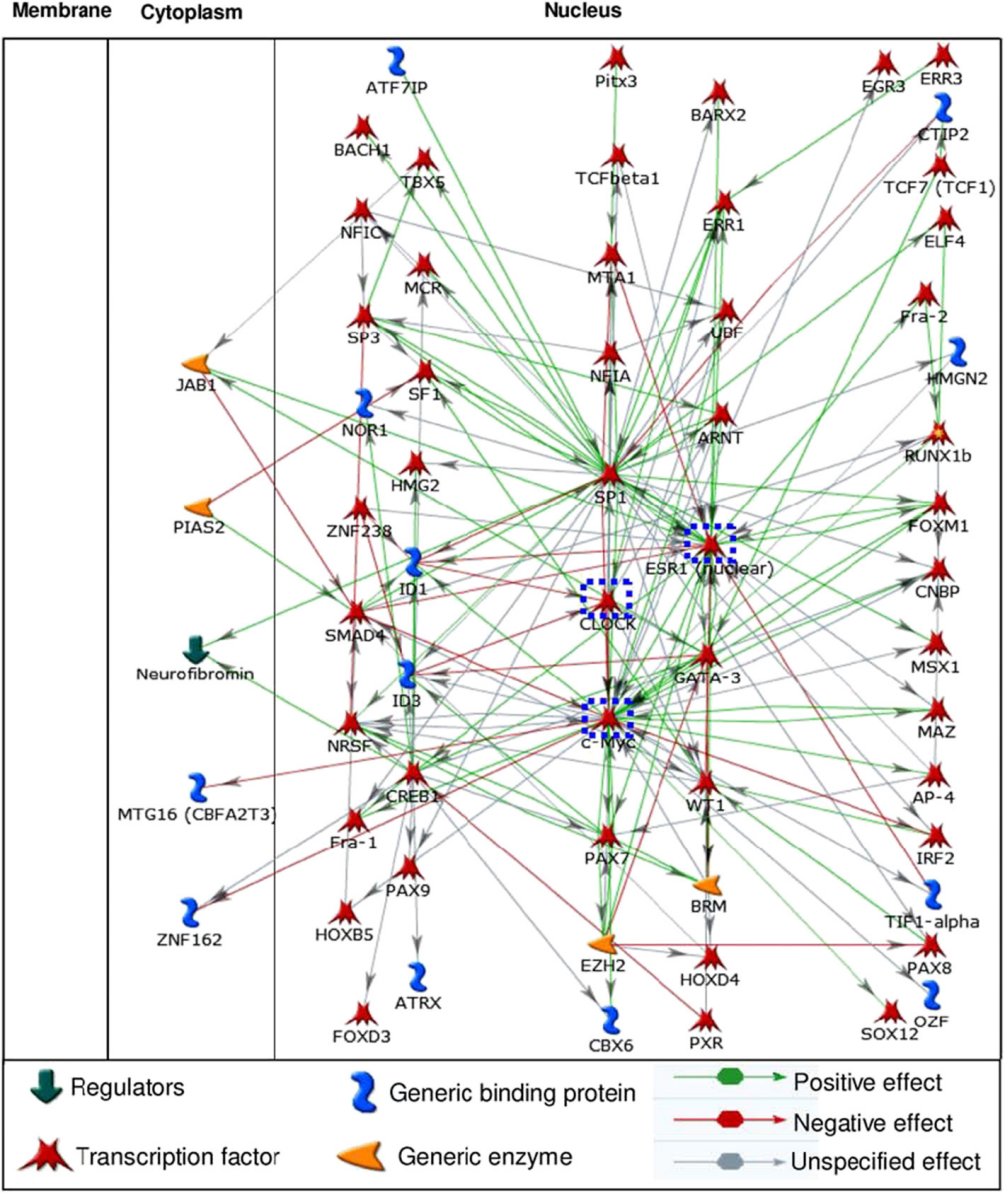
**Knowledge-based construction of the pathways-network of transcription factors putatively associated with pathogenesis of endometriosis.** The transcription factors were identified from GSEA implementation on co-expressed genes. It is notable that CLOCK, ESR1, and MYC (shown inside *blue dotted rectangle*) are differentially co-expressed in endometriosis as shown is Table 5.

Collectively, it appears that the results of our study corroborate well with the previous reports and lead us to hypothesize that eutopic endometrium which is transcriptionally dysfunctional in mediating immune-neuro-endocrine responses may bear vulnerability to give rise to endometriotic lesion if deposited ectopically. Subsequently, the ectopically placed endometrial tissue with positional input and under fluctuating levels of sex steroid hormones in vulnerable subjects may develop differential expression repertoire related to cell survival, adhesion, migration and growth resulting in endometriotic lesion [51,52]. More over, it appears that a pathways-network of several transcription factors including CLOCK-ESR1-MYC may be involved at the transcriptomic level towards pathoetiology of ovarian endometriosis (Fig. 5). Further studies are warranted to test this hypothesis.

Endometriosis, by definition, is a benign disease, however, there are a few reports indicating risk of malignant transformation in endometriosis [53-55]. In the present study, we observed a general suppression in the expression of genes associated with cell cycle and DNA damage repair in both eutopic and ectopic endometrium in fertile women with endometriosis. Interestingly, there is a recent report indicating a better survival rate for women with endometriosis for all malignancies combined, and specifically for ovarian and breast cancer, while it was poorer in malignant melanoma [56]. While the present results revealing the lack of overt oncogenic potential in endometriotic tissue concur with some of the earlier reports [6-8], genes (CHEK1, ERBB family, laminin gamma and Ki-67) associated with gynecological cancers [57-60] were highly expressed in autologous, paired eutopic and ectopic tissues. Thus, the possibility of inducement of oncogenic transformation through critical phase transition [61] in the course of endometriosis disease progression cannot be ruled out, especially in the high risk population [62,63].

Finally, we have identified for the first time a cohort of twenty-eight (28) genes with high degree of predictability index for ovarian endometriosis in fertile women. We believe this cohort of genes can be used for further study to discover patho-physiology of ovarian endometriosis.

## Conclusions

Expressional profiles between paired eutopic and ectopic samples showed markedly higher cohesion compared with that of clinical stages of severity and phases of menstrual cycle. Endometriotic endometrium displayed anomalous expressional balance for several genes associated with immunological, neuracrine and endocrine functions. Although no overt oncogenic potential in endometriotic tissue was observed, expressions of a few genes (CHEK1, ERBB family, laminin gamma and Ki-67) associated with gynecological cancers were seen to be up-regulated. A novel cohort of twenty-eight (28) genes representing potential marker for ovarian endometriosis in fertile women was discovered.

## Competing interests

The authors declare that they have no competing interests.

## Authors’ contributions

MAK contributed in performing experiments, data acquisition and analysis. JS and DG contributed to the conception, designing, acquisition, analysis and interpretation of data and the drafting process of the manuscript. SM contributed to the sample acquisition, and results interpretation process of the manuscript. All authors read and approved the final manuscript.

## Supplementary Material

Additional file 1 **Table S1.** Summary of subject profiles. Click here for file

Additional file 2 **Table S2.** Primers used in real-time PCR reactions. Click here for file

Additional file 3 **Table S3.** List of differentially regulated genes. Click here for file

## References

[B1] Bulun SE Endometriosis New Engl J Med 2009 360 268 279 10.1056/NEJMra0804690 19144942

[B2] Rogers PAW D’Hooghe TM Fazleabas A Gargett CE Giudice LC Montgomery GW Rombauts L Salamonsen LA Zondervan KT Priorities for endometriosis research: recommendations from an International consensus workshop Reprod Sci 2009 16 335 346 19196878 10.1177/1933719108330568 PMC3682634

[B3] Eyster KM Boles AL Brannian JD Hansen KA DNA microarray analysis of gene expression markers of endometriosis Fertil Steril 2002 77 38 42 10.1016/S0015-0282(01)02955-7 11779588

[B4] Eyster KM Klinkova O Kennedy V Hansen KA Whole genome deoxyribonucleic acid microarray analysis of gene expression in ectopic versus eutopic endometrium Fertil Steril 2007 88 1505 1533 10.1016/j.fertnstert.2007.01.056 17462640

[B5] Hever A Roth RB Hevezi P Marin ME Acosta JA Acosta H Rojas J Herrera R Grigoriadis D White E Human endometriosis is associated with plasma cells and over expression of B lymphocyte stimulator Proc Natl Acad Sci (USA) 2007 104 12451 12456 10.1073/pnas.0703451104 17640886 PMC1941489

[B6] Borghese B Mondon F Noel J Fayt I Mignot TM Vaiman D Chapron C Gene expression profile for ectopic versus eutopic endometrium provides new insights into endometriosis oncogenic potential Mol Endocrinol 2008 22 2557 2562 10.1210/me.2008-0322 18818281

[B7] Zafrakas M Tarlatzis BC Streichert T Pournaropoulos F Wolfle U Smeets SJ Wittek B Grimbizis G Brakenhoff RH Pantel K Genome-wide microarray gene expression, array-CGH analysis, and telomerase activity in advanced ovarian endometriosis: a high degree of differentiation rather than malignant potential Int J Mol Med 2008 21 335 344 18288381

[B8] Khan MA Sengupta J Giudice LC Mittal S Kumar S Datta Gupta S Sharma R Najwa AR Ghosh D cDNA-based transcript analysis of autologous eutopic and ectopic endometrium of women with moderate and severe endometriosis J Endometriosis 2011 3 8 33

[B9] Wu Y Kajdacsy-Balla A Strawn E Basir Z Halverson G Jailwala P Wang Y Wang X Ghosh S Guo SW Transcriptional characterizations of differences between eutopic and ectopic endometrium Endocrinology 2006 147 232 246 16195411 10.1210/en.2005-0426

[B10] Cornillie FJ Oosterlynck D Lauweryns JM Koninckx PR Deeply infiltrating pelvic endometriosis: histology and clinical significance Fertil Steril 1990 53 978 983 2140994 10.1016/s0015-0282(16)53570-5

[B11] Vercellini P Frontino G Pietropaolo G Gattei U Daguati R Crosignani PG Deep endometriosis: definition, pathogenesis and clinical management J Am Assoc Gynecol Laparosc 2004 11 153 161 10.1016/S1074-3804(05)60190-9 15200766

[B12] American Society for Reproductive Medicine Revised classification of endometriosis 1996 Fertil Steril 1997 67 817 818 10.1016/S0015-0282(97)81391-X 9130884

[B13] Ghosh D Sharkey AM Charnock-Jones DS Smith SK Sengupta J Effect of low-dose mifepristone administration on day 2 after ovulation on transcript profiles in implantation-stage endometrium of rhesus monkeys Reproduction 2009 138 357 370 10.1530/REP-08-0442 19439560

[B14] Pan W A comparative review of statistical methods for discovering differently expressed genes in replicated microarray experiments Bioinformatics 2002 18 546 554 10.1093/bioinformatics/18.4.546 12016052

[B15] http://www.broadinstitute.org/gsea/msigdb

[B16] Subramanian A Tamayo P Mootha VK Gene set enrichment analysis: A knowledge-based approach for interpreting genome-wide expression profiles Proc Natl Acad Sci (USA) 2005 102 15545 15550 10.1073/pnas.0506580102 16199517 PMC1239896

[B17] Pfaffl MW Horgan GW Dempfle L Relative expression software tool (REST) for group-wise comparison and statistical analysis of relative expression results in real-time PCR Nucleic Acid Res 2002 30 e36 1–10 10.1093/nar/30.9.e36 11972351 PMC113859

[B18] Bustin SA Quantification of mRNA using real time reverse transcription PCR (RT-PCR): trends & problems J Mol Endocrinol 2002 29 23 39 10.1677/jme.0.0290023 12200227

[B19] http://www.ncbi.nlm.nih.gov/geo/GSE37837

[B20] Giudice LC Elucidating endometrial function in the post-genomic era Hum Reprod Update 2003 9 223 235 10.1093/humupd/dmg019 12859044

[B21] Shai RM Microarray tools for deciphering complex diseases Front Biosci 2006 11 1414 1424 10.2741/1892 16368525

[B22] Bulun SE Adashi EY Larsen PR, Kronenberg HM, Melmed S, Polonsky K The physiology and pathology of the female reproductive axis Williams Textbook of Endocrinology 2003 10 WB Saunders, Philadelphia 587 664

[B23] Cover TM Thomas JA Elements of Information Theory 1991 Wiley, New York

[B24] Zhou T Carlson JM Doyle J Mutation, specialization, and hypersensitivity in highly optimized tolerance Proc Natl Acad Sci (USA) 2002 99 2049 2054 10.1073/pnas.261714399 11842230 PMC122317

[B25] Aghajanova L Giudice LC Molecular evidence for differences in endometrium in severe versus mild endometriosis Reprod Sci 2011 18 229 251 10.1177/1933719110386241 21063030 PMC3118406

[B26] Shi J Walker MG Gene set enrichment analysis (GSEA) for interpreting gene expression profiles Curr Bioinformtics 2007 2 133 137 10.2174/157489307780618231

[B27] Zhao H Wang Q Bai C He K Pan Y A cross-study gene set enrichment analysis identifies critical pathways in endometriosis Reprod Biol Endocrinol 2009 7 94 10.1186/1477-7827-7-94 19735579 PMC2752458

[B28] Bellet MM Sassone-Corsi P Mammalian circadian clock and metabolism – epigenetic link J Cell Sci 2010 123 3837 3848 10.1242/jcs.051649 21048160 PMC2972271

[B29] Sancar A Lindsey-Boltz LA Kang T-H Reardon JT Lee JH Ozturk N Circadian clock control of the cellular response to DNA damage FEBS Lett 2010 584 2618 2625 10.1016/j.febslet.2010.03.017 20227409 PMC2878924

[B30] Prieur X Roszer T Ricote M Lipotoxicity in macrophages: evidence from diseases associated with the metabolic syndrome Biochim Biophys Acta 2010 1801 327 337 10.1016/j.bbalip.2009.09.017 19796705

[B31] Tariverdian N Theoharides TC Siedentopf F Gutierrez G Jeschke U Rabinovich GA Blois SM Arck PC Neuro-endocrine-immune disequilibrium and endometriosis: an interdisciplinary approach Semin Immunopathol 2007 29 193 210 10.1007/s00281-007-0077-0 17621704 PMC2668599

[B32] Tariverdian N Rucke M Szerkes-Bartho J Blois SM Karpf EF Sedomayr H Siedentopf F Arck PC Neuro-endocrine circuitry and endometriosis: progesterone derivative dampens corticotrophin-releasing hormone-induced inflammation by peritoneal cells in vitro J Mol Med 2010 88 267 278 10.1007/s00109-009-0559-8 19898767

[B33] Wang G Tokushige N Russell P Dubinovsky S Markham R Fraser IS Neuroendocrine cells in eutopic endometrium of women with endometriosis Hum Reprod 2010 25 287 291 10.1093/humrep/dep379 19910323

[B34] Asante A Taylor RN Endometriosis: the role of neuroangiogenesis Ann Rev Physiol 2011 73 163 182 10.1146/annurev-physiol-012110-142158 21054165

[B35] Nasu K Yuge A Tsuno A Nishida M Narahara H Involvement of resistance to apoptosis in the pathogenesis of endometriosis Histol Histopathol 2009 24 1181 1192 19609865 10.14670/HH-24.1181

[B36] Gentilini D Vigano P Somigliana E Vicentini LM Vignali M Busacca M Di Blasio AM Endometrial stromal cells from women with endometriosis reveal peculiar migratory behavior in response to ovarian steroids Fertil Steril 2010 93 706 715 10.1016/j.fertnstert.2008.10.014 19022426

[B37] Stephens AN Hannan NJ Rainczuk A Meehan KL Chen J Nicholls PK Rombauts LJ Stanton PG Robertson DM Salamonsen LA Post-translational modifications and protein-specific isoforms in endometriosis J Proteome Res 2010 9 2438 2449 10.1021/pr901131p 20199104

[B38] Adachi M Nasu K Tsuno A Kawano Y Narahara H Attachment to extracellular matrices is enhanced in human endometriotic stromal cells: a possible mechanism underlying the pathogenesis of endometriosis Eur J Obstet Gynecol Reprod Biol 2010 155 85 88 21112686 10.1016/j.ejogrb.2010.10.026

[B39] Bulun SE Imir G Utsunomiya H Thung S Gurates B Tamura M Lin Z Aromatase in endometriosis and uterine leiomyomata J Steroid Biochem Mol Biol 2005 95 57 62 10.1016/j.jsbmb.2005.04.012 16024248

[B40] Dassen H Punyadeera C Kamps R Delvoux B Van Langendonckt A Donnez J Husen B Thole H Dunselman G Groothuis P Estrogen metabolizing enzymes in endometrium and endometriosis Hum Reprod 2007 22 3148 3158 10.1093/humrep/dem310 17921479

[B41] Smuc T Hevir N Ribic-Pucelj M Husen B Thole H Rizner TL Disturbed estrogen and progesterone action in ovarian endometriosis Mol Cell Endocrinol 2009 301 59 64 10.1016/j.mce.2008.07.020 18762229

[B42] Delvoux B Groothuis P D’Hooghe T Kyama C Dunselman G Romano A Increased production of 17β estradiol in endometriosis lesions is the result of impaired metabolism J Clin Endocrinol Metab 2009 94 876 883 19088158 10.1210/jc.2008-2218

[B43] Colette S Lousse JC Defrere S Curaba M Heilier JF Van Langendonckt A Mestdagt M Foidart JM Loumaye E Donnez J Absence of aromatase protein and mRNA expression in endometriosis Hum Reprod 2009 24 2133 2141 10.1093/humrep/dep199 19493871

[B44] Val P Lefrancois-Martinez AM Veyssiere G Matinez A Sf-1 a key player in the development and differentiation of steroidogenic tissues Nucl Recept 2003 1 8 10.1186/1478-1336-1-8 14594453 PMC240021

[B45] Stocco DM StAR protein and the regulation of steroid hormone biosynthesis Ann Rev Physiol 2001 63 193 213 10.1146/annurev.physiol.63.1.193 11181954

[B46] Bulun SE Utsunomiya H Lin Z Yin P Cheng YH Pavone ME Tokunaga H Trukhacheva E Attar E Gurates B Sterodogenic factor-1 and endometriosis Mol Cell Endocrinol 2009 300 104 108 10.1016/j.mce.2008.12.012 19150483

[B47] Utsunomiya H Cheng YH Lin Z Reierstad S Yin P Attar E Xue Q Imir G Thung S Trukhacheva E Upstream stimulatory factor-2 regulates steroidogenic fctor-1 expression in endometriosis Mol Endocrinol 2007 22 904 914 18165439 10.1210/me.2006-0302 PMC2276471

[B48] Tian Y Kong B Zhu W Su S Kan Y Expression of steroidogenic factor 1 (SF-1) and steroidogenic acute regulatory protein (StAR) in endometriosis is associated with endometriosis severity J Int Med Res 2009 37 1389 1395 19930843 10.1177/147323000903700513

[B49] http://www.genego.com/map_2210.php

[B50] Bulun SE Cheng YH Pavone ME Xue Q Attar E Trukhacheva E Tokunaga H Utsunomiya H Yin P Luo X Estrogen receptor-b, estrogen receptor-a, and progesterone resistance in endometriosis Semin Reprod Med 2010 28 36 43 10.1055/s-0029-1242991 20104427 PMC3073375

[B51] Wren JD Wu Y Guo SW A system-wide analysis of differentially expressed genes in ectopic and eutopic endometrium Hum Reprod 2007 22 2093 2102 10.1093/humrep/dem129 17562676

[B52] Montgomery GW Nyholt DR Zhao ZZ Treloar SA Painter JN Missmer SA Kennedy SH Zondervan KT The search for genes contributing to endometriosis risk Hum Reprod Update 2008 14 447 457 10.1093/humupd/dmn016 18535005 PMC2574950

[B53] Varma R Rollason T Gupta JK Maher ER Endometriosis and the neoplastic process Reproduction 2004 127 293 304 10.1530/rep.1.00020 15016949

[B54] Vlahos NF Kalampokas T Fotiou S Endometriosis and ovarian cancer: a review Gynecol Endocrinol 2010 26 213 219 10.3109/09513590903184050 19718562

[B55] Kurman RJ Shih IM Molecular pathogenesis and extraovarian origin of epithelial ovarian cancer – shifting the paradigm Hum Pathol 2011 42 918 931 10.1016/j.humpath.2011.03.003 21683865 PMC3148026

[B56] Melin A Lundholm C Malki N Swahn ML Sparen P Bergqvist A Endometriosis as a prognostic factor for cancer survival Int J Cancer 2011 129 948 955 10.1002/ijc.25718 20949560

[B57] Arora S Bisanz KM Peralta LA Basu GD Choudhary A Tibes R Azorsa DO RNAi screening of kinome identifies modulators of cisplatin response in ovarian cancer cells Gynecol Oncol 2010 118 220 227 10.1016/j.ygyno.2010.05.006 20722101

[B58] Concin N Hefler L van Bavel J Mueller-Holzner E Zeimet A Daxenbichler G Speiser P Hacker N Marth C Biolgical markers in pT1 and pT2 ovarian cancer with lymph node metastasis Gynecol Oncol 2003 89 9 15 10.1016/S0090-8258(02)00147-6 12694648

[B59] Katayama M Sekiguchi K Laminin-5 in epithelial tumour invasion J Mol Histol 2004 35 277 286 15339047 10.1023/b:hijo.0000032359.35698.fe

[B60] Perez-Nadales E Lloyd AC Essential function for ErbB3 in breast cancer proliferation Breast Cancer Res 2004 6 137 139 10.1186/bcr792 15084235 PMC400683

[B61] Carlson JM Doyle J Complexity and robustness Proc Natl Acad Sci (USA) 2002 99 Suppl. 1 2538 2545 11875207 10.1073/pnas.012582499 PMC128573

[B62] Prowse AH Manek S Varma R Liu J Godwin AK Maher ER Tomlinson IP Kennedy SH Molecular genetic evidence that endometriosis is a precursor of ovarian cancer Int J Cancer 2006 119 556 562 10.1002/ijc.21845 16506222

[B63] Kvaskoff M Mesrine S Clavel-Chapelon F Boutron-Rualt MC Endometriosis risk in relation to naevi, freckles and skin sensitivity to sun exposure: the French E3N cohort Int J Epidemiol 2009 38 1143 1153 10.1093/ije/dyp175 19351698

