# Supplementary material for: Genome-wide expressions in autologous eutopic and ectopic endometrium of fertile women with endometriosis
Source: Reprod Biol Endocrinol. 2012 Sep 24;10:84. doi: 10.1186/1477-7827-10-84 (PMC3533745; doi:10.1186/1477-7827-10-84)
Supplement: Additional file 1 — Table S1. Summary of subject profiles. [file 1477-7827-10-84-S1.doc]

| **Supplemental Table S1: Summary of subject profiles** |
| --- |
| __________________________________________________________________ |
| Identification Age (y) Cycle Phase of Severity Parity |
| number day^a^ cycle^b^ stage^c^ |
| ___________________________________________________________________ |
| **Samples used for exploratory analysis (n=18)** |
| E17 29 9 P 4 G1L1 |
| E20 40 13 P 4 G6L6 |
| E23 33 10 P 4 G1L1 |
| E26 45 9 P 3 G3L3 |
| E31 24 19 P 3 G1L1 |
| E32 38 9 P 3 G3L2 |
| E33 28 17 S 4 G2L1 |
| E40 25 7 P 4 G4L2 |
| E43 40 14 P 3 G1L1 |
| E48 31 8 P 4 G1L1 |
| E49 37 18 S 3 G2L2 |
| E52 30 11 P 4 G4L2 |
| E56 30 10 P 4 G1L1 |
| E57 37 24 S 3 G4L4 |
| E68 31 17 S 3 G1L1 |
| E70 34 17 S 4 G2L2 |
| E73 25 8 P 4 G1L1 |
| E75 40 10 P 3 G2L1 |
| **Samples used for prediction analysis (n=8)** |
| Ep2 33 12 P 4 G2L2 |
| Ep7 27 18 S 3 G1L1 |
| Ep9 31 10 P 4 G2L1 |
| Ep16 26 10 P 3 G1L1 |
| Ep21 38 19 S 3 G2L1 |
| Ep22 41 11 P 3 G2L2 |
| Ep23 37 17 S 4 G2L2 |
| Ep30 25 17 S 4 G4L2 |
| __________________________________________________________________ |
| ^a^based on the report obtained from the subject  ^b^based on histological reports. ^c^based on standardized protocol (12)  P, proliferative phase; S, secretory phase  3, clinical severity stage 3 (moderate); 4, clinical severity stage 4 (severe)  G, total number of pregnancy; L, number live offspring  E, samples used for exploratory analysis  Ep, samples used for prediction analysis  All (n=26) patients were referred from the Pain Clinics. |
