# Supplementary material for: Genome-wide expressions in autologous eutopic and ectopic endometrium of fertile women with endometriosis
Source: Reprod Biol Endocrinol. 2012 Sep 24;10:84. doi: 10.1186/1477-7827-10-84 (PMC3533745; doi:10.1186/1477-7827-10-84)
Supplement: Additional file 2 — Table S2. Primers used in real-time PCR reactions. [file 1477-7827-10-84-S2.doc]

| **Supplemental Table S2: Primers used in real-time PCR reactions** |
| --- |
| ______________________________________________________________________ |
| Gene name Primers sequence (5’ to 3’) T_m_ |
| [Gene symbol; |
| GenBank ID] |
| _______________________________________________________________________ |
| Ataxia telangiectasia GCAACATGATGTGAAAACCCATC (s) 57.8 |
| mutated TCACTGTAACCTGTAACCTCTGC (as) 58.7 |
| [ATM; NM_000051] |
| DDHD domain ATCCCTTATCTGTATTCCGCTGTC (s) 58.9 |
| containing 1 GCTGTCACCAACTTAGCACTAAC (as) 58.5 |
| [DDHD1; NM_030637] |
| Dynein, light chain CATTGCGGCTCATATCAAGAAGG (s) 58.8 |
| factor 1 [DYNLT1; GAAGTGTTTGGTTTCATGTGTCAC (as) 58.2 |
| NM_006519] |
| Ferritin, heavy GAACTACCACCAGGACTCAGAG (s) 58.0 |
| polypeptide 1 GCAAAGTTCTTCAAAGCCACATC (as) 58.0 |
| [FTH1; NM_002032] |
| Glyceraldehyde-3- TGGTCTCCTCTGACTTCAAC (s) 54.5 |
| phosphate GTAGCCAAATTCGTTGTCATAC (as) 54.4 |
| dehydrogenase [GAPDH; |
| NM_002046] |
| Laminin receptor 1 AGAGCCCTGGAATATGAAGTGAC (s) 58.6 |
| [LAMR1; CCTTTAAGTTACGACGGGAATCC (as) 57.9 |
| M14199] |
| Mesoderm induction TTCCTGGCTGATGAAGACAGAG (s) 58.3 |
| early response 1 GGTCCCACCATGATCTCCTTC (as) 58.1 |
| [MIER2; NM_017550] |
| WD repeat domain GCCCAGCCAAGTATCTCTTAGG (s) 58.7 |
| 87 [WDR87; GGTGCCCAGAGAATATCAGTCC (as) 58.7 |
| NM_031951] |
| ___________________________________________________________________________ |
| s, sense strand  as, anti-sense strand |
