# Supplementary material for: Genome-wide expressions in autologous eutopic and ectopic endometrium of fertile women with endometriosis
Source: Reprod Biol Endocrinol. 2012 Sep 24;10:84. doi: 10.1186/1477-7827-10-84 (PMC3533745; doi:10.1186/1477-7827-10-84)
Supplement: Additional file 3 — Table S3. List of differentially regulated genes. [file 1477-7827-10-84-S3.doc]

| **Supplemental Table S3: List of differentially^a^ regulated genes** _____________________________________________________________________________ |
| --- |
| Description of comparison |
| Specifics Symbol Gene name Fold change |
| (GenBank |
| Accession No.) |
| _____________________________________________________________________________ |
| **Eutopic-to-ectopic** |
|  |
| Pooled |
| ANAPC11 Anaphase promoting complex subunit 11 -3.9 |
| (NM_001002244) |
|  |
| APOE Apolipoprotein E -3.1 |
| (NM_000041) |
|  |
| ARHGAP26 Rho GTPase activating protein 26 3.2 |
| (NM_015071) |
|  |
| BAMBI BMP and activin membrane-bound -3.1 |
| (NM_012342) inhibitor homolog (Xenopus laevis) |
|  |
| BCAM Basal cell adhesion molecule -4.5 |
| (NM_005581) (Lutheran blood group) |
|  |
| BCHE Butyrylcholinesterase 3.7 |
| (NM_000055) |
|  |
| C1orf172 Chromosome 1 open reading frame 172 3.7 |
| (NM_152365) |
|  |
| C2orf88 Chromosome 2 open reading frame 88 3.4 |
| (NM_001042519) |
|  |
| CBLN1 Cerebellin 1 precursor 3.1 |
| (NM_004352) |
|  |
| CCT6A Chaperonin containing TCP1, subunit 6A (zeta 1) -3.4 |
| (NM_001762) |
|  |
| CHEK1 CHK1 checkpoint homolog (S. pombe) 3.9 |
| (NM_001274) |
|  |
| CHP Calcium binding protein P22 3.2 |
| (NM_007236) |
|  |
| COBL Cordon-bleu homolog (mouse) 3.5 |
| (NM_015198) |
|  |
| COLEC11 Collectin sub-family member 11 -3.7 |
| (NM_199235) |
|  |
| COX6B1 Cytochrome c oxidase subunit VIb -3.1 |
| (NM_001863) polypeptide 1 (ubiquitous) |
|  |
| CSNK1G1 Casein kinase 1, gamma 1 4.2 |
| (NM_022048) |
|  |
| DBX2 Developing brain homeobox 2 5.8 |
| (NM_001004329) |
|  |
| DDAH2 Dimethylarginine dimethylaminohydrolase 2 -3.2 |
| (NM_013974) |
|  |
| DDX19A DEAD (Asp-Glu-Ala-As) box polypeptide 19A 4.0 |
| (NM_018332) |
|  |
| EEF1G Eukaryotic translation elongation factor 1 gamma -5.0 |
| (NM_001404) |
|  |
| EIF3K Eukaryotic translation initiation factor 3, subunit K -3.6 |
| (NM_013234) |
|  |
| ERBB3 V-erb-b2 erythroblastic leukemia viral 3.4 |
| (NM_001982) oncogene homolog 3 (avian) |
|  |
| FAM131B Family with sequence similarity 131, member B 3.1 |
| (NM_014690) |
|  |
| FBXO4 F-box protein 4 3.1 |
| (NM_012176) |
|  |
| FLJ32255 Hypothetical protein LOC643977 3.3 |
| (AK056817) |
|  |
| FTL Ferritin, light polypeptide -3.7 |
| (NM_000146) |
|  |
| GALNT4 UDP-N-acetyl-alpha-D-galactosamine:polypeptide 3.1 |
| (NM_003774) N-acetylgalactosaminyltransferase 4 (GalNAc-T4) |
|  |
| GNPTAB N-acetylglucosamine-1-phosphate transferase, 3.1 |
| (NM_024312) alpha and beta subunits |
|  |
| HCG_2011852 Hypothetical protein LOC643677 3.3 |
| (NM_001146197) |
|  |
| HKDC1 Hexokinase domain containing 1 3.7 |
| (NM_025130) |
|  |
| HLA-C Major histocompatibility complex, class I, C -4.7 |
| (NM_002117) |
|  |
| HMGN2 High-mobility group nucleosomal binding domain 2 -4.8 |
| (NM_005517) |
|  |
| HOMER2 Homer homolog 2 (Drosophila) 3.8 |
| (NM_199330) |
|  |
| ITGB1 Integrin, beta 1 (fibronectin receptor, beta -3.8 |
| (NM_002211) polypeptide, antigen CD29 includes MDF2, |
| MSK12) |
|  |
| KLF2 Kruppel-like factor 2 (lung) -4.6 |
| (NM_016270) |
|  |
| LAMC2 Laminin, gamma 2 4.3 |
| (NM_018891) |
|  |
| LOC221710 Hypothetical protein LOC221710 3.1 |
| (NM_001135575) |
|  |
| LOC283711 Hypothetical protein LOC283711 3.7 |
| (XR_040656) |
|  |
| LOC643037 Similar to hCG1730248 3.2 |
| (XM_926406) |
|  |
| LTA4H Leukotriene A4 hydrolase -3.3 |
| (NM_000895) |
|  |
| MAGEE1 Melanoma antigen family E, 1 3.3 |
| (NM_020932) |
|  |
| MKI67 Antigen identified by monoclonal antibody 3.4 |
| (NM_002417) Ki-67 |
|  |
| MPPED2 Metallophosphoesterase domain containing 2 4.1 |
| (NM_001584) |
|  |
| MSX2 Msh homeobox 2 3.6 |
| (NM_002449) |
|  |
| MVP Major vault protein -3.6 |
| (NM_017458) |
|  |
| NCRNA00116Non-protein coding RNA 116 5.1 |
| (NR_027063) |
|  |
| NIN Ninein (GSK3B interacting protein) 3.3 |
| (NM_016350) |
|  |
| NME3 Non-metastatic cells 3, protein expressed in -3.7 |
| (NM_002513) |
|  |
| NRCAM Neuronal cell adhesion molecule 4.5 |
| (NM_001037132) |
|  |
| NT5DC3 5'-nucleotidase domain containing 3 3.6 |
| (NM_001031701) |
|  |
| OAZ1 Ornithine decarboxylase antizyme 1 -6.6 |
| (NM_004152) |
|  |
| PARG Poly (ADP-ribose) glycohydrolase 3.8 |
| (NM_003631) |
|  |
| PFKFB3 6-phosphofructo-2-kinase/fructose-2, -5.0 |
| (NM_004566) 6-biphosphatase 3 |
|  |
| POLA2 Polymerase (DNA directed), alpha 2 3.2 |
| (NM_002689) (70kD subunit) |
|  |
| POLR3GL Polymerase (RNA) III (DNA directed) -3.3 |
| (NM_032305) polypeptide G (32kD)-like |
|  |
| PPBP Pro-platelet basic protein (chemokine 3.1 |
| (NM_002704) (C-X-C motif) ligand 7) |
|  |
| RAB39B RAB39B, member RAS oncogene family 3.7 |
| (NM_171998) |
|  |
| RABL3 RAB, member of RAS oncogene family-like 3 3.1 |
| (NM_173825) |
|  |
| RAPH1 Ras association (RalGDS/AF-6) and 3.9 |
| (NM_213589) pleckstrin homology domains 1 |
|  |
| RARRES2 Retinoic acid receptor responder -3.1 |
| (NM_002889) (tazarotene induced) 2 |
|  |
| RASD1 RAS, dexamethasone-induced 1 3.9 |
| (NM_016084) |
|  |
| RASEF RAS and EF-hand domain containing 6.7 |
| (NM_152573) |
|  |
| RPLP1 Ribosomal protein, large, P1 -7.1 |
| (NM_001003) |
|  |
| RRP1 Ribosomal RNA processing 1 homolog 3.5 |
| (NM_003683) (S. cerevisiae) |
|  |
| RGL4 Ral guanine nucleotide dissociation 3.5 |
| (NM_153615) stimulator-like4 |
|  |
| RPL7A Ribosomal protein L7a -3.1 |
| (NM_000972) |
|  |
| RPS10 Ribosomal protein S10 -3.5 |
| (NM_001014) |
|  |
| RPL10 Ribosomal protein L10 -4.5 |
| (NM_006013) |
|  |
| RPL19 Ribosomal protein L19 -6.9 |
| (NM_000981) |
|  |
| RPL21 Ribosomal protein L21 -3.6 |
| (BC104478) |
|  |
| RPL21 Ribosomal protein L21 -4.7 |
| (NM_000982) |
|  |
| RPL23A Ribosomal protein L23a -6.0 |
| (NM_000984) |
|  |
| RPL29 Ribosomal protein L29 -3.7 |
| (NM_000992) |
|  |
| SAMD1 Sterile alpha motif domain containing 1 3.7 |
| (NM_138352) |
|  |
| SCOC Short coiled-coil protein 5.1 |
| (NM_032547) |
|  |
| SEPP1 Selenoprotein P, plasma, 1 -3.3 |
| (NM_005410) |
|  |
| SLC2A4RG SLC2A4 regulator -3.3 |
| (NM_020062) |
|  |
| SLC4A7 Solute carrier family 4, sodium bicarbonate 3.1 |
| (NM_003615) cotransporter, member 7 |
|  |
| SLC12A7 Solute carrier family 12 -3.3 |
| (NM_006598) (potassium/chloride transporters), member 7 |
|  |
| SLC12A9 Solute carrier family 12 (potassium/ -3.4 |
| (NM_020246) chloride transporters), member 9 |
|  |
| STAR Steroidogenic acute regulatory protein -4.1 |
| (NM_000349) |
|  |
| TACSTD2 Tumor-associated calcium signal transducer 2 3.1 |
| (NM_002353) |
|  |
| TMEM158 Transmembrane protein 158 (gene/pseudogene) -3.2 |
| (NM_015444) |
|  |
| TMPO Thymopoietin 3.1 |
| (NM_003276) |
|  |
| TNFAIP2 Tumor necrosis factor, alpha-induced protein 2 -3.3 |
| (NM_006291) |
|  |
| UGT8 UDP glycosyltransferase 8 3.1 |
| (NM_003360) |
|  |
| VPS28 Vacuolar protein sorting 28 homolog -3.8 |
| (NM_183057) (S. cerevisiae) |
|  |
| VTCN1 V-set domain containing T cell activation 4.0 |
| (NM_024626) inhibitor1 |
|  |
| WISP2 WNT1 inducible signaling pathway protein 2 -3.9 |
| (NM_003881) |
|  |
| WNT16 Wingless-type MMTV integration site 6.7 |
| (NM_057168) family, member 16 |
|  |
| ZNF713 Zinc finger protein 713 -3.8 |
| (NM_182633) |
| _____________________________________________________________________________ |
|  |
| Stage 3 |
| ACTR3 ARP3 actin-related protein 3 homolog (yeast) -3.9 |
| (NM_005721) |
|  |
| ARL1 ADP-ribosylation factor-like 1 -3.1 |
| (NM_001177) |
|  |
| ASH1L Ash1 (absent, small, or homeotic)-like -4.4 |
| (NM_018489) (Drosophila) |
|  |
| C10orf10 Chromosome 10 open reading frame 10 -3.3 |
| (NM_007021) |
|  |
| C10orf32 Chromosome 10 open reading frame 32 -4.1 |
| (NM_144591) |
| C10orf54 Chromosome 10 open reading frame 54 -7.7 |
| (NM_022153) |
|  |
| C12orf48 Chromosome 12 open reading frame 48 3.7 |
| (NM_017915) |
|  |
| CCR1 Chemokine (C-C motif) receptor 1 -3.8 |
| (NM_001295) |
|  |
| CNBP CCHC-type zinc finger, nucleic acid -4.8 |
| (NM_003418) binding protein |
|  |
| COL14A1 Collagen, type XIV, alpha 1 -5.9 |
| (NM_021110) |
|  |
| CSTA Cystatin A (stefin A) -3.9 |
| (NM_005213) |
|  |
| CX3CL1 Chemokine (C-X3-C motif) ligand 1 -6.4 |
| (NM_002996) |
|  |
| DNAJB2 DnaJ (Hsp40) homolog, subfamily B, member 2 -3.9 |
| (NM_006736) |
|  |
| EIF1AY Eukaryotic translation initiation factor 1A, -3.3 |
| (NM_004681) Y-linked |
|  |
| FAM89B Family with sequence similarity 89, member B -3.1 |
| (NM_152832) |
|  |
| FAM90A7 Family with sequence similarity 90, member A7 -3.6 |
| (NM_001136572) |
|  |
| GATA6 GATA binding protein 6 -4.3 |
| (NM_005257) |
|  |
| IGLL1 Immunoglobulin lambda locus -3.1 |
| (BC012159) |
|  |
| JAK3 Janus kinase 3 -3.4 |
| (NM_000215) |
|  |
| JMJD7 Jumonji domain containing 7 -3.4 |
| (NM_001114632) |
|  |
| KIAA1958 KIAA1958 -4.4 |
| (ENST00000337530) |
|  |
| KLF2 Kruppel-like factor 2 (lung) -8.6 |
| (NM_016270) |
| LAMC2 Laminin, gamma 2 4.3 |
| (NM_018891) |
|  |
| LIMCH1 LIM and calponin homology domains 1 -3.4 |
| (NM_014988) |
|  |
| LOC440983 Hypothetical gene supported by BC066916 -3.1 |
| (BC066916) |
|  |
| MED22 Mediator complex subunit 22 -3.2 |
| (NM_133640) |
|  |
| MMP23B Matrix metallopeptidase 23B -5.5 |
| (NM_006983) |
|  |
| MYO1D Myosin ID -3.5 |
| (NM_015194) |
|  |
| NIT2 Nitrilase family, member 2 -3.4 |
| (NM_020202) |
|  |
| NME3 Non-metastatic cells 3, protein expressed -6.7 |
| (NM_002513) |
|  |
| OCIAD1 OCIA domain containing 1 -4.9 |
| (NM_001079839) |
|  |
| OS9 Osteosarcoma amplified 9, endoplasmic -4.0 |
| (NM_006812) reticulum lectin |
|  |
| PAFAH1B2 Platelet-activating factor acetylhydrolase 1b, -3.1 |
| (BC001774) catalytic subunit 2 (30kDa) |
|  |
| PFDN5 Prefoldin subunit 5 -4.9 |
| (NM_002624) |
|  |
| PSMB1 Proteasome (prosome, macropain) subunit, -3.4 |
| (NM_002793) beta type, 1 |
|  |
| PVRL3 Poliovirus receptor-related 3 -4.9 |
| (NM_015480) |
|  |
| RALGDS Ral guanine nucleotide dissociation stimulator -3.6 |
| (NM_001042368) |
|  |
| RASEF RAS and EF-hand domain containing 3.7 |
| (NM_152573) |
|  |
| SLC25A35 Solute carrier family 25, member 35 4.0 |
| (NM_201520) |
|  |
| SLC45A3 Solute carrier family 45, member 3 -4.6 |
| (NM_033102) |
|  |
| STARD7 StAR-related lipid transfer (START) -3.1 |
| (NM_020151) domain containing 7 |
|  |
| TACSTD2 Tumor-associated calcium signal transducer 2 3.1 |
| (NM_002353) |
|  |
| TCEA1 Transcription elongation factor A (SII), 1 -3.3 |
| (NM_006756) |
|  |
| TCEAL5 Transcription elongation factor A (SII)-like 5 -3.8 |
| (NM_001012979) |
|  |
| TMEM127 Transmembrane protein 127 -3.1 |
| (NM_017849) |
|  |
| TNFAIP2 Tumor necrosis factor, alpha-induced protein 2 -4.3 |
| (NM_006291) |
|  |
| TNFRSF14 Tumor necrosis factor receptor superfamily, -4.8 |
| (NM_003820) member 14 (herpesvirus entry mediator) |
|  |
| TSHB Thyroid stimulating hormone, beta 3.7 |
| (NM_000549) |
|  |
| TSHZ1 Teashirt zinc finger homeobox 1 -3.2 |
| (NM_005786) |
|  |
| UBXN1 UBX domain protein 1 -5.4 |
| (NM_015853) |
|  |
| UQCR11 Ubiquinol-cytochrome c reductase, -4.1 |
| (NM_006830) complex III subunit XI |
|  |
| UTP15 UTP15, U3 small nucleolar ribonucleoprotein, -4.0 |
| (NM_032175) homolog (S. cerevisiae) |
|  |
| ZDHHC7 Zinc finger, DHHC-type containing 7 -3.5 |
| (NM_017740) |
|  |
| ZNF283 Zinc finger protein 283 -4.7 |
| (NM_181845) |
|  |
| ZNF367 Zinc finger protein 367 6.3 |
| (NM_153695) |
|  |
| _____________________________________________________________________________ |
| Stage 4 |
| ADAMTS9 ADAM metallopeptidase with thrombospondin 5.0 |
| (AF261918) type 1 motif, 9 |
|  |
| ADCY6 Adenylate cyclase 6 3.6 |
| (NM_015270) |
|  |
| AFG3L2 AFG3 ATPase family gene 3-like 2 (yeast) 3.1 |
| (NM_006796) |
|  |
| AP1AR Adaptor-related protein complex 1 4.4 |
| (NM_018569) type 1 motif, 9 |
|  |
| B3GALTL Beta 1,3-galactosyltransferase-like 3.6 |
| (NM_194318) |
|  |
| C17orf80 Chromosome 17 open reading frame 80 3.9 |
| (NM_017941) |
|  |
| C1QB Complement component 1, q -4.4 |
| (NM_000491) subcomponent, B chain |
|  |
| CENPN Centromere protein N 4.0 |
| (NM_001100624) |
|  |
| CSTF3 Cleavage stimulation factor, 3' pre-RNA, 3.5 |
| (NM_001033505) subunit 3, 77kDa |
|  |
| CTBS Chitobiase, di-N-acetyl-(CTBS), mRNA 3.6 |
| (NM_004388) |
|  |
| DOK7 Docking protein 7 3.8 |
| (NM_173660) |
|  |
| EDNRA Endothelin receptor type A 3.4 |
| (NM_001957) |
|  |
| ELP4 Elongation protein 4 homolog (S. cerevisiae) 3.4 |
| (NM_019040) |
|  |
| EXD2 Exonuclease 3'-5' domain containing 2 6.1 |
| (NM_018199) |
|  |
| GABBR1 Gamma-aminobutyric acid (GABA) B receptor, 1 4.3 |
| (NM_001470) |
|  |
| KCTD15 Potassium channel tetramerisation 3.8 |
| (NM_024076) domain containing 15 |
|  |
| LAMC2 Laminin, gamma 2 3.5 |
| (NM_018891) |
|  |
| LPAR3 Lysophosphatidic acid receptor 3 4.2 |
| (AK091731) |
|  |
| MAGT1 Magnesium transporter 1 9.2 |
| (NM_032121) |
|  |
| MINPP1 Multiple inositol polyphosphate 3.3 |
| (NM_004897) histidine phosphatase, 1 |
|  |
| NANOS1 Nanos homolog 1 (Drosophila) 4.7 |
| (NM_199461) |
|  |
| NBN Nibrin 3.2 |
| (NM_002485) |
|  |
| PDE11A Phosphodiesterase 11A 7.3 |
| (NM_016953) |
|  |
| PPARA Peroxisome proliferator-activated receptor alpha 3.2 |
| (NM_005036) |
|  |
| PPIA Peptidylprolyl isomerase A (cyclophilin A) -3.7 |
| (NM_021130) |
|  |
| RASEF RAS and EF-hand domain containing 6.9 |
| (NM_152573) |
|  |
| RMND1 Required for meiotic nuclear division 1 5.4 |
| (NM_017909) homolog (S. cerevisiae) |
|  |
| RNF115 Ring finger protein 115 4.1 |
| (NM_014455) |
|  |
| RXFP1 Relaxin/insulin-like family peptide receptor 1 3.9 |
| (AK295040) |
|  |
| SLC22A3 Solute carrier family 22 (extraneuronal 4.4 |
| (NM_021977) monoamine transporter), member 3 |
|  |
| SLC35E1 Solute carrier family 35, member E1 3.9 |
| (NM_024881) |
|  |
| STIP1 Stress-induced-phosphoprotein 1 6.3 |
| (NM_006819) |
|  |
| TACSTD2 Tumor-associated calcium signal transducer 2 3.1 |
| (NM_002353) |
|  |
| TP53I11 Tumor protein p53 inducible protein 11 3.1 |
| (NM_001076787) |
|  |
| TRAF3IP2 TRAF3 interacting protein 2 3.6 |
| (NM_147686) |
|  |
| TSGA14 Testis specific, 14 4.2 |
| (NM_018718) |
|  |
| ZNF713 Zinc finger protein 713 -7.3 |
| (NM_182633) |
| ___________________________________________________________________________ |
|  |
| Proliferative phase |
|  |
| ADRBK2 Adrenergic, beta, receptor kinase 2 3.8 |
| (NM_005160) |
|  |
| AKAP14 A kinase (PRKA) anchor protein 14 4.3 |
| (NM_178813) |
|  |
| AMPH Amphiphysin 7.1 |
| (NM_001635) |
|  |
| ATF3 Activating transcription factor 3 6.3 |
| (NM_001040619) |
|  |
| B3GALTL Beta 1,3-galactosyltransferase-like 3.6 |
| (NM_194318) |
|  |
| BAG5 BCL2-associated athanogene 5 4.2 |
| (NM_001015049) |
|  |
| BCR Breakpoint cluster region 3.1 |
| (NM_004327) |
|  |
| BOD1L Biorientation of chromosomes 8.0 |
| (NM_148894) in cell division 1-like |
|  |
| BTBD11 BTB (POZ) domain containing 11 3.7 |
| (NM_001018072) |
|  |
| C1orf198 Chromosome 1 open reading -3.7 |
| (NM_032800) frame 198 |
|  |
| C1orf217 Chromosome 1 open reading 5.1 |
| (BC000988) frame 217 |
|  |
| C2orf49 Chromosome 2 open reading 4.3 |
| (NM_024093) frame 49 |
|  |
| C5orf13 Chromosome 5 open reading 4.0 |
| (NM_004772) frame 13 |
|  |
| C9orf102 Chromosome 9 open reading 5.0 |
| (NM_1010895)frame 102 |
|  |
| C9orf140 Chromosome 9 open reading 3.8 |
| (NM_178448) frame 140 |
|  |
| C10orf32 Chromosome 10 open reading -4.1 |
| (NM_144591) frame 32 |
|  |
| C10orf79 Chromosome 10 open reading 3.1 |
| (NM_025145) frame 79 |
|  |
| C12orf48 Chromosome 12 open reading 3.3 |
| (NM_017915) frame 48 |
|  |
| C12orf69 Chromosome 12 open reading 5.0 |
| (NM_1013698)frame 69 |
|  |
| C20orf54 Chromosome 20 open reading 3.1 |
| (NM_033409) frame 54 |
|  |
| CBLN1 Cerebellin 1 precursor 4.8 |
| (NM_004352) |
|  |
| CCBE1 Collagen and calcium binding EGF 5.0 |
| (NM_133459) domains 1 |
|  |
| CDCA3 Cell division cycle associated 3 3.9 |
| (NM_031299) |
|  |
| CDK18 Cyclin-dependent kinase 18 6.1 |
| (NM_212503) |
|  |
| CDKN3 Cyclin-dependent kinase inhibitor 3 3.1 |
| (NM_005192) |
|  |
| CENPV Centromere protein V 3.9 |
| (NM_181716) |
|  |
| CHD3 Chromodomain helicase DNA binding 4.0 |
| (NM_1005273)protein 3 |
|  |
| CREB5 CAMP responsive element binding 3.3 |
| (NM_182898) protein 5 |
|  |
| CSTF3 Cleavage stimulation factor, 3' 5.6 |
| (NM_1033505) pre-RNA, subunit 3, 77kDa |
|  |
| CTCF CCCTC-binding factor 4.8 |
| (NM_006565) (zinc finger protein) |
|  |
| CX3CL1 Chemokine (C-X3-C motif) ligand 1 -6.4 |
| (NM_002996) |
|  |
| CXADR Coxsackie virus and adenovirus 3.7 |
| (NM_001338) receptor |
|  |
| DIAPH3 Diaphanous homolog 3 (Drosophila) 7.8 |
| (NM_030932) |
|  |
| DSCR6 Down syndrome critical region gene 6 4.2 |
| (NM_018962) |
|  |
| DUSP8 Dual specificity phosphatase 8 4.1 |
| (NM_004420) |
|  |
| DYNC1I1 Dynein, cytoplasmic 1, intermediate 3.8 |
| (NM_004411) chain 1 |
|  |
| EGFL6 EGF-like-domain, multiple 6 3.3 |
| (NM_015507) |
|  |
| EGR3 Early growth response 3 3.7 |
| (NM_004430) |
|  |
| EHF Ets homologous factor 3.4 |
| (NM_012153) |
|  |
| ERBB3 v-erb-b2 erythroblastic leukemia viral 3.2 |
| (NM_001982) oncogene homolog 3 (avian) |
|  |
| EXD2 Exonuclease 3'-5' domain containing 2 5.1 |
| (NM_018199) |
|  |
| FAM89B Family with sequence similarity 89, -3.1 |
| (NM_152832) member B |
|  |
| FDXR Ferredoxin reductase -3.2 |
| (NM_004110) |
|  |
| FGFBP1 Fibroblast growth factor binding 5.7 |
| (NM_005130) protein 1 |
|  |
| FGFR1 Fibroblast growth factor receptor 1 7.9 |
| (AK024388) |
|  |
| FMN2 Formin 2 3.9 |
| (NM_020066) |
|  |
| FUT9 Fucosyltransferase 9 (alpha (1,3) 8.2 |
| (NM_006581) fucosyltransferase) |
|  |
| GALP Galanin-like peptide 4.9 |
| (NM_033106) |
|  |
| GAN Gigaxonin 5.9 |
| (NM_022041) |
|  |
| GFRA2 GDNF family receptor alpha 2 5.1 |
| (NM_001495) |
|  |
| GPD2 Glycerol-3-phosphate dehydrogenase 3.1 |
| (NM_000408) 2 (mitochondrial) |
|  |
| HELLS Helicase, lymphoid-specific 3.1 |
| (NM_018063) |
|  |
| HNRNPU Heterogeneous nuclear 5.6 |
| (NM_004501) ribonucleoprotein U (scaffold attachment |
| factor A) |
|  |
| INADL InaD-like (Drosophila) 5.8 |
| (NM_176877) |
|  |
| IRF1 Interferon regulatory factor 1 3.3 |
| (NM_002198) |
|  |
| JMJD7 Jumonji domain containing 7 -3.4 |
| (NM_001114632) |
|  |
| KIF18A Kinesin family member 18A 3.6 |
| (NM_031217) |
|  |
| KIF26B Kinesin family member 26B 3.1 |
| (NM_018012) |
|  |
| KMO Kynurenine 3-monooxygenase 3.4 |
| (NM_003679) (kynurenine 3-hydroxylase) |
|  |
| KREMEN2 Kringle containing transmembrane 3.4 |
| (NM_172229) protein 2 |
|  |
| KRT34 Keratin 34 6.2 |
| (NM_021013) |
|  |
| LAMB1 Laminin, beta 1 -3.2 |
| (NM_002291) |
|  |
| LAMC2 Laminin, gamma 2 3.4 |
| (NM_018891) |
|  |
| LMNB2 Lamin B2 3.4 |
| (NM_032737) |
|  |
| LOC283711 Hypothetical protein LOC283711 7.7 |
| (XR_040656) |
|  |
| LOC284454 Hypothetical protein LOC284454 7.9 |
| (BX640708) |
|  |
| LOC440288 Similar to FLJ16518 protein 5.1 |
| (BC014373) |
|  |
| LOC729817 Similar to mCG50656 5.1 |
| (XR_078892) |
|  |
| LOC90246 Hypothetical protein LOC90246 3.3 |
| (NR_026954) |
|  |
| LRRC37A2 Leucine rich repeat containing 37, 4.6 |
| (NM_1006607) member A2 |
|  |
| LYRM4 LYR motif containing 4 4.5 |
| (NM_020408) |
|  |
| MANF Mesencephalic astrocyte-derived -4.8 |
| (NM_006010) neurotrophic factor |
|  |
| MAP4K4 Mitogen-activated protein kinase 6.4 |
| (NM_145686) kinase kinase kinase 4 |
|  |
| MATN4 Matrilin 4 3.1 |
| (NM_003833) |
|  |
| MCCC2 Methylcrotonoyl-Coenzyme A 3.1 |
| (NM_022132) carboxylase 2 (beta) |
|  |
| MLLT4 Myeloid/lymphoid or mixed-lineage 10.8 |
| (BC014505) leukemia (trithorax homolog, Drosophila); |
| translocated to, 4 |
|  |
| MYL12B Myosin, light chain 12B, regulatory -5.6 |
| (NM_033546) |
|  |
| NRTN Neurturin 5.4 |
| (NM_004558) |
|  |
| NSA2 NSA2 ribosome biogenesis homolog -3.1 |
| (NM_014886) (S. cerevisiae) |
|  |
| ORM1 Orosomucoid 1 3.3 |
| (NM_000607) |
|  |
| OVOL2 Ovo-like 2 (Drosophila) 3.5 |
| (NM_021220) |
|  |
| PAK7 P21 protein (Cdc42/Rac)-activated 5.1 |
| (NM_020341) kinase 7 |
|  |
| PDE7A Phosphodiesterase 7A 3.7 |
| (NM_002603) |
|  |
| PHF23 PHD finger protein 23 5.8 |
| (NM_024297) |
|  |
| PHYHD1 Phytanoyl-CoA dioxygenase domain 3.9 |
| (NM_174933) containing 1 |
|  |
| PLA1A Phospholipase A1 member A 4.6 |
| (NM_015900) |
|  |
| POU5F1 POU class 5 homeobox 1 3.6 |
| (NM_002701) |
|  |
| PPFIA1 Protein tyrosine phosphatase, 3.6 |
| (NM_003626) receptor type, f polypeptide |
| (PTPRF), interacting protein |
| (liprin), alpha 1 |
|  |
| PPP2R3A Protein phosphatase 2 (formerly 2A), 3.9 |
| (NM_002718) regulatory subunit B'', alpha |
|  |
| PROX1 Prospero homeobox 1 7.2 |
| (NM_002763) |
|  |
| PRRT2 Proline-rich transmembrane protein 2 -3.2 |
| (NM_145239) |
|  |
| RAD51 RAD51 homolog (RecA homolog, 3.1 |
| (NM_002875) E. coli) (S. cerevisiae) |
|  |
| RASGRF1 Ras protein-specific guanine 6.7 |
| (NM_002891) nucleotide-releasing factor 1 |
|  |
| RNF115 Ring finger protein 115 4.1 |
| (NM_014455) |
|  |
| RPL7A Ribosomal protein L7a -4.7 |
| (NM_000972) |
|  |
| RPS27 Ribosomal protein S27 -6.6 |
| (NM_001030) |
|  |
| RPS28 Ribosomal protein S28 -5.3 |
| (NM_001031) |
|  |
| SCNM1 Sodium channel modifier 1 3.8 |
| (NM_024041) |
|  |
| SDC3 Syndecan 3 3.1 |
| (NM_014654) |
|  |
| SDK2 Sidekick homolog 2 (chicken) 5.7 |
| (NM_001144952) |
|  |
| SDR42E1 Short chain dehydrogenase/reductase 8.2 |
| (NM_145168) family 42E, member 1 |
|  |
| SERINC2 Serine incorporator 2 3.6 |
| (NM_178865) |
|  |
| SF3B5 Splicing factor 3b, subunit 5, 10kDa -4.6 |
| (NM_031287) |
|  |
| SHMT1 Serine hydroxymethyltransferase 1 3.4 |
| (NM_004169) (soluble) |
|  |
| SIK1 Salt-inducible kinase 1 4.1 |
| (NM_173354) |
|  |
| SIN3A SIN3 homolog A, transcription 5.6 |
| (NM_001145358) regulator (yeast) |
|  |
| SKA1 Spindle and kinetochore associated 5.5 |
| (NM_1039535) complex subunit 1 |
|  |
| SLC35A3 Solute carrier family 35 (UDP-N- 3.9 |
| (BC005136) acetylglucosamine (UDP-GlcNAc) |
| transporter), member A3 |
|  |
| SLFN5 Schlafen family member 5 3.3 |
| (NM_144975) |
|  |
| SMARCC2 SWI/SNF related, matrix associated, -3.7 |
| (NM_139067) actin dependent regulator of |
| chromatin, subfamily c, member 2 |
|  |
| SPAG11B Sperm associated antigen 11B 7.4 |
| (NM_016512) |
|  |
| SPRED1 Sprouty-related, EVH1 domain 3.1 |
| (NM_152594) containing 1 |
|  |
| SRCIN1 SRC kinase signaling inhibitor 1 5.7 |
| (NM_025248) |
|  |
| TACC3 Transforming, acidic coiled-coil 4.3 |
| (NM_006342) containing protein 3 |
|  |
| TMEM2 Transmembrane protein 2 6.1 |
| (NM_013390) |
|  |
| TMEM115 Transmembrane protein 115 5.1 |
| (NM_007024) |
|  |
| TNFRSF19 Tumor necrosis factor receptor 4.5 |
| (NM_148957) superfamily, member 19 |
|  |
| TPM1 Tropomyosin 1 (alpha) -3.1 |
| (NM_001018004) |
|  |
| TPM3 Tropomyosin 3 3.4 |
| (NM_001043352) |
|  |
| TRAF3IP2 TRAF3 interacting protein 2 11.2 |
| (AF136407) |
|  |
| TRIM11 Tripartite motif-containing 11 3.6 |
| (AK074866) |
|  |
| TTR Transthyretin 5.1 |
| (NM_000371) |
|  |
| TUBB2B Tubulin, beta 2B 4.1 |
| (NM_178012) |
|  |
| U2AF1 U2 small nuclear RNA auxiliary 4.1 |
| (AL832665) factor 1 |
|  |
| UQCR11 Ubiquinol-cytochrome c reductase, -4.1 |
| (NM_006830) complex III subunit XI |
|  |
| VWC2 Von Willebrand factor C domain 3.6 |
| (NM_198570) containing 2 |
|  |
| WHSC1 Wolf-Hirschhorn syndrome 4.7 |
| (NM_007331) candidate 1 |
|  |
| XAGE3 X antigen family, member 3 5.5 |
| (NM_130776) |
|  |
| ZDHHC7 Zinc finger, DHHC-type containing 7 -3.3 |
| (NM_017740) |
|  |
| ZFYVE28 Zinc finger, FYVE domain containing 28 3.6 |
| (NM_020972) |
|  |
| ZNF207 Zinc finger protein 207 3.2 |
| (AL834501) |
|  |
| ZNF706 Zinc finger protein 706 -3.1 |
| (NM_001042510) |
| _____________________________________________________________________________ |
|  |
| Secretory |
|  |
| AEBP1 AE binding protein 1 -4.5 |
| (NM_001129) |
|  |
| AGL Amylo-1, 6-glucosidase, 9.4 |
| (NM_000028) 4-alpha-glucanotransferase |
|  |
| ALDH1A1 Aldehyde dehydrogenase 1 -4.6 |
| (NM_000689) family, member A1 |
|  |
| ARHGAP17 Rho GTPase activating protein 17 -3.2 |
| (NM_001006634) |
|  |
| ARHGEF4 Rho guanine nucleotide -3.5 |
| (NM_032995) exchange factor (GEF) 4 |
|  |
| ARID3A AT rich interactive domain -6.7 |
| (NM_005224) 3A (BRIGHT-like) |
|  |
| ARL1 ADP-ribosylation factor-like 1 -3.4 |
| (NM_001177) |
|  |
| ATF4 Activating transcription factor 4 (tax-responsive -3.1 |
| (NM_001675) enhancer element B67) |
|  |
| BAHD1 Bromo adjacent homology -4.4 |
| (NM_014952) domain containing 1 |
|  |
| BEST4 Bestrophin 4 -3.2 |
| (NM_153274) |
|  |
| BTF3 Basic transcription factor 3 -3.8 |
| (NM_001037637) |
|  |
| C1QA Complement component 1, -3.4 |
| (NM_015991) q subcomponent, A chain |
|  |
| C1QTNF5 C1q and tumor necrosis factor related protein 5 -3.5 |
| (NM_015645) |
|  |
| C1orf172 Chromosome 1 open reading frame 172 7.4 |
| (NM_152365) |
|  |
| C1orf198 Chromosome 1 open reading frame 198 -3.7 |
| (NM_032800) |
|  |
| C6orf47 Chromosome 6 open reading frame 47 -3.3 |
| (NM_021184) |
|  |
| C7orf30 Chromosome 7 open reading frame 30 -4.5 |
| (NM_138446) |
|  |
| C9orf25 Chromosome 9 open reading frame 25 -3.1 |
| (NM_147202) |
|  |
| C9orf167 Chromosome 9 open reading frame 167 -3.1 |
| (NM_017723) |
|  |
| C10orf54 Chromosome 10 open reading frame 54 -6.4 |
| (NM_022153) |
|  |
| C11orf88 Chromosome 11 open reading frame 88 6.3 |
| (NM_207430) |
|  |
| C12orf48 Chromosome 12 open reading frame 48 3.3 |
| (NM_017915) |
|  |
| C16orf45 Chromosome 16 open reading frame 45 -4.0 |
| (NM_033201) |
|  |
| C20orf141 Chromosome 20 open reading frame 141 -5.9 |
| (NM_080739) |
|  |
| CCR1 Chemokine (C-C motif) receptor 1 -3.2 |
| (NM_001295) |
|  |
| CLDN11 Claudin 11 -3.3 |
| (NM_005602) |
|  |
| COL14A1 Collagen, type XIV, alpha 1 -4.4 |
| (NM_021110) |
|  |
| COPZ1 Coatomer protein complex, subunit zeta 1 -5.4 |
| (NM_016057) |
|  |
| CPZ Carboxypeptidase Z -4.7 |
| (NM_001014448) |
|  |
| CSDE1 Cold shock domain containing E1, -7.1 |
| (NM_1007553) RNA-binding |
|  |
| CTHRC1 Collagen triple helix repeat containing 1 -4.0 |
| (NM_138455) |
|  |
| D4S234E DNA segment on chromosome 4 (unique) 234 -5.8 |
| (NM_014392) expressed sequence |
|  |
| DHRS3 Dehydrogenase/reductase (SDR family) -3.6 |
| (NM_004753) member 3 |
|  |
| DHX58 DEXH (Asp-Glu-X-His) box polypeptide 58 -6.5 |
| (NM_024119) |
|  |
| DNAH12 Dynein, axonemal, heavy chain 12 3.3 |
| (NM_178504) |
|  |
| DNAJB2 DnaJ (Hsp40) homolog, subfamily B, member 2 -4.4 |
| (NM_006736) |
|  |
| DPYSL2 Dihydropyrimidinase- like 2 -3.4 |
| (NM_001386) |
|  |
| DRG2 Developmentally regulated GTP binding protein 2 -3.1 |
| (NM_001388) |
|  |
| DUSP26 Dual specificity phosphatase 26 (putative) -4.1 |
| (NM_024025) |
|  |
| EFHA1 EF-hand domain family, member A1 -4.0 |
| (NM_152726) |
|  |
| EGR3 Early growth response 3 -3.4 |
| (NM_004430) |
|  |
| EIF1AY Eukaryotic translation -3.2 |
| (NM_004681) initiation factor 1A, Y-linked |
|  |
| EIF2C1 Eukaryotic translation initiation factor 2C, 1 -3.2 |
| (NM_012199) |
|  |
| EIF4EBP1 Eukaryotic translation initiation factor 4E -3.5 |
| (NM_004095) binding |
|  |
| ERBB3 v-erb-b2 erythroblastic leukemia viral 3.7 (NM_001982) oncogene homolog 3 (avian) |
|  |
| FAM108A1 Family with sequence similarity 108, member A1 -3.4 |
| (NM_031213) |
|  |
| FNDC3B Fibronectin type III domain containing 3B -4.0 |
| (NM_022763) |
|  |
| FOLR2 Folate receptor 2 (fetal) -9.7 |
| (NM_000803) |
|  |
| GADD45B Growth arrest and DNA- damage-inducible, beta -3.1 |
| (NM_015675) |
|  |
| GANAB Glucosidase, alpha; neutral AB -3.9 |
| (NM_198335) |
|  |
| GATA6 GATA binding protein 6 -6.5 |
| (NM_005257) |
|  |
| GGT5 Gamma-glutamyltransferase 5 -3.3 |
| (NM_004121) |
|  |
| GUK1 Guanylate kinase 1 -3.4 |
| (NM_000858) |
|  |
| H2AFJ H2A histone family, member J -3.6 |
| (NM_177925) |
|  |
| HIST1H2BF Histone cluster 1, H2bf -3.5 |
| (NM_003522) |
|  |
| KIAA0467 KIAA0467 -3.6 |
| (NM_015284) |
|  |
| KIAA0895L KIAA0895-like -4.2 |
| (NM_001040715) |
|  |
| LAMC2 Laminin, gamma 2 5.7 |
| (NM_018891) |
|  |
| LAS1L LAS1-like (S. cerevisiae) -4.4 |
| (NM_031206) |
|  |
| LGTN Ligatin 7.3 |
| (NM_006893) |
|  |
| LHFP Lipoma HMGIC fusion partner -6.6 |
| (NM_005780) |
|  |
| LOC643037 Similar to hCG1730248 6.8 |
| (XM_926406) |
|  |
| LPPR4 Lipid phosphate phosphatase-related 3.6 |
| (NM_014839) protein type 4 |
|  |
| LRP3 Low density lipoprotein receptor-related protein 3 -5.4 |
| (NM_002333) |
|  |
| LTA4H Leukotriene A4 hydrolase -6.9 |
| (NM_000895) |
|  |
| LY86 Lymphocyte antigen 86 -3.1 |
| (NM_004271) |
|  |
| LZTS2 Leucine zipper, putative tumor suppressor 2 -6.2 |
| (NM_032429) |
|  |
| MATN4 Matrilin 4 5.1 |
| (NM_003833) |
|  |
| ME3 Malic enzyme 3, NADP(+)-dependent, -3.8 |
| (NM_1014811) mitochondrial |
|  |
| MEA1 Male-enhanced antigen 1 -3.1 |
| (NM_014623) |
|  |
| MGAT1 Mannosyl (alpha-1,3-)-glycoprotein beta-1,2-N- -3.5 |
| (NM_002406) acetylglucosaminyltransferase |
|  |
| MIER2 Mesoderm induction early response 1, family -3.9 |
| (NM_017550) member 2 |
|  |
| MME Membrane metallo- endopeptidase 3.4 |
| (NM_007289) |
|  |
| MMP23B Matrix metallopeptidase 23B -7.4 |
| (NM_006983) |
|  |
| MMRN2 Multimerin 2 -3.9 |
| (NM_024756) |
|  |
| MRPS34 Mitochondrial ribosomal protein S34 -5.1 |
| (NM_023936) |
|  |
| MS4A7 Membrane-spanning 4- domains, subfamily A, -3.1 |
| (NM_021201) member 7 |
|  |
| MT1G Metallothionein 1G -4.2 |
| (NM_005950) |
|  |
| MT1H Metallothionein 1H -5.1 |
| (NM_005951) |
|  |
| MT1X Metallothionein 1X -3.3 |
| (NM_005952) |
|  |
| MTSS1L Metastasis suppressor 1-like 6.2 |
| (NM_138383) |
|  |
| MVP Major vault protein -5.6 |
| (NM_017458) |
|  |
| MYL9 Myosin, light chain 9, regulatory -4.1 |
| (NM_181526) |
|  |
| MYO1C Myosin IC -10.8 |
| (NM_033375) |
|  |
| NBL1 Neuroblastoma, suppression of tumorigenicity 1 -3.1 |
| (NM_182744) |
|  |
| NBPF10 Neuroblastoma breakpoint family, member 10 -5.8 |
| (NM_1039703) |
|  |
| NDUFAF2 NADH dehydrogenase (ubiquinone) 1 alpha -4.0 |
| (NM_174889) subcomplex, assembly factor 2 |
|  |
| NME3 Non-metastatic cells 3 protein -8.3 |
| (NM_002513) |
|  |
| NUDT4 Nudix (nucleoside diphosphate linked moiety X)- -4.6 |
| (NM_199040) type motif 4 |
|  |
| OGFR Opioid growth factor receptor -5.6 |
| (NM_007346) |
|  |
| OSBP Oxysterol binding protein -3.4 |
| (NM_002556) |
|  |
| PDCD10 Programmed cell death 10 -3.1 |
| (NM_007217) |
|  |
| PEX19 Peroxisomal biogenesis factor 19 5.3 |
| (NM_002857) |
|  |
| PEX26 Peroxisomal biogenesis factor 26 -4.5 |
| (AK000065) |
|  |
| PGR Progesterone receptor 7.5 |
| (NM_000926) |
|  |
| PHF1 PHD finger protein 1 -3.3 |
| (NM_024165) |
|  |
| PLK2 Polo-like kinase 2 (Drosophila) -3.2 |
| (NM_006622) |
|  |
| POLDIP3 Polymerase (DNA-directed), delta interacting -4.0 |
| (NM_032311) protein 3 |
|  |
| POLR3H Polymerase (RNA) III polypeptide H (22.9kD) -3.1 |
| (NM_138338) |
|  |
| PORCN Porcupine homolog (Drosophila) -5.3 |
| (NM_203473) |
|  |
| PPP1CC Protein phosphatase 1, catalytic subunit, -5.5 |
| (NM_002710) gamma isozyme |
|  |
| PROS1 Protein S (alpha) -4.5 |
| (NM_000313) |
|  |
| PSAP Prosaposin -6.1 |
| (NM_001042465) |
|  |
| PVRL3 Poliovirus receptor-related 3 -4.2 |
| (NM_015480) |
|  |
| RAB24 RAB24, member RAS oncogene family -5.1 |
| (NM_1031677) |
|  |
| RAC2 Ras-related C3 botulinum toxin substrate 2 -3.3 |
| (NM_002872) (rho family,small GTP binding protein Rac2) |
|  |
| RALGDS Ral guanine nucleotide dissociation stimulator -4.2 |
| (NM_1042368) |
|  |
| RASD1 RAS, dexamethasone-induced 1 6.4 |
| (NM_016084) |
| RGS16 Regulator of G-protein signaling 16 -3.1 |
| (NM_002928) |
|  |
| RLTPR RGD motif, leucine rich repeats, tropomodulin -3.9 |
| (AK090421) domain and proline-rich containing |
|  |
| ROBO3 Roundabout, axon guidance receptor, homolog 3 -5.4 |
| (NM_022370) (Drosophila) |
|  |
| SCARA3 Scavenger receptor class A, member 3 -4.1 |
| (NM_016240) |
|  |
| SH3BP5 SH3-domain binding protein 5 (BTK-associated) -6.6 |
| (NM_004844) |
|  |
| SHC3 SHC (Src homology 2 domain containing) 3.8 |
| (NM_016848) transforming protein 3 |
|  |
| SIGLEC11 Sialic acid binding Ig-like lectin 11 -4.1 |
| (NM_052884) |
|  |
| SLC26A11 Solute carrier family 26, member 11 -7.0 |
| (NM_173626) |
|  |
| SLC27A5 Solute carrier family 27 (fatty acid transporter), -3.6 |
| (NM_012254) member 5 |
|  |
| SLC48A1 Solute carrier family 48 (heme transporter), -3.1 |
| (AK000496) member 1 |
|  |
| SNRNP200 Small nuclear ribonucleoprotein 200kDa (U5) -5.5 |
| (NM_014014) |
|  |
| SOBP Sine oculis binding protein homolog (Drosophila) -3.1 |
| (NM_018013) |
|  |
| SORL1 Sortilin-related receptor, L (DLR class) A -3.3 |
| (NM_003105) repeats-containing |
|  |
| SPRR1A Small proline-rich protein 1A -4.6 |
| (NM_005987) |
|  |
| SQLE Squalene epoxidase 5.3 |
| (NM_003129) |
|  |
| ST13 Suppression of tumorigenicity 13 -4.7 |
| (NM_003932) (colon carcinoma) (Hsp70 interacting protein) |
|  |
| STK25 Serine/threonine kinase 25 (STE20 homolog, -4.4 |
| (NM_006374) yeast) |
| TCEA1 Transcription elongation factor A (SII), 1 -3.8 |
| (NM_006756) |
|  |
| TCEAL2 Transcription elongation factor A (SII)-like 2 -6.3 |
| (NM_080390) |
|  |
| TCIRG1 T-cell, immune regulator 1, ATPase, -4.3 |
| (NM_006019) H+ transporting, lysosomal V0 subunit A3 |
|  |
| TEAD1 TEA domain family member1 (SV40 -3.4 |
| (NM_021961) transcriptional enhancer factor) |
|  |
| TMEM176A Transmembrane protein 176A -3.1 |
| (NM_018487) |
|  |
| TNIP1 TNFAIP3 interacting protein 1-5.6 |
| (NM_006058) |
|  |
| TNPO1 Transportin 1 3.3 |
| (NM_002270) |
|  |
| TRIP6 Thyroid hormone receptor interactor 6 -4.7 |
| (NM_003302) |
|  |
| TSEN34 TRNA splicing endonuclease 34 homolog -4.2 |
| (NM_1077446) (S. cerevisiae) |
|  |
| TSEN54 TRNA splicing endonuclease 54 homolog -3.1 |
| (NM_207346) (S. cerevisiae) |
|  |
| TSHZ1 Teashirt zinc finger homeobox 1 -4.3 |
| (NM_005786) |
|  |
| TXNDC11 Thioredoxin domain containing 11 -4.4 |
| (NM_015914) |
|  |
| UBXN1 UBX domain protein 1 -5.1 |
| (NM_015853) |
|  |
| UQCR11 Ubiquinol-cytochrome c reductase, -5.0 |
| (NM_006830) complex III subunit XI |
|  |
| VPS28 Vacuolar protein sorting 28 homolog -6.6 |
| (NM_183057) (S. cerevisiae) |
|  |
| VTCN1 V-set domain containing T cell activation 8.2 |
| (NM_024626) inhibitor 1 |
|  |
| ZDHHC7 Zinc finger, DHHC-type containing 7 -4.0 |
| (NM_017740) |
|  |
| ZNF219 Zinc finger protein 219 -4.1 |
| (NM_016423) |
| _____________________________________________________________________________ |
|  |
| **Clinical stages 3-to-4** |
|  |
| Eutopic |
| A2M Alpha-2-macroglobulin -6.2 |
| (NM_000014) |
|  |
| ABCC11 ATP-binding cassette, sub-family C 7.9 |
| (NM_033151) (CFTR/MRP), member 11 |
|  |
| ACBD4 Acyl-Coenzyme A binding domain containing 4 3.6 |
| (NM_024722) |
|  |
| ACTR3B ARP3 actin-related protein 3 homolog B (yeast) 6.8 |
| (NM_001040135) |
|  |
| ADAMTS3 ADAM metallopeptidase with thrombospondin 9.7 |
| (NM_014243) type 1 motif, 3 |
|  |
| ADAT2 Adenosine deaminase, tRNA-specific 2, TAD2 4.5 |
| (NM_182503) homolog (S. cerevisiae) |
|  |
| AGPS Alkylglycerone phosphate synthase 3.3 |
| (NM_003659) |
|  |
| ALAS1 Aminolevulinate, delta-, synthase 1 -5.9 |
| (NM_000688) |
|  |
| ALDH1L2 Aldehyde dehydrogenase 1 family, member L2 14.2 |
| (NM_001034173) |
|  |
| ALG1L Asparagine-linked glycosylation 1-like 3.7 |
| (NM_001015050) |
|  |
| ALMS1P Alstrom syndrome 1 pseudogene 7.6 |
| (NR_003683) |
|  |
| ANXA7 Annexin A7 -3.1 |
| (NM_004034) |
|  |
| AP1B1 Adaptor-related protein complex 1, beta 1 subunit 12.9 |
| (NM_001127) |
|  |
| AP2B1 Adaptor-related protein complex 2, beta 1 subunit 3.8 |
| (NM_001030006) |
|  |
| AP3D1 Adaptor-related protein complex 3, delta 1 subunit 3.9 |
| (NM_003938) |
|  |
| APOBEC3F Apolipoprotein B mRNA editing enzyme, 4.2 |
| (NM_145298) catalytic polypeptide-like 3F |
|  |
| AQP6 Aquaporin 6, kidney specific 16.3 |
| (NM_001652) |
|  |
| ARID4A AT rich interactive domain 4A (RBP1-like) 3.9 |
| (NM_002892) |
|  |
| ARL8B ADP-ribosylation factor-like 8B -3.6 |
| (NM_018184) |
|  |
| ASB4 Ankyrin repeat and SOCS box-containing 4 5.7 |
| (NM_016116) |
|  |
| ATP2A2 ATPase, Ca++ transporting, cardiac muscle, 5.1 |
| (NM_001681) slow twitch 2 |
|  |
| BAIAP3 BAI1-associated protein 3 6.7 |
| (NM_003933) |
|  |
| BDP1 B double prime 1, subunit of RNA polymerase III 4.0 |
| (NM_018429) transcription initiation factor IIIB |
|  |
| BRD3 Bromodomain containing 3 4.4 |
| (NM_007371) |
|  |
| C1QTNF3 C1q and tumor necrosis factor related protein 3 7.0 |
| (AK094895) |
|  |
| C4orf27 Chromosome 4 open reading frame 27 -3.9 |
| (NM_017867) |
|  |
| C6orf134 Chromosome 6 open reading frame 134 10.9 |
| (BC025755) |
|  |
| C11orf64 Chromosome 11 open reading frame 64 17.4 |
| (NR_026946) |
|  |
| C12orf40 Chromosome 12 open reading frame 40 9.1 |
| (NM_001031748) |
|  |
| C14orf4 Chromosome 14 open reading frame 4 6.2 |
| (NM_024496) |
|  |
| C20orf12 Chromosome 20 open reading frame 12 4.7 |
| (NM_001099407) |
|  |
| C20orf72 Chromosome 20 open reading frame 72 3.7 |
| (NM_052865) |
|  |
| C21orf96 Chromosome 21 open reading frame 96 11.8 |
| (NR_026812) |
|  |
| CALCOCO2 Calcium binding and coiled-coil domain 2 4.5 |
| (NM_005831) |
|  |
| CBX3 Chromobox homolog 3 (HP1 gamma 4.2 |
| (AL568696) homolog, Drosophila) |
|  |
| CC2D2A Coiled-coil and C2 domain containing 2A 4.5 |
| (NM_001080522) homolog, Drosophila) |
|  |
| CCL15 Chemokine (C-C motif) ligand 15 6.5 |
| (NM_032965) |
|  |
| CCL19 Chemokine (C-C motif) ligand 19 5.2 |
| (NM_006274) |
|  |
| CCNF Cyclin F 9.6 |
| (NM_001761) |
|  |
| CCNT2 Cyclin T2 4.0 |
| (NM_058241) |
|  |
| CD46 CD46 molecule, complement regulatory protein 13.3 |
| (NM_002389) |
|  |
| CDC2L2 Cell division cycle 2-like 2 (PITSLRE proteins) 8.5 |
| (AB209095) |
|  |
| CDC34 Cell division cycle 34 homolog (S. cerevisiae) -3.9 |
| (NM_004359) |
|  |
| CHIA Chitinase, acidic 3.4 |
| (NM_021797) |
|  |
| CHRM4 Cholinergic receptor, muscarinic 4 18.6 |
| (NM_000741) |
|  |
| CLEC14A C-type lectin domain family 14, member A 6.1 |
| (NM_175060) |
|  |
| CLTA Clathrin, light chain (Lca) -3.2 |
| (NM_007096) |
|  |
| CNBP CCHC-type zinc finger, nucleic acid -4.2 |
| (NM_003418) binding protein |
| COQ4 Coenzyme Q4 homolog (S. cerevisiae) 5.1 |
| (NM_016035) |
|  |
| CSRNP1 Cysteine-serine-rich nuclear protein 1 5.5 |
| (NM_033027) |
|  |
| CX3CL1 Chemokine (C-X3-C motif) ligand 1 -3.1 |
| (NM_002996) |
|  |
| CXCL3 Chemokine (C-X-C motif) ligand 3 3.3 |
| (NM_002090) |
|  |
| CXorf38 Chromosome X open reading frame 38 5.0 |
| (NM_144970) |
|  |
| CYBRD1 Cytochrome b reductase 1 -5.9 |
| (NM_024843) |
|  |
| CYYR1 Cysteine/tyrosine-rich 1 3.6 |
| (NM_052954) |
|  |
| DAGLB Diacylglycerol lipase, beta 3.1 |
| (NM_139179) |
|  |
| DAPK2 Death-associated protein kinase 2 6.2 |
| (NM_014326) |
|  |
| DCAF5 DDB1 and CUL4 associated factor 5 3.8 |
| (NM_003861) |
|  |
| DDX18 DEAD (Asp-Glu-Ala-Asp) box polypeptide 18 5.4 |
| (NM_006773) |
|  |
| DEPDC1 DEP domain containing 1 4.2 |
| (NM_017779) |
|  |
| DLX2 Distal-less homeobox 2 10.1 |
| (NM_004405) |
|  |
| DNAH7 Dynein, axonemal, heavy chain 7 7.5 |
| (NM_018897) |
|  |
| DNAJC10 DnaJ (Hsp40) homolog, subfamily C, -4.0 |
| (NM_018981) member 10 |
|  |
| DUSP16 Dual specificity phosphatase 16 4.9 |
| (NM_030640) |
|  |
| DYDC2 DPY30 domain containing 2 5.5 |
| (NM_032372) |
|  |
| DYNLL1 Dynein, light chain, LC8-type 1 -5.1 |
| (NM_001037494) |
|  |
| DYRK3 Dual-specificity tyrosine-(Y)-phosphorylation 4.8 |
| (NM_001004023) regulated kinase 3 |
|  |
| E2F7 E2F transcription factor 7 3.2 |
| (NM_203394) |
|  |
| EAF1 ELL associated factor 1 6.9 |
| (NM_033083) |
|  |
| EDN2 Endothelin 2 3.6 |
| (NM_001956) |
|  |
| EFCAB6 EF-hand calcium binding domain 6 7.9 |
| (NM_022785) |
|  |
| EIF1 Eukaryotic translation initiation factor 1 -5.8 |
| (NM_005801) |
|  |
| EIF4A2 Eukaryotic translation initiation factor 4A, 9.0 |
| (BC039344) isoform 2 |
|  |
| ELP2 Elongation protein 2 homolog (S. cerevisiae) -3.8 |
| (NM_018255) |
|  |
| EPB41L1 Erythrocyte membrane protein band 4.1-like 1 -3.1 |
| (NM_012156) |
|  |
| ERC1 ELKS/RAB6-interacting/CAST family member 1 6.8 |
| (NM_178040) |
|  |
| ESCO1 Establishment of cohesion 1 homolog 1 -3.1 |
| (NM_052911) (S. cerevisiae) |
|  |
| ESRRG Estrogen-related receptor gamma 9.4 |
| (NM_206594) |
|  |
| EVC2 Ellis van Creveld syndrome 2 7.2 |
| (NM_147127) |
|  |
| EXOC5 Exocyst complex component 5 6.9 |
| (NM_006544) |
|  |
| FAM47E Family with sequence similarity 47, member E 3.1 |
| (NM_001136570) |
|  |
| FAM63A Family with sequence similarity 63, member A 3.8 |
| (NM_001040217) |
|  |
| FAM71D Family with sequence similarity 71, member D 12.8 |
| (NM_173526) |
|  |
| FAM93B Family with sequence similarity 93, member B 17.9 |
| (BC030685) |
|  |
| FAM119A Family with sequence similarity 119, member A -3.4 |
| (NM_001127395) |
|  |
| FAM123B Family with sequence similarity 123B 5.0 |
| (NM_152424) |
|  |
| FAM154B Family with sequence similarity 154, member B 6.0 |
| (NM_001008226) |
|  |
| FAR1 Fatty acyl CoA reductase 1 3.2 |
| (NM_032228) |
|  |
| FBXO9 F-box protein 9 6.0 |
| (NM_033480) |
|  |
| FBXO21 F-box protein 21 -3.9 |
| (NM_033624) |
|  |
| FLOT1 Flotillin 1 9.2 |
| (NM_005803) |
|  |
| FOXJ1 Forkhead box J1 6.2 |
| (NM_001454) |
|  |
| FOXJ3 Forkhead box J3 -3.1 |
| (NM_014947) |
|  |
| FTMT Ferritin mitochondrial 8.5 |
| (NM_177478) |
|  |
| FUT5 Fucosyltransferase 5 (alpha (1,3) 7.3 |
| (NM_002034) fucosyltransferase) |
|  |
| GCOM1 GRINL1A complex locus 10.2 |
| (NM_001018090) |
|  |
| GIGYF2 GRB10 interacting GYF protein 2 7.3 |
| (NM_015575) |
|  |
| GJC3 Gap junction protein, gamma 3, 30.2kDa 9.1 |
| (NM_181538) |
|  |
| GK5 Glycerol kinase 5 (putative) 5.2 |
| (NM_001039547) |
|  |
| GLT8D3 Glycosyltransferase 8 domain containing 3 4.5 |
| (NM_173601) |
|  |
| GNAQ Guanine nucleotide binding protein (G protein), 7.8 |
| (NM_002072) q polypeptide |
|  |
| GOT1 Glutamic-oxaloacetic transaminase 1, soluble 3.9 |
| (NM_002079) (aspartate aminotransferase 1) |
|  |
| GOT2 Glutamic-oxaloacetic transaminase 2, 7.1 |
| (NM_002080) mitochondrial (aspartate aminotransferase 2) |
|  |
| GPR4 G protein-coupled receptor 4 4.9 |
| (NM_005282) |
|  |
| GRIN2A Glutamate receptor, ionotropic, N-methyl 15.4 |
| (NM_000833) D-aspartate 2A |
|  |
| HDHD1A Haloacid dehalogenase-like hydrolase 6.1 |
| (NM_012080) domain containing 1A |
|  |
| HGSNAT Heparan-alpha-glucosaminide N-acetyltransferase 3.3 |
| (NM_152419) |
|  |
| HMGA2 High mobility group AT-hook 2 6.1 |
| (NM_003483) |
|  |
| HNRNPA1L2 Heterogeneous nuclear ribonucleoprotein -4.1 |
| (NM_001011724)A1-like 2 |
|  |
| HOXD8 Homeobox D8 4.2 |
| (NM_019558) |
|  |
| HPN Hepsin 5.9 |
| (NM_182983) |
|  |
| HPSE Heparanase 7.2 |
| (AF155510) |
|  |
| HSDL1 Hydroxysteroid dehydrogenase like 1 4.3 |
| (NM_031463) |
|  |
| IL16 Interleukin 16 6.1 |
| (NM_004513) |
|  |
| IMPACT Impact homolog (mouse) 9.7 |
| (NM_018439) |
|  |
| INCENP Inner centromere protein antigens 135/155kDa 4.2 |
| (NM_001040694) |
|  |
| ITGA5 Integrin, alpha 5 (fibronectin receptor, 4.3 |
| (NM_002205) alpha polypeptide) |
|  |
| JMJD7 Jumonji domain containing 7 -3.8 |
| (NM_001114632) |
|  |
| JRK Jerky homolog (mouse) 4.8 |
| (NM_003724) |
|  |
| KCNK12 Potassium channel, subfamily K, member 12 15.5 |
| (NM_022055) |
|  |
| KPNA4 Karyopherin alpha 4 (importin alpha 3) 7.8 |
| (NM_002268) |
|  |
| KRT31 Keratin 31 11.6 |
| (NM_002277) |
|  |
| KRT222 Keratin 222 6.0 |
| (NM_152349) |
|  |
| LAT2 Linker for activation of T cells family, member 2 4.4 |
| (NM_032464) |
|  |
| LCE1F Late cornified envelope 1F 5.4 |
| (NM_178354) |
|  |
| LOC100128343Hypothetical protein LOC100128343 3.9 |
| (AF289611) |
|  |
| LOC100287911Similar to hCG 2024922 4.7 |
| (XM_002342123) |
|  |
| LOC100289399 Similar to phospholipase A2, group X 3.1 |
| (XM_002344113) |
|  |
| LOC145694 Hypothetical protein LOC145694 4.3 |
| (AK056793) |
|  |
| LOC157740 Hypothetical protein C8orf9 13.4 |
| (AJ291676) |
|  |
| LOC285033 Hypothetical protein LOC285033 7.3 |
| (AK057419) |
|  |
| LOC285550 Hypothetical protein LOC285550 9.4 |
| (NM_001145191) |
|  |
| LOC338620 Hypothetical protein LOC338620 6.1 |
| (BC043009) |
|  |
| LOC392352 Similar to TRIMCyp 10.4 |
| (XM_002342942) |
|  |
| LOC440292 Similar to COMM domain containing 4 3.3 |
| (XM_496078) |
|  |
| LOC729046 Similar to ribosomal protein L17 -4.9 |
| (XR_015710) |
|  |
| LOC729082 Hypothetical protein LOC729082 3.7 |
| (NR_026757) |
|  |
| LRFN1 Leucine rich repeat and fibronectin type III 3.9 |
| (NM_020862) domain containing 1 |
|  |
| LRRC16B Leucine rich repeat containing 16B 7.8 |
| (NM_138360) |
|  |
| LTBP2 Latent transforming growth factor beta 7.1 |
| (NM_000428) binding protein 2 |
|  |
| MAGEA10 Melanoma antigen family A, 10 9.5 |
| (NM_001011543) |
|  |
| MAGEA4 Melanoma antigen family A, 4 15.5 |
| (NM_002362) |
|  |
| MAGI1 Membrane associated guanylate kinase, WW 7.1 |
| (NM_004742) and PDZ domain containing 1 |
|  |
| MAN1A2 Mannosidase, alpha, class 1A, member 2 5.7 |
| AK098129) |
|  |
| MAP7D3 MAP7 domain containing 3 8.4 |
| (NM_024597) |
|  |
| MARVELD3 MARVEL domain containing 3 7.5 |
| (NM_001017967) |
|  |
| MED1 Mediator complex subunit 1 5.8 |
| (NM_004774) |
|  |
| MED22 Mediator complex subunit 22 -4.8 |
| (NM_133640) |
|  |
| MEG3 Maternally expressed 3 (non-protein coding) 4.1 |
| (NR_002766) |
|  |
| MEGF8 Multiple EGF-like-domains 8 4.7 |
| (NM_001410) |
|  |
| MEN1 Multiple endocrine neoplasia I 3.2 |
| (NM_130803) |
|  |
| METT5D1 Methyltransferase 5 domain containing 1 17.8 |
| (NM_152636) |
|  |
| MLLT1 Myeloid/lymphoid or mixed-lineage leukemia 5.6 |
| (NM_005934) (trithorax homolog, Drosophila); translocated to, 1 |
|  |
| MRPL3 Mitochondrial ribosomal protein L3 -5.2 |
| (NM_007208) |
|  |
| MTP18 Mitochondrial protein 18 kDa 4.6 |
| (NM_016498) |
|  |
| MYLIP Myosin regulatory light chain interacting protein 3.1 |
| (NM_013262) |
|  |
| N4BP2L1 NEDD4 binding protein 2-like 1 -3.9 |
| (NM_052818) |
|  |
| NAPRT1 Nicotinate phosphoribosyltransferase -4.1 |
| (NM_145201) domain containing 1 |
|  |
| NEDD8 Neural precursor cell expressed, developmentally -4.3 |
| (NM_006156) down-regulated 8 |
|  |
| NEIL3 Nei endonuclease VIII-like 3 (E. coli) 5.7 |
| (NM_018248) |
|  |
| NETO2 Neuropilin (NRP) and tolloid (TLL)-like 2 4.5 |
| (NM_018092) |
|  |
| NFIB Nuclear factor I/B 6.9 |
| (NM_005596) |
|  |
| NOB1 NIN1/RPN12 binding protein 1 5.0 |
| (NM_014062) homolog (S. cerevisiae) |
|  |
| NOS1AP Nitric oxide synthase 1 (neuronal) adaptor protein 12.9 |
| (NM_014697) |
|  |
| NPAS3 Neuronal PAS domain protein 3 5.0 |
| (NM_022123) |
|  |
| NPPB Natriuretic peptide precursor B 7.3 |
| (NM_002521) |
|  |
| NRF1 Nuclear respiratory factor 1 9.3 |
| (NM_005011) |
|  |
| NRM Nurim (nuclear envelope membrane protein) 6.7 |
| (NM_007243) |
|  |
| NT5C1B 5'-nucleotidase, cytosolic IB 16.5 |
| (NM_001002006) |
|  |
| NUDT11 Nudix (nucleoside diphosphate linked 4.0 |
| (NM_018159) moiety X)-type motif 11 |
|  |
| NUDT15 Nudix (nucleoside diphosphate linked 5.9 |
| (NM_018283) moiety X)-type motif 15 |
|  |
| NUPR1 Nuclear protein, transcriptional regulator, 1 -5.4 |
| (NM_001042483) |
|  |
| ODF2L Outer dense fiber of sperm tails 2-like 3.8 |
| (NM_020729) |
|  |
| OR5A1 Olfactory receptor, family 5, subfamily A, 10.3 |
| (NM_001004728) member 1 |
|  |
| ORC5L Origin recognition complex, subunit 5-like (yeast) 5.2 |
| (NM_002553) |
|  |
| OVCA2 Ovarian tumor suppressor candidate 2 -5.9 |
| (NM_080822) |
|  |
| PABPN1 Poly(A) binding protein, nuclear 1 -3.4 |
| (NM_004643) |
|  |
| PADI3 Peptidyl arginine deiminase, type III 19.3 |
| (NM_016233) |
|  |
| PAPPA Pregnancy-associated plasma protein A, 10.0 |
| (NM_002581) pappalysin 1 |
|  |
| PARP10 Poly (ADP-ribose) polymerase family, -5.6 |
| (NM_032789) member 10 |
|  |
| PDS5A PDS5, regulator of cohesion maintenance, 4.7 |
| (NM_001100400) homolog A (S. cerevisiae) |
|  |
| PECAM1 Platelet/endothelial cell adhesion molecule 5.4 |
| (NM_000442) |
|  |
| PFDN5 Prefoldin subunit 5 -4.7 |
| (NM_002624) |
|  |
| PKNOX2 PBX/knotted 1 homeobox 2 5.2 |
| (NM_022062) |
|  |
| PLEKHA4 Pleckstrin homology domain containing, family A -3.4 |
| (NM_020904) (phosphoinositide binding specific) member 4 |
|  |
| PLGLB1 Plasminogen-like B1 5.9 |
| (NM_001032392) |
|  |
| PLK1S1 Polo-like kinase 1 substrate 1 4.1 |
| (BC039296) |
|  |
| PNMA3 Paraneoplastic antigen MA3 -3.2 |
| (NM_013364) |
|  |
| PPFIA1 Protein tyrosine phosphatase, receptor type, f 4.9 |
| (NM_177423) polypeptide (PTPRF), interacting protein (liprin), |
| alpha 1 |
|  |
| PPIAL4A Peptidylprolyl isomerase A (cyclophilin A)- 5.2 |
| (NM_178230) like 4A |
|  |
| PPM1H Protein phosphatase 1H (PP2C domain containing) 6.4 |
| (NM_020700) |
|  |
| PRR5 Proline rich 5 (renal) -5.6 |
| (NM_015366) |
|  |
| PRR5- ARHGAP8 PRR5-ARHGAP8 readthrough 4.8 |
| (NM_181334) |
|  |
| PRTN3 Proteinase 3 10.3 |
| (NM_002777) |
|  |
| PSME3 Proteasome (prosome, macropain) activator 5.9 |
| (NM_176863) subunit 3 (PA28 gamma; Ki) |
|  |
| PTBP1 Polypyrimidine tract binding protein 1 7.1 |
| (NM_002819) |
|  |
| PTGS2 Prostaglandin-endoperoxide synthase 2 4.2 |
| (NM_000963) (prostaglandin G/H synthase and cyclooxygenase) |
|  |
| PVT1 Pvt1 oncogene (non-protein coding) 3.8 |
| (NR_003367) |
|  |
| PYROXD1 Pyridine nucleotide-disulphide oxidoreductase 5.8 |
| (NM_024854) domain 1 |
|  |
| RAB6C RAB6C, member RAS oncogene family 6.4 |
| (AL136727) |
|  |
| RAPGEF1 Rap guanine nucleotide exchange factor (GEF) 1 13.3 |
| (NM_198679) |
|  |
| RAPH1 Ras association (RalGDS/AF-6) and pleckstrin 7.9 |
| (NM_213589) homology domains 1 |
|  |
| RASSF1 Ras association (RalGDS/AF-6) domain family 3.2 |
| (NM_170713) member 1 |
|  |
| RBMX RNA binding motif protein, X-linked 6.4 |
| (NM_002139) |
|  |
| RBMXL2 RNA binding motif protein, X-linked-like 2 13.0 |
| (NM_014469) |
|  |
| RFX5 Regulatory factor X, 5 (influences HLA 7.7 |
| (NM_000449) class II expression) |
|  |
| RHOD Ras homolog gene family, member D 7.1 |
| (NM_014578) |
|  |
| RMND1 Required for meiotic nuclear division 1 3.9 |
| (NM_017909) homolog (S. cerevisiae) |
|  |
| RNASEH2A Ribonuclease H2, subunit A 4.9 |
| (NM_006397) |
|  |
| RPAP2 RNA polymerase II associated protein 2 4.6 |
| (NM_024813) |
|  |
| RPL7 Ribosomal protein L7 -6.6 |
| (NM_000971) |
|  |
| RPL12 Ribosomal protein L12 -6.4 |
| (AK057602) |
|  |
| RPL15 Ribosomal protein L15 -3.8 |
| (NM_002948) |
|  |
| RPL21 Ribosomal protein L21 -4.4 |
| (NM_000982) |
|  |
| RPL22 Ribosomal protein L22 -9.3 |
| (NM_000983) |
|  |
| RPL29 Ribosomal protein L29 -4.8 |
| (NM_000992) |
|  |
| RPS10 Ribosomal protein S10 -4.2 |
| (NM_001014) |
|  |
| RPS14 Ribosomal protein S14 -6.3 |
| (NM_001025071) |
|  |
| RPS3A Ribosomal protein S3A -7.2 |
| (NM_001006) |
|  |
| RPUSD1 RNA pseudouridylate synthase domain 3.8 |
| (NM_058192) containing 1 |
|  |
| RYK RYK receptor-like tyrosine kinase 6.9 |
| (NM_001005861) |
|  |
| S1PR5 Sphingosine-1-phosphate receptor 5 7.3 |
| (NM_030760) |
|  |
| SCN4A Sodium channel, voltage-gated, type IV, 17.6 |
| (NM_000334) alpha subunit |
|  |
| SF3A2 Splicing factor 3a, subunit 2, 66kDa -5.6 |
| (NM_007165) |
|  |
| SHF Src homology 2 domain containing F 3.5 |
| (NM_138356) |
|  |
| SLC12A1 Solute carrier family 12 (sodium/potassium/ 9.5 |
| (NM_000338) chloride transporters), member 1 |
|  |
| SLC35A4 Solute carrier family 35, member A4 4.8 |
| (NM_080670) |
|  |
| SLC46A2 Solute carrier family 46, member 2 5.6 |
| (NM_033051) |
|  |
| SNX12 Sorting nexin 12 5.7 |
| (NM_013346) |
|  |
| SNX24 Sorting nexin 24 4.7 |
| (NM_014035) |
|  |
| SOCS5 Suppressor of cytokine signaling 5 9.5 |
| (NM_144949) |
|  |
| SOCS7 Suppressor of cytokine signaling 7 9.3 |
| (NM_014598) |
|  |
| SPATA18 Spermatogenesis associated 18 homolog (rat) 7.9 |
| (NM_145263) |
|  |
| SPTLC3 Serine palmitoyltransferase, long chain 3.2 |
| (NM_018327) base subunit 3 |
|  |
| SSX2 Synovial sarcoma, X breakpoint 2 7.0 |
| (NM_175698) |
|  |
| SSX2IP Synovial sarcoma, X breakpoint 2 5.8 |
| (NM_014021) interacting protein |
|  |
| ST3GAL2 ST3 beta-galactoside alpha-2,3-sialyltransferase 2 5.1 |
| (NM_006927) |
|  |
| STAT2 Signal transducer and activator of transcription 2, 6.4 |
| (NM_005419) 113kDa |
|  |
| SYNCRIP Synaptotagmin binding, cytoplasmic RNA 9.1 |
| (NM_006372) interacting protein |
|  |
| TACC2 Transforming, acidic coiled-coil containing 13.7 |
| (NM_206862) protein 2 |
|  |
| TALDO1 Transaldolase 1 -3.7 |
| (NM_006755) |
|  |
| TBCEL Tubulin folding cofactor E-like 5.9 |
| (NM_152715) |
|  |
| TBRG4 Transforming growth factor beta regulator 4 5.4 |
| (NM_030900) |
|  |
| TCAM1 Testicular cell adhesion molecule 1 16.9 |
| (NR_002947) homolog (mouse) |
|  |
| TCEA1 Transcription elongation factor A (SII), 1 -3.8 |
| (NM_006756) |
|  |
| TGM3 Transglutaminase 3 (E polypeptide, protein- 10.1 |
| (NM_003245) glutamine-gamma-glutamyltransferase) |
|  |
| THRA Thyroid hormone receptor, alpha (erythroblastic -4.3 |
| (NM_003250) leukemia viral (v-erb-a) oncogene homolog, avian) |
|  |
| THSD1 Thrombospondin, type I, domain containing 1 -3.1 |
| (NM_018676) |
|  |
| TIMM10 Translocase of inner mitochondrial membrane 4.5 |
| (NM_012456) 10 homolog (yeast) |
|  |
| TMEM92 Transmembrane protein 92 5.6 |
| (NM_153229) |
|  |
| TMEM99 Transmembrane protein 99 -3.5 |
| (NM_145274) |
|  |
| TMEM107 Transmembrane protein 107 4.7 |
| (NM_032354) |
|  |
| TMPRSS6 Transmembrane protease, serine 6 11.7 |
| (NM_153609) |
|  |
| TNFRSF11A Tumor necrosis factor receptor superfamily, 5.1 |
| (NM_003839) member 11a, NFKB activator |
|  |
| TP53INP2 Tumor protein p53 inducible nuclear protein 2 3.9 |
| (NM_021202) |
|  |
| TRIM14 Tripartite motif-containing 14 6.7 |
| (NM_014788) |
|  |
| TRIM26 Tripartite motif-containing 26 4.2 |
| (NM_003449) |
|  |
| TSHB Thyroid stimulating hormone, beta 4.4 |
| (NM_000549) |
|  |
| UBA52 Ubiquitin A-52 residue ribosomal protein -5.9 |
| (NM_001033930) fusion product 1 |
|  |
| UBE2G2 Ubiquitin-conjugating enzyme E2G 2 3.5 |
| (NM_182688) (UBC7 homolog, yeast) |
|  |
| UBE2Q1 Ubiquitin-conjugating enzyme E2Q family 3.6 |
| (NM_017582) member 1 |
|  |
| USP7 Ubiquitin specific peptidase 7 (herpes 4.1 |
| (NM_003470) virus-associated) |
|  |
| VNN1 Vanin 1 5.3 |
| (NM_004666) |
|  |
| VPS8 Vacuolar protein sorting 8 homolog (S. cerevisiae) 4.8 |
| (NM_001009921) |
|  |
| VPS41 Vacuolar protein sorting 41 homolog (S. cerevisiae) 6.6 |
| (BX648347) |
|  |
| WBSCR17 Williams-Beuren syndrome chromosome 5.4 |
| (NM_022479) region 17 |
|  |
| WHSC1 Wolf-Hirschhorn syndrome candidate 1 3.3 |
| (NM_133334) |
|  |
| XAB2 XPA binding protein 2 5.8 |
| (NM_020196) |
|  |
| ZBTB22 Zinc finger and BTB domain containing 22 5.2 |
| (NM_005453) |
|  |
| ZNF18 Zinc finger protein 18 6.0 |
| (NM_144680) |
|  |
| ZNF80 Zinc finger protein 80 12.7 |
| (NM_007136) |
|  |
| ZNF135 Zinc finger protein 135 9.0 |
| (AL157426) |
|  |
| ZNF257 Zinc finger protein 257 7.8 |
| (NM_033468) |
|  |
| ZNF274 Zinc finger protein 274 6.3 |
| (NM_133502) |
|  |
| ZNF280A Zinc finger protein 280A 9.6 |
| (NM_080740) |
|  |
| ZNF343 Zinc finger protein 343 4.7 |
| (NM_024325) |
|  |
| ZNF367 Zinc finger protein 367 11.9 |
| (NM_153695) |
|  |
| ZNF551 Zinc finger protein 551 3.9 |
| (NM_138347) |
|  |
| ZNF621 Zinc finger protein 621 6.0 |
| (NM_198484) |
|  |
| ZNF711 Zinc finger protein 711 5.7 |
| (NM_021998) |
|  |
| ZRANB2 Zinc finger, RAN-binding domain containing 2 4.1 |
| (NM_203350) |
|  |
| Ectopic |
| ABCC13 ATP-binding cassette, sub-family C 11.8 |
| (NR_003087) (CFTR/MRP), member 13 |
|  |
| ABHD12B Abhydrolase domain containing 12B 15.1 |
| (NM_181533) |
|  |
| ABI3BP ABI family, member 3 (NESH) binding protein 9.9 |
| (NM_015429) |
|  |
| ACBD3 Acyl-Coenzyme A binding domain containing 3 5.8 |
| (NM_022735) |
|  |
| ACO1 Aconitase 1, soluble -3.1 |
| (NM_002197) |
|  |
| ACOT4 Acyl-CoA thioesterase 4 7.1 |
| (NM_152331) |
|  |
| ACSS2 Acyl-CoA synthetase short-chain family member 2 4.0 |
| (NM_018677) |
|  |
| ADAM6 ADAM metallopeptidase domain 6 (pseudogene) 23.2 |
| (NR_002224) |
|  |
| ADAM10 ADAM metallopeptidase domain 10 6.3 |
| (NM_001110) |
|  |
| ADCY2 Adenylate cyclase 2 (brain) 11.3 |
| (NM_020546) |
|  |
| ADCY5 Adenylate cyclase 5 5.8 |
| (NM_183357) |
|  |
| ADCYAP1 Adenylate cyclase activating 10.8 |
| (NM_001117) polypeptide 1 (pituitary) |
|  |
| ADORA1 Adenosine A1 receptor 18.1 |
| (NM_000674) |
|  |
| ADPGK ADP-dependent glucokinase 4.5 |
| (NM_031284) |
|  |
| ADPRHL1 ADP-ribosylhydrolase like 1 6.6 |
| (NM_138430) |
| ADRB3 Adrenergic, beta-3-, receptor 9.9 |
| (NM_000025) |
|  |
| ADSS Adenylosuccinate synthase 7.7 |
| (NM_001126) |
|  |
| AFG3L2 AFG3 ATPase family gene3-like 2 (yeast) 7.4 |
| (NM_006796) |
|  |
| AFMID Arylformamidase 4.2 |
| (NM_001010982) |
|  |
| AGBL3 ATP/GTP binding protein-like 3 16.6 |
| (AK023045) |
|  |
| AKR1E2 Aldo-keto reductase family 1, member E2 7.0 |
| (AB040820) |
|  |
| AKT3 V-akt murine thymoma viral 3.6 |
| (NM_005465) oncogene homolog 3 (protein kinase |
| B,gamma) |
|  |
| ALAS1 Aminolevulinate, delta-, synthase 1 13.7 |
| (NM_000688) |
|  |
| ALDH1L2 Aldehyde dehydrogenase 1 family, member L2 14.8 |
| (NM_001034173) |
|  |
| ALG10B Asparagine-linked glycosylation 10, alpha-1,2- 9.8 |
| (NM_001013620) glucosyltransferase homolog B (yeast) |
|  |
| ALMS1P Alstrom syndrome 1 pseudogene 7.9 |
| (NR_003683) |
|  |
| ALS2CR4 Amyotrophic lateral sclerosis2 (juvenile) 3.4 |
| (NM_001044385) chromosome region, candidate 4 |
|  |
| AMIGO1 Adhesion molecule with Ig-like domain 1 8.9 |
| (AB032989) |
|  |
| ANGPT4 Angiopoietin 4 20.3 |
| (NM_015985) |
|  |
| ANGPTL5 Angiopoietin-like 5 7.8 |
| (NM_178127) |
|  |
| ANKRD12 Ankyrin repeat domain 12 6.7 |
| (NM_015208) |
|  |
| ANTXR1 Anthrax toxin receptor 1 6.5 |
| (NM_053034) |
|  |
| AP1B1 Adaptor-related protein complex 1, 6.8 |
| (NM_001127) beta 1 subunit |
|  |
| AP4B1 Adaptor-related protein complex 4, beta 1 subunit 3.4 |
| (NM_006594) |
|  |
| APCS Amyloid P component, serum 10.9 |
| (NM_001639) |
|  |
| APIP APAF1 interacting protein 7.2 |
| (NM_015957) |
|  |
| APOBEC2 Apolipoprotein B mRNA editing 12.5 |
| (NM_006789) enzyme, catalytic polypeptide-like 2 |
|  |
| APOL2 Apolipoprotein L, 2 10.0 |
| (NM_145637) |
|  |
| APOOL Apolipoprotein O-like 10.8 |
| (NM_198450) |
|  |
| AQP9 Aquaporin 9 6.6 |
| (NM_020980) |
|  |
| ARAF V-raf murine sarcoma 3611 6.1 |
| (NM_001654) viral oncogene homolog |
|  |
| ARHGEF15 Rho guanine nucleotide 5.4 |
| (NM_173728) exchange factor (GEF) 15 |
|  |
| ARHGEF2 Rho/Rac guanine nucleotide 4.2 |
| (NM_004723) exchange factor (GEF) 2 |
|  |
| ARHGEF9 Cdc42 guanine nucleotide 3.2 |
| (NM_015185) exchange factor (GEF) 9 |
|  |
| ARID1A AT rich interactive domain1A (SWI-like) 8.9 |
| (NM_006015) |
|  |
| ARL13B ADP-ribosylation factor-like 13B 6.6 |
| (NM_182896) |
|  |
| ARL5B ADP-ribosylation factor-like 5B 9.9 |
| (NM_178815) |
|  |
| ARMC8 Armadillo repeat containing 8 3.6 |
| (NM_213654) |
|  |
| ASB4 Ankyrin repeat and SOCS 17.4 |
| (AK096449) box-containing 4 |
|  |
| ASH1L Ash1 (absent, small, or homeotic)-like 7.7 |
| (NM_018489) (Drosophila) |
|  |
| ASXL3 Additional sex combs like 3 (Drosophila) 8.3 |
| (NM_030632) |
|  |
| ATE1 Arginyltransferase 1 4.5 |
| (NM_001001976) |
|  |
| ATG10 ATG10 autophagy related 10 homolog 6.5 |
| (NM_031482) (S. cerevisiae) |
|  |
| ATP11C ATPase, class VI, type 11C 4.9 |
| (NM_173694) |
|  |
| ATP2B3 ATPase, Ca++ transporting, plasma membrane 3 7.3 |
| (NM_021949) |
|  |
| ATP5G3 ATP synthase, H+ transporting, mitochondrial F0 7.3 |
| (NM_001002258) complex, subunit C3 (subunit 9) |
|  |
| ATP8A1 ATPase, aminophospholipid transporter (APLT), 7.6 |
| (NM_006095) class I, type 8A,member 1 |
|  |
| ATRNL1 Attractin-like 1 8.3 |
| (NM_207303) |
|  |
| B3GALTL Beta 1,3-galactosyltransferase-like 16.7 |
| (NM_194318) |
|  |
| B3GNT4 UDP-GlcNAc:betaGal beta- 11.1 |
| (NM_030765) 1,3-N-acetylglucosaminyltransferase 4 |
|  |
| B4GALNT3 Beta-1,4-N-acetyl-galactosaminyl 12.0 |
| (NM_173593) transferase 3 |
|  |
| BAP1 BRCA1 associated protein-1 12.6 |
| (NM_004656) (ubiquitin carboxy-terminal hydrolase) |
|  |
| BAT1 HLA-B associated transcript 1 4.5 |
| (NM_004640) |
|  |
| BAT2 HLA-B associated transcript 2 3.5 |
| (NM_080686) |
|  |
| BAT2L HLA-B associated transcript 2-like 6.7 |
| (NM_013318) |
|  |
| BCLAF1 BCL2-associated transcription 5.2 |
| (NM_014739) factor 1 |
|  |
| BEST3 Bestrophin 3 11.6 |
| (NM_032735) |
|  |
| BMPR2 Bone morphogenetic protein 7.3 |
| (NM_001204) receptor, type II (serine/threonine kinase) |
|  |
| BNC2 Basonuclin 2 8.8 |
| (NM_017637) |
|  |
| BRWD3 Bromodomain and WD repeat 7.9 |
| (NM_153252) domain containing 3 |
|  |
| C1orf26 Chromosome 1 open reading frame 26 5.7 |
| (NM_017673) |
|  |
| C1orf104 Chromosome 1 open reading frame 104 8.8 |
| (AK125510) |
|  |
| C1orf161 Chromosome 1 open reading frame 161 10.0 |
| (NM_152367) |
|  |
| C1QTNF3 C1q and tumor necrosis factor related protein 3 7.1 |
| (AK094895) |
|  |
| C2 Complement component 2 4.3 |
| (NM_000063) |
|  |
| C3orf33 Chromosome 3 open reading frame 33 4.9 |
| (NM_173657) |
|  |
| C3orf38 Chromosome 3 open reading frame 38 4.1 |
| (NM_173824) |
| C3orf42 Chromosome 3 open reading frame 42 11.9 |
| (NR_026829) |
|  |
| C3orf51 Chromosome 3 open reading frame 51 5.2 |
| (NR_024615) |
| C3orf58 Chromosome 3 open reading frame 58 5.8 |
| (NM_173552) |
|  |
| C4orf12 Chromosome 4 open reading frame 12 4.3 |
| (AK124663) |
|  |
| C4orf23 Chromosome 4 open reading frame 23 6.9 |
| (NM_152544) |
|  |
| C5orf54 Chromosome 5 open reading frame 54 3.1 |
| (NM_022090) |
|  |
| C6orf134 Chromosome 6 open reading frame 134 33.2 |
| (BC025755) |
|  |
| C7orf53 Chromosome 7 open reading 10.3 |
| (NM_182597) |
|  |
| C8orf59 Chromosome 8 open reading frame 59 6.2 |
| (BC032347) |
|  |
| C9orf66 Chromosome 9 open reading frame 66 8.0 |
| (NM_152569) |
| C10orf53 Chromosome 10 open reading frame 53 19.7 |
| (ENST00000374112) |
|  |
| C10orf72 Chromosome 10 open reading frame 72 4.5 |
| (NM_001031746) |
|  |
| C10orf76 Chromosome 10 open reading frame 76 7.2 |
| (NM_024541) |
|  |
| C11orf63 Chromosome 11 open reading frame 63 15.1 |
| (NM_199124) |
|  |
| C11orf64 Chromosome 11 open reading frame 64 20.9 |
| (NR_026946) |
|  |
| C12orf33 Chromosome 12 open reading frame 33 12.0 |
| (XR_041988) |
|  |
| C12orf73 Chromosome 12 open reading frame 73 4.0 |
| (NM_001135570) |
|  |
| C13orf1 Chromosome 13 open reading frame 1 4.2 |
| (NM_020456) |
|  |
| C16orf67 Chromosome 16 open reading frame 67 3.5 |
| (NR_024034) |
|  |
| C17orf48 Chromosome 17 open reading frame 48 3.2 |
| (NM_020233) |
|  |
| C17orf67 Chromosome 17 open reading frame 67 7.8 |
| (XM_001718395) |
|  |
| C19orf50 Chromosome 19 open reading frame 50 3.8 |
| (NM_024069) |
|  |
| C20orf12 Chromosome 20 open reading frame 12 3.8 |
| (NM_001099407) |
|  |
| C20orf144 Chromosome 20 open reading frame 144 6.0 |
| (NM_080825) |
|  |
| C21orf89 Chromosome 21 open reading frame 89 9.2 |
| (AF426268) |
|  |
| C22orf15 Chromosome 22 open reading frame 15 8.5 |
| (NM_182520) |
|  |
| CAB39 Calcium binding protein 39 5.5 |
| (NM_016289) |
|  |
| CACNA1C Calcium channel, voltage- 4.9 |
| (NM_000719) dependent, L type, alpha 1C subunit |
|  |
| CACNB2 Calcium channel, voltage- 11.9 |
| (AK128769) dependent, beta 2 subunit |
|  |
| CAD Carbamoyl-phosphate 5.1 |
| (NM_004341) synthetase 2, aspartate transcarbamylase, |
| and dihydroorotase |
|  |
| CALCOCO1 Calcium binding and coiled-coil domain 1 9.2 |
| (NM_020898) |
|  |
| CALCOCO2 Calcium binding and coiled-coil domain 2 6.1 |
| (NM_005831) |
|  |
| CAMTA2 Calmodulin binding transcription activator 2 9.3 |
| (NM_015099) |
|  |
| CAPN10 Calpain 10 8.6 |
| (NM_023083) |
|  |
| CBLL1 Cas-Br-M (murine) ecotropic 6.2 |
| (NM_024814) retroviral transforming sequence-like 1 |
|  |
| CCBE1 Collagen and calcium binding EGF domains 1 8.0 |
| (NM_133459) |
|  |
| CCDC68 Coiled-coil domain containing 68 5.2 |
| (NM_025214) |
|  |
| CCDC88C Coiled-coil domain containing 88C 5.4 |
| (NM_001080414) |
|  |
| CCDC93 Coiled-coil domain containing 93 7.7 |
| (NM_019044) |
|  |
| CCDC150 Coiled-coil domain containing 150 4.0 |
| (NM_001080539) |
|  |
| CCDC159 Coiled-coil domain containing 159 5.2 |
| (NM_001080503) |
|  |
| CCKAR Cholecystokinin A receptor 22.2 |
| (NM_000730) |
|  |
| CCNF Cyclin F 12.8 |
| (NM_001761) |
|  |
| CCNT2 Cyclin T2 5.6 |
| (NM_058241) |
|  |
| CD200R1 CD200 receptor 1 11.5 |
| (NM_138806) |
|  |
| CD274 CD274 molecule 5.0 |
| (NM_014143) |
|  |
| CDC2L5 Cell division cycle 2-like 5 14.3 |
| (NM_031267) (cholinesterase-related cell division controller) |
|  |
| CDC42EP4 CDC42 effector protein (Rho GTPase binding) 4 4.2 |
| (NM_012121) |
|  |
| CDCA4 Cell division cycle associated 4 4.0 |
| (NM_017955) |
|  |
| CDH5 Cadherin 5, type 2 (vascular endothelium) 12.6 |
| (NM_001795) |
|  |
| CDH6 Cadherin 6, type 2, K- cadherin (fetal kidney) 8.7 |
| (NM_004932) |
|  |
| CDKL1 Cyclin-dependent kinase- like 1 6.2 |
| (NM_004196) (CDC2-related kinase) |
|  |
| CDX4 Caudal type homeobox 4 13.2 |
| (NM_005193) |
|  |
| CDYL Chromodomain protein, Y-like 6.4 |
| (NM_004824) |
|  |
| CEP120 Centrosomal protein 120kDa 4.1 |
| (NM_153223) |
|  |
| CEP192 Centrosomal protein 192kDa 13.4 |
| (NM_032142) |
|  |
| CERCAM Cerebral endothelial cell adhesion molecule 10.5 |
| (NM_016174) |
|  |
| CFHR5 Complement factor H-related 5 30.6 |
| (NM_030787) |
|  |
| CIB2 Calcium and integrin binding family member 2 7.4 |
| (NM_006383) |
|  |
| CIITA Class II, major histocompatibility complex, 9.0 |
| (NM_000246) transactivator |
|  |
| CLASP2 Cytoplasmic linker associated protein 2 8.0 |
| (NM_015097) |
|  |
| CLEC3A C-type lectin domain family 3,member A 14.6 |
| (NM_005752) |
|  |
| CLIP2 CAP-GLY domain containing linker protein 2 3.2 |
| (NM_003388) |
|  |
| CLOCK Clock homolog (mouse) 9.0 |
| (BQ879883) |
|  |
| CMIP C-Maf-inducing protein 7.1 |
| (NM_198390) |
|  |
| CMKLR1 Chemokine-like receptor 1 9.8 |
| (NM_004072) |
|  |
| CNNM2 Cyclin M2 10.5 |
| (NM_199077) |
|  |
| CNOT3 CCR4-NOT transcription complex, subunit 3 7.7 |
| (NM_014516) |
| COCH Coagulation factor C homolog, 17.3 |
| (NM_004086) cochlin (Limulus polyphemus) |
|  |
| COL15A1 Collagen, type XV, alpha 1 6.9 |
| (NM_001855) |
|  |
| COX6B2 Cytochrome c oxidase subunit 7.7 |
| (NM_144613) VIb polypeptide 2 (testis) |
|  |
| CPSF2 Cleavage and polyadenylation 20.0 |
| (NM_017437) specific factor 2, 100kDa |
|  |
| CREB1 CAMP responsive element 3.9 |
| (NM_134442) binding protein 1 |
|  |
| CREBL2 CAMP responsive element 3.4 |
| (NM_001310) binding protein-like 2 |
|  |
| CROCC Ciliary rootlet coiled-coil, 11.1 |
| (NM_014675) rootletin |
|  |
| CSAG2 CSAG family, member 2 9.4 |
| (NM_001080848) |
|  |
| CSNK1G3 Casein kinase 1, gamma 3 3.9 |
| (NM_001044723) |
|  |
| CSPP1 Centrosome and spindle pole 7.8 |
| (NM_001077204) associated protein 1 |
|  |
| CSRNP1 Cysteine-serine-rich nuclear protein 1 14.4 |
| (NM_033027) |
|  |
| CTAGE1 Cutaneous T-cell lymphoma- associated antigen 1 5.0 |
| (NM_172241) |
|  |
| CTSE Cathepsin E 11.4 |
| (NM_001910) |
|  |
| CUGBP2 CUG triplet repeat, RNA binding protein 2 3.2 |
| (NM_001025076) |
|  |
| CUZD1 CUB and zona pellucida-like domains 1 11.0 |
| (NM_022034) |
|  |
| CYP3A43 Cytochrome P450, family 3, subfamily A, 11.1 |
| (NM_022820) polypeptide 43 |
|  |
| CYTSB Cytospin B 6.5 |
| BC033618) |
|  |
| DAGLB Diacylglycerol lipase, beta 6.5 |
| (NM_139179) |
|  |
| DAOA D-amino acid oxidase activator 42.6 |
| (NM_172370) |
|  |
| DCDC1 Doublecortin domain containing 1 10.8 |
| (NM_181807) |
|  |
| DCK Deoxycytidine kinase 3.9 |
| (NM_000788) |
|  |
| DCLK1 Doublecortin-like kinase 1 4.4 |
| (NM_004734) |
|  |
| DCTN5 Dynactin 5 (p25) 13.1 |
| (NM_032486) |
|  |
| DDEF1IT1 DDEF1 intronic transcript 1(non-protein coding) 8.6 |
| (AF161539) |
|  |
| DDX60 DEAD (Asp-Glu-Ala-Asp) box polypeptide 60 5.8 |
| (NM_017631) |
|  |
| DEPDC7 DEP domain containing 7 8.1 |
| (NM_139160) |
|  |
| DHRS7B Dehydrogenase/reductase (SDR family) 5.3 |
| (NM_015510) member 7B |
|  |
| DHX33 DEAH (Asp-Glu-Ala-His) box polypeptide 33 4.7 |
| (NM_020162) |
|  |
| DKFZp434L192 Hypothetical protein DKFZp434L192 17.9 |
| (NR_026929) |
|  |
| DKFZP586K1520 DKFZP586K1520 protein 8.5 |
| (AL050153) |
|  |
| DLK2 Delta-like 2 homolog (Drosophila) 6.6 |
| (NM_206539) |
|  |
| DMRT3 Doublesex and mab-3 related transcription factor 3 17.5 |
| (NM_021240) |
|  |
| DMRTB1 DMRT-like family B with proline- rich 11.0 |
| (NM_033067) C-terminal, 1 |
|  |
| DMXL2 Dmx-like 2 14.9 |
| (NM_015263) |
|  |
| DNAH7 Dynein, axonemal, heavy chain 7 14.2 |
| (NM_018897) |
|  |
| DOC2B Double C2-like domains, beta 10.5 |
| (NM_003585) |
|  |
| DSCAM Down syndrome cell adhesion 10.3 |
| (NM_001389) molecule |
|  |
| DSCR10 Down syndrome critical region gene 10 14.6 |
| (NR_027695) |
|  |
| DUSP19 Dual specificity phosphatase 19 3.1 |
| (NM_080876) |
|  |
| DYM Dymeclin 9.1 |
| (NM_017653) |
|  |
| DYNC2H1 Dynein, cytoplasmic 2, heavy chain 1 4.1 |
| (NM_001080463) |
|  |
| EAF1 ELL associated factor 1 12.8 |
| (NM_033083) |
|  |
| EBF1 Early B-cell factor 1 6.6 |
| (NM_024007) |
|  |
| EBF3 Early B-cell factor 3 11.6 |
| (NM_001005463) |
|  |
| EFCAB6 EF-hand calcium binding domain 6 16.8 |
| NM_022785) |
|  |
| EFNA5 Ephrin-A5 17.1 |
| (NM_001962) |
|  |
| EIF2C2 Eukaryotic translation initiation factor 2C, 2 3.7 |
| (NM_012154) |
|  |
| EIF5 Eukaryotic translation initiation factor 5 3.1 |
| (NM_001969) |
|  |
| EML1 Echinoderm microtubule associated 4.9 |
| (NM_001008707) protein like 1 |
|  |
| EPC1 Enhancer of polycomb homolog 1 3.1 |
| (NM_025209) (Drosophila) |
|  |
| EPHA4 EPH receptor A4 6.3 |
| (NM_004438) |
|  |
| EPHB6 EPH receptor B6 4.8 |
| (NM_004445) |
|  |
| EPM2AIP1 EPM2A (laforin) interacting protein 1 7.4 |
| (NM_014805) |
|  |
| EPS15 Epidermal growth factor receptor 4.1 |
| (NM_001981) pathway substrate 15 |
|  |
| ESRRG Estrogen-related receptor gamma 12.6 |
| (NM_206594) |
|  |
| ETNK2 Ethanolamine kinase 2 7.3 |
| (NM_018208) |
|  |
| EVC2 Ellis van Creveld syndrome 2 7.8 |
| (NM_147127) |
|  |
| EXD3 Exonuclease 3'-5' domain containing 3 3.2 |
| (NM_017820) |
|  |
| EXOSC6 Exosome component 6 8.7 |
| (NM_058219) |
|  |
| EXTL1 Exostoses (multiple)-like 1 9.6 |
| (NM_004455) |
|  |
| EYA2 Eyes absent homolog 2 (Drosophila) -3.1 |
| (NM_005244) |
|  |
| F13A1 Coagulation factor XIII, A1 polypeptide -3.1 |
| (NM_000129) |
|  |
| FABP3 Fatty acid binding protein 3, muscle and heart 9.1 |
| (BG336702) (mammary-derived growth inhibitor) |
|  |
| FAM19A3 Family with sequence similarity19 (chemokine 21.7 |
| (NM_001004440) (C-C motif)-like), member A3 |
|  |
| FAM25A Family with sequence similarity 25, member A 9.5 |
| (NM_001146157) |
|  |
| FAM26E Family with sequence similarity26, member E 11.3 |
| (NM_153711) |
|  |
| FAM45A Family with sequence similarity45, member A 3.1 |
| (NM_207009) |
|  |
| FAM70A Family with sequence similarity70, member A 7.8 |
| (NM_017938) |
|  |
| FAM93B Family with sequence similarity 93, member B 10.8 |
| (BC030685) |
|  |
| FAM120B Family with sequence similarity 120B 4.7 |
| (NM_032448) |
|  |
| FAM126B Family with sequence similarity126, member B 4.5 |
| (NM_173822) |
|  |
| FAM154B Family with sequence similarity154, member B 13.6 |
| (NM_001008226) |
|  |
| FAM193B Family with sequence similarity193, member B 8.1 |
| (NR_024019) |
|  |
| FBXL12 F-box and leucine-rich repeat protein 12 4.4 |
| (NM_017703) |
|  |
| FBXO9 F-box protein 9 10.2 |
| (NM_033480) |
|  |
| FBXO11 F-box protein 11 8.7 |
| (NM_012167) |
|  |
| FBXO45 F-box protein 45 5.2 |
| (NM_001105573) |
|  |
| FBXW10 F-box and WD repeat domain containing 10 26.0 |
| (NM_031456) |
|  |
| FCN1 Ficolin (collagen/fibrinogen domain containing) 1 5.9 |
| (NM_002003) |
|  |
| FFAR2 Free fatty acid receptor 2 17.3 |
| (NM_005306) |
|  |
| FGF7 Fibroblast growth factor 7 (keratinocyte 11.7 |
| (NM_002009) growth factor) |
|  |
| FGF11 Fibroblast growth factor 11 10.4 |
| (NM_004112) |
|  |
| FIGN Fidgetin 12.6 |
| (NM_018086) |
|  |
| FLOT1 Flotillin 1 9.3 |
| (NM_005803) |
|  |
| FLRT3 Fibronectin leucine rich 3.7 |
| (NM_198391) transmembrane protein 3 |
|  |
| FLYWCH1 FLYWCH-type zinc finger 1 7.8 |
| (NM_020912) |
|  |
| FLYWCH2 FLYWCH family member 2 4.5 |
| (NM_138439) |
|  |
| FMO3 Flavin containing monooxygenase 3 13.8 |
| (NM_001002294) |
|  |
| FOXO3 Forkhead box O3 3.6 |
| (NM_001455) |
|  |
| FOXP2 Forkhead box P2 5.6 |
| (NM_148898) |
|  |
| FUT3 Fucosyltransferase 3 (galactoside3(4)-L- 28.2 |
| (NM_000149) fucosyltransferase, Lewis blood group) |
|  |
| GAB1 GRB2-associated binding protein 1 4.3 |
| (NM_207123) |
|  |
| GABBR1 Gamma-aminobutyric acid(GABA) B receptor, 1 6.6 |
| (NM_001470) |
|  |
| GABPB2 GA binding protein transcription factor, 4.5 |
| (NM_144618) beta subunit 2 |
|  |
| GABRD Gamma-aminobutyric acid (GABA) A receptor, 19.8 |
| (NM_000815) delta |
|  |
| GJB3 Gap junction protein, beta 3, 31kDa 8.7 |
| (NM_024009) |
|  |
| GJC3 Gap junction protein, gamma 3, 30.2kDa 19.9 |
| (NM_181538) |
|  |
| GK5 Glycerol kinase 5 (putative) 5.3 |
| (NM_001039547) |
|  |
| GKAP1 G kinase anchoring protein 1 7.7 |
| (NM_025211) |
|  |
| GLA Galactosidase, alpha -3.5 |
| (NM_000169) |
|  |
| GLI4 GLI family zinc finger 4 8.3 |
| (NM_138465) |
|  |
| GM2A GM2 ganglioside activator 9.1 |
| (NM_000405) |
|  |
| GNA14 Guanine nucleotide binding protein (G protein), 5.0 |
| (NM_004297) alpha 14 |
|  |
| GNRHR2 Gonadotropin-releasing hormone(type 2) 21.7 |
| (NR_002328) receptor 2 |
|  |
| GOLGA2L1 Golgi autoantigen, golgin subfamily a, 4.3 |
| (NM_017600) 2-like 1 |
|  |
| GOLGA8A Golgi autoantigen, golgin subfamily a, 8A 8.3 |
| (NM_181077) |
|  |
| GON4L Gon-4-like (C. elegans) 7.1 |
| (NM_001037533) |
|  |
| GOT2 Glutamic-oxaloacetic transaminase 2, 6.1 |
| (NM_002080) mitochondrial (aspartate aminotransferase 2) |
|  |
| GPA33 Glycoprotein A33 (transmembrane) 14.3 |
| (NM_005814) |
|  |
| GPM6A Glycoprotein M6A 7.7 |
| (NM_201591) |
|  |
| GPR116 G protein-coupled receptor 116 4.4 |
| (NM_015234) |
|  |
| GPR65 G protein-coupled receptor 65 7.6 |
| (NM_003608) |
|  |
| GPR35 G protein-coupled receptor 35 9.1 |
| (NM_005301) |
|  |
| GPX7 Glutathione peroxidase 7 9.6 |
| (NM_015696) |
|  |
| GREB1L Growth regulation by estrogen 10.7 |
| (NM_001142966) in breast cancer-like |
|  |
| GRXCR1 Glutaredoxin, cysteine rich 1 21.7 |
| (NM_001080476) |
|  |
| GTF2H5 General transcription factor IIH, polypeptide 5 5.6 |
| (NM_207118) |
|  |
| GTSF1L Gametocyte specific factor 1-like 18.4 |
| (NM_001008901) |
|  |
| GUCY2C Guanylate cyclase 2C (heat stable enterotoxin 13.8 |
| (NM_004963) receptor) |
|  |
| GYG2 Glycogenin 2 6.8 |
| (NM_003918) |
|  |
| HAUS5 HAUS augmin-like complex, subunit 5 8.3 |
| (NM_015302) |
|  |
| HAUS6 HAUS augmin-like complex, subunit 6 15.3 |
| (NM_017645) |
|  |
| HDHD1A Haloacid dehalogenase-like 3.7 |
| (NM_012080) hydrolase domain containing 1A |
|  |
| HIAT1 Hippocampus abundant transcript 1 3.2 |
| (NM_033055) |
|  |
| HIF1AN Hypoxia inducible factor 1, alpha subunit inhibitor 11.2 |
| (NM_017902) |
|  |
| HIST1H2AK Histone cluster 1, H2ak 10.1 |
| (BC034487) |
|  |
| HIST1H3E Histone cluster 1, H3e -3.5 |
| (NM_003532) |
|  |
| HIST2H2AA4 Histone cluster 2, H2aa4 -4.9 |
| (NM_001040874) |
|  |
| HLA-DRB6 Major histocompatibility complex, class II, 20.4 |
| (NR_001298) DR beta 6 (pseudogene) |
|  |
| HNRPLL Heterogeneous nuclear ribonucleoprotein L-like 3.4 |
| (NM_138394) |
|  |
| HOXA4 Homeobox A4 5.1 |
| (NM_002141) |
|  |
| HOXD13 Homeobox D13 12.6 |
| (NM_000523) |
|  |
| HPCA Hippocalcin 13.8 |
| (NM_002143) |
|  |
| HSD3B7 Hydroxy-delta-5-steroid dehydrogenase, 3 beta- 19.9 |
| (NM_025193) and steroid delta-isomerase 7 |
|  |
| HSPB7 Heat shock 27kDa protein family, member 7 4.9 |
| (NM_014424) (cardiovascular) |
|  |
| HSPB9 Heat shock protein, alpha-crystallin-related, B9 8.1 |
| (NM_033194) |
|  |
| HTR1A 5-hydroxytryptamine (serotonin) receptor 1A 9.5 |
| (NM_000524) |
|  |
| IFT80 Intraflagellar transport 80 homolog 6.2 |
| (NM_020800) (Chlamydomonas) |
|  |
| IGSF11 Immunoglobulin superfamily, member 11 11.2 |
| (NM_152538) |
|  |
| KZF1 IKAROS family zinc finger 1(Ikaros) 9.5 |
|  |
| (NM_006060) |
|  |
| IL2RG Interleukin 2 receptor, gamma 7.6 |
| (NM_000206) (severe combined immunodeficiency) |
|  |
| IL8RB Interleukin 8 receptor, beta 22.2 |
| (NM_001557) |
|  |
| IL10RA Interleukin 10 receptor, alpha 12.9 |
| (NM_001558) |
|  |
| IL20RB Interleukin 20 receptor beta 6.3 |
| (NM_144717) |
|  |
| IL29 Interleukin 29 (interferon, lambda 1) 21.1 |
| (NM_172140) |
|  |
| INHBB Inhibin, beta B 4.4 |
| (NM_002193) |
|  |
| INPP5E Inositol polyphosphate-5- phosphatase, 72 kDa 12.7 |
| (NM_019892) |
|  |
| INTS2 Integrator complex subunit 2 5.3 |
| (NM_020748) |
|  |
| INTS5 Integrator complex subunit 5 7.9 |
| (NM_030628) |
|  |
| INTU Inturned planar cell polarity 5.0 |
| (NM_015693) effector homolog (Drosophila) |
|  |
| IP6K1 Inositol hexakisphosphate kinase 1 6.0 |
| (NM_153273) |
|  |
| IQSEC1 IQ motif and Sec7 domain 1 4.2 |
| (NM_014869) |
|  |
| IRGC Immunity-related GTPase family, cinema 6.9 |
| (NM_019612) |
|  |
| ITFG2 Integrin alpha FG-GAP repeat 5.9 |
| (NM_018463) containing 2 |
|  |
| ITGA2 Integrin, alpha 2 (CD49B, alpha 2 subunit 9.3 |
| (NM_002203) of VLA-2 receptor) |
|  |
| ITGA9 Integrin, alpha 9 3.1 |
| (NM_002207) |
|  |
| ITGAV Integrin, alpha V (vitronectin receptor, 10.8 |
| (NM_002210) alpha polypeptide, antigen CD51) |
|  |
| ITGB8 Integrin, beta 8 33.1 |
| (NM_002214) |
|  |
| ITPRIPL1 Inositol 1,4,5-triphosphate receptor interacting 10.7 |
| (NM_178495) protein-like 1 |
|  |
| IVL Involucrin 14.0 |
| (NM_005547) |
|  |
| IYD Iodotyrosine deiodinase 11.2 |
| (NM_203395) |
|  |
| JAM3 Junctional adhesion molecule 3 5.6 |
| (NM_032801) |
|  |
| KANK2 KN motif and ankyrin repeat domains 2 3.6 |
| (NM_015493) |
|  |
| KCMF1 Potassium channel modulatory factor 1 6.5 |
| (NM_020122) |
|  |
| KCNA3 Potassium voltage-gated channel, 8.0 |
| (NM_002232) shaker-related subfamily, member 3 |
|  |
| KCNH2 Potassium voltage-gated channel, 8.0 |
| (NM_000238) subfamily H (eag-related), member 2 |
|  |
| KCNK12 Potassium channel, subfamily K, member 12 9.3 |
| (NM_022055) |
|  |
| KIAA1033 KIAA1033 11.8 |
| (NM_015275) |
|  |
| KIAA1143 KIAA1143 4.0 |
| (NM_020696) |
|  |
| KIAA1244 KIAA1244 13.7 |
| (NM_020340) |
|  |
| KIAA1529 KIAA1529 4.1 |
| (NM_020893) |
|  |
| KIAA1586 KIAA1586 6.5 |
| (NM_020931) |
|  |
| KIAA1614 KIAA1614 5.5 |
| (NM_020950) |
|  |
| KIAA2026 KIAA2026 9.8 |
| (NM_001017969) |
|  |
| KIF13A Kinesin family member 13A 6.1 |
| (NM_022113) |
|  |
| KLF7 Kruppel-like factor 7 (ubiquitous) 3.9 |
| (NM_003709) |
|  |
| KLHDC3 Kelch domain containing 3 4.2 |
| (NM_057161) |
|  |
| KLHL15 Kelch-like 15 (Drosophila) 4.5 |
| (NM_030624) |
|  |
| KLHL18 Kelch-like 18 (Drosophila) 19.5 |
| (AK023765) |
|  |
| KLHL4 Kelch-like 4 (Drosophila) 8.8 |
| (NM_057162) |
|  |
| KLHL8 Kelch-like 8 (Drosophila) 3.6 |
| (NM_020803) |
|  |
| KLHL23 Kelch-like 23 (Drosophila) 4.4 |
| (NM_144711) |
|  |
| KLK2 Kallikrein-related peptidase 2 14.5 |
| (AF336106) |
|  |
| KRT222 Keratin 222 10.3 |
| (NM_152349) |
|  |
| KRTAP4-7 Keratin associated protein 4-7 24.1 |
| (NM_033061) |
|  |
| L1TD1 LINE-1 type transposase domain containing 1 10.7 |
| (NM_019079) |
|  |
| LAMC1 Laminin, gamma 1 -3.1 |
| (NM_002293) |
|  |
| LASS4 LAG1 homolog, ceramide synthase 4 3.3 |
| (NM_024552) |
|  |
| LATS1 LATS, large tumor suppressor, homolog 1 11.4 |
| (NM_004690) (Drosophila) |
|  |
| LCE1A Late cornified envelope 1A -7.7 |
| (NM_178348) |
|  |
| LGI4 Leucine-rich repeat LGI family, member 4 7.1 |
| (NM_139284) |
|  |
| LNPEP Leucyl/cystinyl aminopeptidase 9.7 |
| (AK096804) |
|  |
| LOC100129794 Similar to hCG1804255 4.1 |
| (XM_001721668) |
|  |
| LOC100132161 Similar to hCG1993567 10.1 |
| (XM_001719650) |
|  |
| LOC100132816 Similar to Putative golgin subfamily A 8.9 |
| (XM_001718703) member 6-like protein 6 |
|  |
| LOC100292648 Similar to hCG2036828 13.0 |
| (XM_002345777) |
|  |
| LOC100294049 Similar to hCG2019710 12.0 |
| (XM_002345385) |
|  |
| LOC220594 TL132 protein 3.3 |
| (NR_003554) |
|  |
| LOC392352 Similar to TRIMCyp 11.2 |
| (XM_002342942) |
|  |
| LOC642413 Similar to Putative cathepsin L- like protein 6 4.2 |
| (XR_016155) |
|  |
| LOC728800 Similar to FLJ00402 protein 10.0 |
| (XM_002343398) |
|  |
| LOH3CR2A Loss of heterozygosity, 3, 9.7 |
| (NR_024065) chromosomal region 2, gene A |
|  |
| LONP2 Lon peptidase 2, peroxisomal 11.2 |
| (NM_031490) |
|  |
| LONRF1 LON peptidase N-terminal domain and 6.5 |
| (NM_152271) ring finger 1 |
|  |
| LPL Lipoprotein lipase 5.5 |
| (NM_000237) |
|  |
| LRRC25 Leucine rich repeat containing 25 7.2 |
| (NM_145256) |
|  |
| LRRC3 Leucine rich repeat containing 3 10.9 |
| (NM_030891) |
|  |
| LRRC33 Leucine rich repeat containing 33 8.5 |
| (NM_198565) |
|  |
| LRRC38 Leucine rich repeat containing 38 9.4 |
| (XM_059074) |
|  |
| LRRC4 Leucine rich repeat containing 4 10.5 |
| (NM_022143) |
|  |
| LY6G5B Lymphocyte antigen 6 complex, locus G5B 11.9 |
| (NM_021221) |
|  |
| LYRM7 Lyrm7 homolog (mouse) 4.6 |
| (NM_181705) |
|  |
| MAGEA10 Melanoma antigen family A, 10 13.1 |
| (NM_001011543) |
|  |
| MAN2C1 Mannosidase, alpha, class 2C, member 1 17.9 |
| (BC010081) |
|  |
| MANEA Mannosidase, endo-alpha 3.1 |
| (NM_024641) |
|  |
| MAP3K7IP1 Mitogen-activated protein kinase 6.0 |
| (NM_006116) kinase kinase 7 interacting protein 1 |
|  |
| MAPK8IP3 Mitogen-activated protein kinase 8 12.7 |
| (NM_015133) interacting protein 3 |
|  |
| MAS1 MAS1 oncogene 30.9 |
| (NM_002377) |
|  |
| MBOAT2 Membrane bound O- acyltransferase 3.5 |
| (NM_138799) domain containing 2 |
|  |
| MCAT Malonyl CoA:ACP acyltransferase 4.3 |
| (NM_173467) (mitochondrial) |
|  |
| MCF2L MCF.2 cell line derived transforming 6.1 |
| (NM_024979) sequence-like |
|  |
| MCM7 Minichromosome maintenance complex -4.2 |
| (NM_182776) component 7 |
|  |
| MDH1B Malate dehydrogenase 1B, NAD (soluble) 17.4 |
| (NM_001039845) |
|  |
| MED13L Mediator complex subunit 13-like 23.5 |
| (NM_015335) |
|  |
| MED17 Mediator complex subunit 17 3.4 |
| (NM_004268) |
|  |
| MED22 Mediator complex subunit 22 7.6 |
| (NM_133640) |
|  |
| MED23 Mediator complex subunit 23 7.7 |
| (NM_015979) |
|  |
| METT5D1 Methyltransferase 5 domain containing 1 6.0 |
| (NM_152636) |
|  |
| METTL9 Methyltransferase like 9 3.7 |
| (NM_016025) |
|  |
| MGC21881 Hypothetical locus MGC21881 3.9 |
| (NR_015363) |
|  |
| MLF1 Myeloid leukemia factor 1 4.3 |
| (NM_022443) |
|  |
| MLL Myeloid/lymphoid or mixed- lineage leukemia 13.8 |
| (NM_005933) (trithorax homolog,Drosophila) |
|  |
| MLYCD Malonyl-CoA decarboxylase 5.4 |
| (NM_012213) |
|  |
| MNX1 Motor neuron and pancreas homeobox 1 11.5 |
| (NM_005515) |
|  |
| MOBKL2B MOB1, Mps One Binder kinase activator-like 2B 4.5 |
| (NM_024761) (yeast) |
|  |
| MRE11A MRE11 meiotic recombination11 homolog A 6.1 |
| (NM_005590) (S. cerevisiae) |
|  |
| MRPL35 Mitochondrial ribosomal protein L35 3.2 |
| (NM_145644) |
|  |
| MSL1 Male-specific lethal 1 homolog (Drosophila) 3.5 |
| (NM_001012241) |
|  |
| MSN Moesin 11.8 |
| (NM_002444) |
|  |
| MTMR8 Myotubularin related protein 8 8.8 |
| (NM_017677) |
|  |
| MYO15A Myosin XVA 18.2 |
| (NM_016239) |
|  |
| MYO16 Myosin XVI 12.5 |
| (NM_015011) |
|  |
| NAB2 NGFI-A binding protein 2 8.2 |
| (NM_005967) (EGR1 binding protein 2) |
|  |
| NANOS1 Nanos homolog 1 (Drosophila) 6.7 |
| (NM_199461) |
|  |
| NAT11 N-acetyltransferase 11 6.8 |
| (NM_024771) (GCN5-related, putative) |
|  |
| NDEL1 NudE nuclear distribution gene E homolog 5.1 |
| (NM_001025579) (A. nidulans)-like 1 |
|  |
| NDST2 N-deacetylase/N-sulfotransferase 5.6 |
| (NM_003635) (heparan glucosaminyl) 2 |
|  |
| NDUFA3 NADH dehydrogenase(ubiquinone) 1 -3.1 |
| (NM_004542) alpha subcomplex, 3, 9kDa |
|  |
| NECAB1 N-terminal EF-hand calcium binding protein 1 9.0 |
| (NM_022351) |
|  |
| NEK3 NIMA (never in mitosis gene a)-related kinase 3 15.8 |
| (NM_002498) |
|  |
| NFE2L3 Nuclear factor (erythroid- derived 2)-like 3 15.1 |
| (NM_004289) |
|  |
| NFIB Nuclear factor I/B 8.3 |
| (NM_005596) |
|  |
| NGFR Nerve growth factor receptor 12.2 |
| (NM_002507) (TNFR superfamily, member 16) |
|  |
| NHLRC2 NHL repeat containing 2 8.8 |
| (NM_198514) |
|  |
| NHS Nance-Horan syndrome(congenital cataracts and 5.5 |
| (NM_198270) dental anomalies) |
|  |
| NKTR Natural killer-tumor recognition sequence 5.7 |
| (NM_005385) |
|  |
| NKX3-2 NK3 homeobox 2 10.5 |
| (NM_001189) |
|  |
| NLRP10 NLR family, pyrin domain containing 10 16.3 |
| (NM_176821) |
|  |
| NMT2 N-myristoyltransferase 2 8.0 |
| (NM_004808) |
|  |
| NOB1 NIN1/RPN12 binding protein 1 homolog 6.0 |
| (NM_014062) (S. cerevisiae) |
|  |
| NOL6 Nucleolar protein family 6(RNA-associated) 13.4 |
| (NM_022917) |
|  |
| NOS1AP Nitric oxide synthase 1(neuronal) adaptor protein 13.8 |
| (NM_014697) |
|  |
| NPNT Nephronectin 4.7 |
| (NM_001033047) |
|  |
| NR2C1 Nuclear receptor subfamily 2, group C, 10.5 |
| (NM_001032287) member 1 |
|  |
| NR5A1 Nuclear receptor subfamily 5, group A, 9.2 |
| (NM_004959) member 1 |
|  |
| NRM Nurim (nuclear envelope membrane protein) 4.0 |
| (NM_007243) |
|  |
| NRP2 Neuropilin 2 8.3 |
| (NM_201266) |
|  |
| NT5C1B 5'-nucleotidase, cytosolic IB 34.0 |
| (NM_001002006) |
|  |
| NUMBL Numb homolog (Drosophila)-like 7.1 |
| (NM_004756) |
|  |
| NUP210 Nucleoporin 210kDa 4.6 |
| (NM_024923) |
|  |
| NUP50 Nucleoporin 50kDa 16.0 |
| (NM_007172) |
|  |
| OBP2A Odorant binding protein 2A 15.3 |
| (NM_014582) |
|  |
| OLA1 Obg-like ATPase 1 6.7 |
| (NM_013341) |
|  |
| OMA1 OMA1 homolog, zinc metallopeptidase 3.7 |
| (NM_145243) (S. cerevisiae) |
|  |
| OR2A7 Olfactory receptor, family 2, subfamily A, 14.3 |
| (NM_001005328) member 7 |
|  |
| OSBP Oxysterol binding protein 5.8 |
| (NM_002556) |
|  |
| OSMR Oncostatin M receptor 7.7 |
| (BC010943) |
|  |
| OTUD5 OTU domain containing 5 8.9 |
| (NM_017602) |
|  |
| OTUD7B OTU domain containing 7B 19.9 |
| (NM_020205) |
|  |
| OXNAD1 Oxidoreductase NAD-binding domain 4.2 |
| (NM_138381) containing 1 |
|  |
| OXTR Oxytocin receptor 11.9 |
| (NM_000916) |
|  |
| PACRGL PARK2 co-regulated-like 6.5 |
| (NM_145048) |
|  |
| PAK2 P21 protein (Cdc42/Rac)- activated kinase 2 3.5 |
| (NM_002577) |
|  |
| PARN Poly (A)-specific ribonuclease(deadenylation 3.9 |
| (NM_002582) nuclease) |
|  |
| PATZ1 POZ (BTB) and AT hook containing zinc finger 1 6.1 |
| (NM_014323) |
|  |
| PBX2 Pre-B-cell leukemia homeobox 2 5.1 |
| (NM_002586) |
|  |
| PCSK4 Proprotein convertase subtilisin/kexin type 4 3.9 |
| (NM_017573) |
|  |
| PCTK1 PCTAIRE protein kinase 1 5.1 |
| (NM_033018) |
|  |
| PDIA3 Protein disulfide isomerase family A, -3.6 |
| (NM_005313) member 3 |
|  |
| PDK4 Pyruvate dehydrogenase kinase, isozyme 4 6.1 |
| (NM_002612) |
|  |
| PDZRN4 PDZ domain containing ring finger 4 6.9 |
| (NM_013377) |
|  |
| PECR Peroxisomal trans-2- enoyl-CoA reductase 3.2 |
| (NM_018441) |
|  |
| PEG10 Paternally expressed 10 5.9 |
| (NM_001040152) |
|  |
| PES1 Pescadillo homolog 1, containing BRCT domain 10.2 |
| (NM_014303) (zebrafish) |
|  |
| PGAP1 Post-GPI attachment to proteins 1 10.1 |
| (NM_024989) |
|  |
| PHC1 Polyhomeotic homolog 1 (Drosophila) 4.3 |
| (NM_004426) |
|  |
| PHC3 Polyhomeotic homolog 3 (Drosophila) 7.4 |
| (NM_024947) |
|  |
| PHF8 PHD finger protein 8 3.1 |
| (NM_015107) |
|  |
| PHF12 PHD finger protein 12 12.6 |
| (NM_020889) |
|  |
| PHF17 PHD finger protein 17 5.7 |
| (NM_199320) |
|  |
| PHF20L1 PHD finger protein 20-like 1 19.3 |
| (NM_198513) |
|  |
| PHLDA1 Pleckstrin homology-like domain, family A, 7.3 |
| (NM_007350) member 1 |
|  |
| PHLPP1 PH domain and leucine rich repeat protein 11.9 |
| (NM_194449) phosphatase 1 |
|  |
| PHPT1 Phosphohistidine phosphatase 1 -4.4 |
| (NM_014172) |
|  |
| PICALM Phosphatidylinositol binding clathrin assembly 4.7 |
| (NM_007166) protein |
|  |
| PINX1 PIN2-interacting protein 1 7.5 |
| (NM_017884) |
|  |
| PIP Prolactin-induced protein 12.8 |
| (NM_002652) |
|  |
| PITPNC1 Phosphatidylinositol transfer protein, 8.2 |
| (AK094724) cytoplasmic 1 |
|  |
| PITPNM2 Phosphatidylinositol transfer protein, 7.7 |
| (NM_020845) membrane-associated 2 |
|  |
| PIWIL2 Piwi-like 2 (Drosophila) 7.6 |
| (NM_018068) |
|  |
| PKD2L2 Polycystic kidney disease 2-like 2 6.9 |
| (NM_014386) |
|  |
| PKNOX2 PBX/knotted 1 homeobox 2 8.1 |
| (NM_022062) |
|  |
| PLAG1 Pleiomorphic adenoma gene 1 3.1 |
| (NM_002655) |
|  |
| PLD4 Phospholipase D family, member 4 8.5 |
| (NM_138790) |
|  |
| PLDN Pallidin homolog (mouse) 5.1 |
| (NM_012388) |
|  |
| PLEKHH2 Pleckstrin homology domain containing, 10.3 |
| (NM_172069) family H (with MyTH4 |
| domain)member 2 |
|  |
| PLEKHM3 Pleckstrin homology domain containing, 12.4 |
| (NM_001080475) family M, member 3 |
|  |
| PMCH Pro-melanin-concentrating hormone 11.8 |
| (NM_002674) |
|  |
| PMS2 PMS2 postmeiotic segregation increased 2 7.2 |
| (NM_000535) (S. cerevisiae) |
|  |
| PNMAL2 PNMA-like 2 5.9 |
| (NM_020709) |
|  |
| PNPLA6 Patatin-like phospholipase domain containing 6 5.1 |
| (NM_006702) |
|  |
| POLR1B Polymerase (RNA) I polypeptide B, 128kDa 8.4 |
| (BX647683) |
|  |
| POM121L9P POM121 membrane glycoprotein- 7.6 |
| (NR_003714) like 9 (rat) pseudogene |
|  |
| PPARA Peroxisome proliferator-activated 4.4 |
| (NM_005036) receptor alpha |
|  |
| PPAT Phosphoribosyl pyrophosphate 5.8 |
| (NM_002703) amidotransferase |
|  |
| PPFIA1 Protein tyrosine phosphatase, receptor type, f 6.4 |
| (NM_177423) polypeptide (PTPRF), interacting protein |
| (liprin), alpha 1 |
|  |
| PPIA Peptidylprolyl isomerase A (cyclophilin A) -4.4 |
| (NM_021130) |
|  |
| PPIC Peptidylprolyl isomerase C (cyclophilin C) -4.0 |
| (NM_000943) |
|  |
| PPP1R3C Protein phosphatase 1, regulatory (inhibitor) 7.1 |
| (NM_005398) subunit 3C |
|  |
| PPP1R9B Protein phosphatase 1, regulatory (inhibitor) 22.9 |
| (NM_032595) subunit 9B |
|  |
| PRAGMIN Homolog of rat pragma of Rnd2 -3.1 |
| (NM_001080826) |
|  |
| PRKAR1B Protein kinase, cAMP-dependent, regulatory, 4.5 |
| (NM_002735) type I, beta |
|  |
| PRKAR2B Protein kinase, cAMP-dependent, regulatory, 6.8 |
| (NM_002736) type II, beta |
|  |
| PRKG1 Protein kinase, cGMP-dependent, type I 14.7 |
| (NM_006258) |
|  |
| PRR5 Proline rich 5 (renal) -7.3 |
| (NM_015366) |
|  |
| PSEN2 Presenilin 2 (Alzheimer disease 4) 3.4 |
| (NM_000447) |
|  |
| PTHLH Parathyroid hormone-like hormone 8.7 |
| (M31157) |
|  |
| PTPRB Protein tyrosine phosphatase, receptor type, B 6.2 |
| (NM_001109754) |
|  |
| PVRL3 Poliovirus receptor-related 3 4.8 |
| (NM_015480) |
|  |
| RAB3B RAB3B, member RAS oncogene family 12.6 |
| (NM_002867) |
|  |
| RAB22A RAB22A, member RAS oncogene family 6.1 |
| (NM_020673) |
|  |
| RAB30 RAB30, member RAS oncogene family 5.8 |
| (NM_014488) |
|  |
| RAPGEF6 Rap guanine nucleotide exchange factor 6.1 |
| (NM_001164386) (GEF) 6 |
|  |
| RASGRF1 Ras protein-specific guanine 17.0 |
| (NM_002891) nucleotide-releasing factor 1 |
|  |
| RASL11A RAS-like, family 11, member A 6.8 |
| (NM_206827) |
|  |
| RASSF5 Ras association (RalGDS/AF-6) domain family 22.3 |
| (NM_182663) member 5 |
|  |
| RBM28 RNA binding motif protein 28 3.9 |
| (NM_018077) |
|  |
| RBM43 RNA binding motif protein 43 6.7 |
| (NM_198557) |
|  |
| RBMS3 RNA binding motif, single stranded 7.3 |
| (NM_014483) interacting protein |
|  |
| RBMX RNA binding motif protein, X-linked 6.3 |
| (NM_002139) |
|  |
| RDH5 Retinol dehydrogenase 5 (11-cis/9-cis) 8.5 |
| (NM_002905) |
|  |
| RDH16 Retinol dehydrogenase 16 (all-trans) 7.0 |
| (NM_003708) |
|  |
| RERG RAS-like, estrogen-regulated, growth inhibitor 3.6 |
| (NM_032918) |
|  |
| RET Ret proto-oncogene 10.0 |
| (NM_020975) |
|  |
| REXO4 REX4, RNA exonuclease 4 homolog 9.3 |
| (NM_020385) (S. cerevisiae) |
|  |
| RFPL3 Ret finger protein-like 3 11.4 |
| (NM_006604) |
|  |
| RFPL3S RFPL3 antisense RNA (non-protein coding) 6.5 |
| (NR_001450) |
|  |
| RFX5 Regulatory factor X, 5 (influences HLA class II 7.5 |
| (NM_000449) expression) |
|  |
| RGS5 Regulator of G-protein signaling 5 4.1 |
| (NM_003617) |
|  |
| RGS11 Regulator of G-protein signaling 11 9.3 |
| (BC040504) |
|  |
| RHOXF2B Rhox homeobox family, member 2B 8.7 |
| (NM_001099685) |
|  |
| RIC8B Resistance to inhibitors of cholinesterase 8 8.3 |
| (NM_018157) homolog B (C. elegans) |
|  |
| RIF1 RAP1 interacting factor homolog (yeast) 5.1 |
| (NM_018151) |
|  |
| RIMKLA Ribosomal modification protein rimK-like 15.0 |
| (NM_173642) family member A |
|  |
| RIMS3 Regulating synaptic membrane exocytosis 3 4.4 |
| (NM_014747) |
|  |
| RMND1 Required for meiotic nuclear division 1 4.4 |
| (NM_017909) homolog (S. cerevisiae) |
|  |
| RNASEH1 Ribonuclease H1 8.4 |
| (NM_002936) |
|  |
| RNF182 Ring finger protein 182 8.7 |
| (NM_152737) |
|  |
| RNF213 Ring finger protein 213 8.6 |
| (NM_020914) |
|  |
| RNF214 Ring finger protein 214 6.2 |
| (NM_001077239) |
|  |
| RNF216 Ring finger protein 216 4.5 |
| (NM_207111) |
|  |
| ROBO2 Roundabout, axon guidance 10.6 |
| (NM_002942) receptor, homolog 2 (Drosophila) |
|  |
| RORA RAR-related orphan receptor A 4.1 |
| (NM_134260) |
|  |
| RP11-94I2.2 Neuroblastoma breakpoint family, 4.8 |
| (NM_183372) member 11-like |
|  |
| RP1-21O18.1 Kazrin 9.4 |
| (NM_015209) |
|  |
| RPL32P3 Ribosomal protein L32 pseudogene 3 14.6 |
| (NR_003111) |
|  |
| RRP1B Ribosomal RNA processing1 homolog B 6.1 |
| (NM_015056) (S. cerevisiae) |
|  |
| RSBN1 Round spermatid basic protein 1 5.2 |
| (NM_018364) |
|  |
| RTKN Rhotekin 4.9 |
| (NM_033046) |
|  |
| RYBP RING1 and YY1 binding protein 5.8 |
| (NM_012234) |
|  |
| S1PR5 Sphingosine-1-phosphate receptor 5 15.5 |
| (NM_030760) |
|  |
| SAE1 SUMO1 activating enzyme subunit 1 10.4 |
| (NM_005500) |
|  |
| SAMD9 Sterile alpha motif domain containing 9 7.6 |
| (NM_017654) |
|  |
| SAMD12 Sterile alpha motif domain containing 12 10.5 |
| (NM_001101676) |
|  |
| SCAMP1 Secretory carrier membrane protein 1 10.5 |
| (NM_004866) |
|  |
| SCAND2 SCAN domain containing 2 pseudogene 14.2 |
| (NR_004859) |
|  |
| SCML1 Sex comb on midleg-like1 (Drosophila) 9.2 |
| (NM_001037540) |
|  |
| SCOC Short coiled-coil protein 4.5 |
| (NM_032547) |
|  |
| SDR9C7 Short chain dehydrogenase/reductase 14.4 |
| (AK122782) family 9C, member 7 |
|  |
| SEC22C SEC22 vesicle trafficking protein homolog C 12.3 |
| (NM_032970) (S. cerevisiae) |
|  |
| SEC24B SEC24 family, member B(S. cerevisiae) 3.3 |
| (NM_006323) |
|  |
| SEC61A2 Sec61 alpha 2 subunit (S. cerevisiae) 7.7 |
| (NM_018144) |
|  |
| SECISBP2L SECIS binding protein 2-like 8.6 |
| (NM_014701) |
|  |
| SEMA3G Sema domain, immunoglobulin domain (Ig), 4.6 |
| (NM_020163) short basic domain, secreted, (semaphorin) 3G |
|  |
| SERINC3 Serine incorporator 3 15.3 |
| (NM_198941) |
|  |
| SF3A1 Splicing factor 3a, subunit 1, 120kDa -3.7 |
| (NM_005877) |
|  |
| SFRS1 Splicing factor, arginine/ serine-rich 1 8.7 |
| (NM_001078166) |
|  |
| SFRS4 Splicing factor, arginine/serine-rich 4 8.3 |
| (NM_005626) |
|  |
| SFRS18 Splicing factor, arginine/serine- rich 18 3.3 |
| (NM_032870) |
|  |
| SFTA1P Surfactant associated 1 14.8 |
| (NR_027082) |
|  |
| SHE Src homology 2 domain containing E 8.2 |
| (NM_001010846) |
|  |
| SHMT1 Serine hydroxymethyltransferase1 (soluble) 8.3 |
| (Y14488) |
|  |
| SHOX Short stature homeobox 27.4 |
| (U89331) |
|  |
| SIGLECP3 Sialic acid binding Ig-like lectin, pseudogene 3 4.1 |
| (NR_002804) |
|  |
| SIPA1L3 Signal-induced proliferation- associated 1 like 3 11.2 |
| (NM_015073) |
|  |
| SLAIN2 SLAIN motif family, member 2 7.4 |
| (AK025264) |
|  |
| SLC13A4 Solute carrier family 13 (sodium/ sulfate 6.5 |
| (NM_012450) symporters), member 4 |
|  |
| SLC16A13 Solute carrier family 16, member 13 19.0 |
| (NM_201566) (monocarboxylic acid transporter 13) |
|  |
| SLC16A14 Solute carrier family 16, member 14 6.1 |
| (NM_152527) (monocarboxylic acid transporter 14) |
|  |
| SLC22A17 Solute carrier family 22, member 17 5.5 |
| (NM_016609) |
|  |
| SLC25A42 Solute carrier family 25, member 42 5.5 |
| (NM_178526) |
|  |
| SLC30A10 Solute carrier family 30, member 10 12.0 |
| (NM_018713) |
|  |
| SLCO1B1 Solute carrier organic anion transporter 12.5 |
| (NM_006446) family, member 1B1 |
|  |
| SLCO1B3 Solute carrier organic anion transporter 11.4 |
| (NM_019844) family, member 1B3 |
|  |
| SMAGP Small cell adhesion glycoprotein 6.1 |
| (NM_001031628) |
|  |
| SMCHD1 Structural maintenance of chromosomes flexible 4.7 |
| (NM_015295) hinge domain containing 1 |
|  |
| SMPD3 Sphingomyelin phosphodiesterase 3, neutral 9.0 |
| (NM_018667) membrane (neutral sphingomyelinase II) |
|  |
| SNCA Synuclein, alpha (non A4 component of 4.5 |
| (NM_000345) amyloid precursor) |
|  |
| SOCS5 Suppressor of cytokine signaling 5 9.9 |
| (NM_144949) |
|  |
| SOCS6 Suppressor of cytokine signaling 6 8.0 |
| (NM_004232) |
|  |
| SPDYE2 Speedy homolog E2 (Xenopus laevis) 9.2 |
| (NM_001031618) |
|  |
| SPDYE3 Speedy homolog E3 (Xenopus laevis) 5.5 |
| NM_001004351) |
|  |
| SPG21 Spastic paraplegia 21 (autosomal recessive, -3.8 |
| (NM_016630) Mast syndrome) |
|  |
|  |
| SPINK6 Serine peptidase inhibitor, Kazal type 6 14.1 |
| (NM_205841) |
|  |
| SPINT2 Serine peptidase inhibitor, Kunitz type, 2 -6.5 |
| (NM_021102) |
|  |
| SPRR1A Small proline-rich protein 1A 9.2 |
| (NM_005987) |
|  |
| SPTB Spectrin, beta, erythrocytic 15.4 |
| (NM_001024858) |
|  |
| SRP19 Signal recognition particle 19kDa 4.9 |
| (NM_003135) |
|  |
| SRRD SRR1 domain containing 6.4 |
| (NM_001013694) |
|  |
| SSBP2 Single-stranded DNA binding protein 2 5.1 |
| (NM_012446) |
|  |
| SSBP3 Single stranded DNA binding protein 3 6.3 |
| (NM_001009955) |
|  |
| SSTR1 Somatostatin receptor 1 27.4 |
| (NM_001049) |
|  |
| STAC SH3 and cysteine rich domain 22.8 |
| (NM_003149) |
|  |
| STAT2 Signal transducer and activator of transcription 2, 5.6 |
| (NM_005419) 113kDa |
|  |
| STBD1 Starch binding domain 1 16.0 |
| (AK074635) |
|  |
| STIP1 Stress-induced-phosphoprotein 1 14.0 |
| NM_006819) |
|  |
| STK17A Serine/threonine kinase 17a 8.3 |
| (NM_004760) |
|  |
| STK17B Serine/threonine kinase 17b 8.6 |
| (NM_004226) |
|  |
| STXBP3 Syntaxin binding protein 3 5.0 |
| (NM_007269) |
|  |
| STXBP4 Syntaxin binding protein 4 6.0 |
| (NM_178509) |
|  |
| SUPT7L Suppressor of Ty 7(S. cerevisiae)-like 7.6 |
| (NM_014860) |
|  |
| SURF4 Surfeit 4 5.9 |
| (NM_033161) |
|  |
| SVIP Small VCP/p97-interacting protein 4.5 |
| (NM_148893) |
|  |
| SYCP2 Synaptonemal complex protein 2 11.1 |
| (NM_014258) |
|  |
| SYDE2 Synapse defective 1, Rho GTPase, 6.8 |
| (NM_032184) homolog 2 (C. elegans) |
|  |
| SYTL2 Synaptotagmin-like 2 13.2 |
| (NM_206927) |
|  |
| TARDBP TAR DNA binding protein 3.4 |
| (NM_007375) |
|  |
| TAS1R1 Taste receptor, type 1, member 1 9.3 |
| (NM_138697) |
|  |
| TBC1D25 TBC1 domain family, member 25 15.6 |
| (NM_002536) |
|  |
| TBC1D3B TBC1 domain family, member 3B 4.0 |
| (NM_001001417) |
|  |
| TBCEL Tubulin folding cofactor E-like 6.3 |
| (NM_152715) |
|  |
| TCF15 Transcription factor 15(basic helix-loop-helix) 27.6 |
| (NM_004609) |
|  |
| TDGF1 Teratocarcinoma-derived growth factor 1 8.5 |
| (NM_003212) |
|  |
| TDRD3 Tudor domain containing 3 3.2 |
| (NM_030794) |
|  |
| TET1 Tet oncogene 1 5.6 |
| (NM_030625) |
|  |
| TET2 Tet oncogene family member 2 8.6 |
| (NM_017628) |
|  |
| THAP6 THAP domain containing 6 12.1 |
| (NM_144721) |
|  |
| TIGD6 Tigger transposable element derived 6 11.3 |
| (NM_030953) |
|  |
| TIMM8A Translocase of inner mitochondrial 4.5 |
| (NM_004085) membrane 8 homolog A (yeast) |
|  |
| TM7SF2 Transmembrane 7 superfamily member 2 7.8 |
| (NM_003273) |
|  |
| TMEM159 Transmembrane protein 159 11.9 |
| (NM_020422) |
|  |
| TMEM169 Transmembrane protein 169 15.7 |
| (NM_138390) |
|  |
| TMEM180 Transmembrane protein 180 5.2 |
| (NM_024789) |
|  |
| TMEM229A Transmembrane protein 229A 8.5 |
| (NM_001136002) |
|  |
| TMLHE Trimethyllysine hydroxylase, epsilon 7.9 |
| (NM_018196) |
|  |
| TNRC4 Trinucleotide repeat containing 4 14.2 |
| (NM_007185) |
|  |
| TNS1 Tensin 1 8.3 |
| (NM_022648) |
|  |
| TOLLIP Toll interacting protein 5.0 |
| (NM_019009) |
|  |
| TP53INP2 Tumor protein p53 inducible nuclear protein 2 8.2 |
| (NM_021202) |
|  |
| TPCN1 Two pore segment channel 1 4.8 |
| (NM_001143819) |
|  |
| TRANK1 Tetratricopeptide repeat and 5.8 |
| (NM_014831) ankyrin repeat containing 1 |
|  |
| TRIB2 Tribbles homolog 2 (Drosophila) 5.8 |
| (NM_021643) |
|  |
| TRIM7 Tripartite motif-containing 7 8.5 |
| (NM_033342) |
|  |
| TRIM27 Tripartite motif-containing 27 4.3 |
| (NM_006510) |
|  |
| TRIM41 Tripartite motif-containing 41 11.4 |
| (NM_033549) |
|  |
| TRIP12 Thyroid hormone receptor interactor 12 13.0 |
| (NM_004238) |
|  |
| TRPC7 Transient receptor potential cation channel, 23.9 |
| (NM_020389) subfamily C, member 7 |
|  |
| TSPAN14 Tetraspanin 14 15.5 |
| (NM_030927) |
|  |
| TSPAN32 Tetraspanin 32 4.5 |
| (NM_139022) |
|  |
| TSPYL1 TSPY-like 1 6.8 |
| (NM_003309) |
|  |
| TSPYL6 TSPY-like 6 10.4 |
| (NM_001003937) |
|  |
| TTC28 Tetratricopeptide repeat domain 28 14.2 |
| (NM_001145418) |
|  |
| TTTY4C Testis-specific transcript, Y-linked 4C 16.4 |
| (NR_002177) (non-protein coding) |
|  |
| TUBB4 Tubulin, beta 4 6.4 |
| (NM_006087) |
|  |
| TUBE1 Tubulin, epsilon 1 5.8 |
| (NM_016262) |
|  |
| TXLNB Taxilin beta 13.7 |
| (NM_153235) |
|  |
| TXNL1 Thioredoxin-like 1 14.0 |
| (BC034791) |
|  |
| UEVLD UEV and lactate/malate dehyrogenase domains 8.0 |
| (NM_001040697) |
|  |
| UGT2B4 UDP glucuronosyltransferase2 family, 26.9 |
| (NM_021139) polypeptide B4 |
|  |
| UPB1 Ureidopropionase, beta 15.4 |
| (NM_016327) |
|  |
| UPF2 UPF2 regulator of nonsense transcripts 10.3 |
| (NM_080599) homolog (yeast) |
|  |
| UPF3A UPF3 regulator of nonsense transcripts 12.2 |
| (NM_023011) homolog A (yeast) |
|  |
| USP24 Ubiquitin specific peptidase 24 4.1 |
| (NM_015306) |
|  |
| USP25 Ubiquitin specific peptidase 25 7.6 |
| (NM_013396) |
|  |
| USP46 Ubiquitin specific peptidase 46 4.0 |
| (NM_022832) |
|  |
| VN1R1 Vomeronasal 1 receptor 1 12.1 |
| (NM_020633) |
|  |
| WBSCR16 Williams-Beuren syndrome chromosome 14.3 |
| (NM_030798) region 16 |
|  |
| WBSCR17 Williams-Beuren syndrome chromosome 3.8 |
| (NM_022479) region 17 |
|  |
| WDR5B WD repeat domain 5B 4.4 |
| (NM_019069) |
|  |
| WDR13 WD repeat domain 13 20.3 |
| (L08237) |
|  |
| WDR20 WD repeat domain 20 8.8 |
| (BC030654) |
|  |
| WDR36 WD repeat domain 36 4.1 |
| (NM_139281) |
|  |
| WNT8B Wingless-type MMTV integration site 14.4 |
| (NM_003393) family, member 8B |
|  |
| XK X-linked Kx blood group(McLeod syndrome) 4.1 |
| (NM_021083) |
|  |
| XPO4 Exportin 4 4.3 |
| (NM_022459) |
|  |
| XPO5 Exportin 5 4.8 |
| (NM_020750) |
|  |
| XRRA1 X-ray radiation resistance associated 1 5.8 |
| (NM_182969) |
| YES1 V-yes-1 Yamaguchi sarcoma viral oncogene 5.4 |
| (NM_005433) homolog 1 |
|  |
| YWHAE Tyrosine 3-monooxygenase/ tryptophan 5- 3.4 |
| (NM_006761) monooxygenase activation protein, |
| epsilon polypeptide |
|  |
| ZBTB7B Zinc finger and BTB domain containing 7B 6.5 |
| (NM_015872) |
|  |
| ZBTB25 Zinc finger and BTB domain containing 25 4.5 |
| (NM_006977) |
|  |
| ZC3H12C Zinc finger CCCH-type containing 12C 3.7 |
| (NM_033390) |
|  |
| ZCCHC4 Zinc finger, CCHC domain containing 4 4.5 |
| (NM_024936) |
|  |
| ZCRB1 Zinc finger CCHC-type and RNA binding motif 1 4.4 |
| (NM_033114) |
|  |
| ZDHHC15 Zinc finger, DHHC-type containing 15 7.9 |
| (NM_001146257) |
|  |
| ZFAND3 Zinc finger, AN1-type domain 3 9.7 |
| (NM_021943) |
|  |
| ZFP2 Zinc finger protein 2 homolog (mouse) 5.4 |
| (NM_030613) |
|  |
| ZFP14 Zinc finger protein 14 homolog (mouse) 5.2 |
| (NM_020917) |
|  |
| ZFY Zinc finger protein, Y-linked 6.3 |
| (NM_003411) |
|  |
| ZIK1 Zinc finger protein interacting with K protein 1 5.3 |
| (NM_001010879) homolog (mouse) |
|  |
| ZKSCAN3 Zinc finger with KRAB and SCAN domains 3 6.3 |
| (NM_024493) |
|  |
| ZNF2 Zinc finger protein 2 11.9 |
| (NM_021088) |
|  |
| ZNF12 Zinc finger protein 12 3.9 |
| (NM_016265) |
|  |
| ZNF17 Zinc finger protein 17 3.5 |
| (NM_006959) |
|  |
| ZNF37A Zinc finger protein 37A 5.2 |
| (NM_001007094) |
|  |
| ZNF66 Zinc finger protein 66 3.6 |
| (XM_001714861) |
|  |
| ZNF135 Zinc finger protein 135 4.4 |
| (AL157426) |
|  |
| ZNF154 Zinc finger protein 154 9.3 |
| (BC152561) |
|  |
| ZNF177 Zinc finger protein 177 7.1 |
| (NM_003451) |
|  |
| ZNF214 Zinc finger protein 214 8.4 |
| (NM_013249) |
|  |
| ZNF227 Zinc finger protein 227 6.3 |
| (NM_182490) |
|  |
| ZNF235 Zinc finger protein 235 9.0 |
| (NM_004234) |
|  |
| ZNF248 Zinc finger protein 248 4.1 |
| (NM_021045) |
|  |
| ZNF254 Zinc finger protein 254 10.1 |
| (NM_203282) |
|  |
| ZNF257 Zinc finger protein 257 17.7 |
| (NM_033468) |
|  |
| ZNF274 Zinc finger protein 274 15.2 |
| (NM_133502) |
|  |
| ZNF283 Zinc finger protein 283 6.2 |
| (NM_181845) |
|  |
| ZNF323 Zinc finger protein 323 4.0 |
| (NM_030899) |
|  |
| ZNF343 Zinc finger protein 343 9.0 |
| (NM_024325) |
|  |
| ZNF346 Zinc finger protein 346 4.7 |
| (NM_012279) |
|  |
| ZNF347 Zinc finger protein 347 5.6 |
| (NM_032584) |
|  |
| ZNF366 Zinc finger protein 366 5.0 |
| (AK090694) |
|  |
| ZNF385B Zinc finger protein 385B 9.1 |
| (NM_152520) |
|  |
| ZNF420 Zinc finger protein 420 13.2 |
| (NM_144689) |
|  |
| ZNF430 Zinc finger protein 430 5.3 |
| (NM_025189) |
|  |
| ZNF501 Zinc finger protein 501 5.0 |
| (NM_145044) |
|  |
| ZNF507 Zinc finger protein 507 5.7 |
| (NM_014910) |
|  |
| ZNF551 Zinc finger protein 551 4.4 |
| (NM_138347) |
|  |
| ZNF570 Zinc finger protein 570 9.3 |
| (NM_144694) |
|  |
| ZNF571 Zinc finger protein 571 7.5 |
| (NM_016536) |
|  |
| ZNF577 Zinc finger protein 577 3.6 |
| (NM_032679) |
|  |
| ZNF578 Zinc finger protein 578 5.9 |
| (NM_001099694) |
|  |
| ZNF596 Zinc finger protein 596 6.5 |
| (NM_001042416) |
|  |
| ZNF610 Zinc finger protein 610 11.0 |
| (NM_173530) |
|  |
| ZNF626 Zinc finger protein 626 5.8 |
| (NM_001076675) |
|  |
| ZNF644 Zinc finger protein 644 4.4 |
| (NM_201269) |
|  |
| ZNF667 Zinc finger protein 667 4.4 |
| (NM_022103) |
|  |
| ZNF680 Zinc finger protein 680 9.0 |
| (NM_178558) |
|  |
| ZNF726 Zinc finger protein 726 9.9 |
| (XM_001726947) |
|  |
| ZNF765 Zinc finger protein 765 8.7 |
| (BC017357) |
|  |
| ZNF770 Zinc finger protein 770 20.2 |
| (NM_014106) |
|  |
| ZNF826 Zinc finger protein 826 4.3 |
| (NM_001039884) |
|  |
| ZNF841 Zinc finger protein 841 6.0 |
| (NM_001136499) |
|  |
| ZNF853 Zinc finger protein 853 15.5 |
| (NM_017560) |
|  |
| ZNF879 Zinc finger protein 879 10.5 |
| (NM_001136116) |
|  |
| ZNFX1 Zinc finger, NFX1-type containing 1 7.4 |
| (NM_021035) |
|  |
| ZRANB2 Zinc finger, RAN-binding domain containing 2 6.0 |
| (NM_203350) |
|  |
| ZSCAN2 Zinc finger and SCAN domain containing 2 7.0 |
| (NM_181877) |
| __________________________________________________________________________ |
|  |
| **Proliferative-to-secretory phase** |
|  |
| Eutopic |
|  |
| ADAM8 ADAM metallopeptidase domain 8 -3.6 |
| (NM_001109) |
|  |
| ADAMTSL5 ADAMTS-like 5 -9.6 |
| (NM_213604) |
|  |
| ANKS1A Ankyrin repeat and sterile alpha motif 3.2 |
| (NM_015245) domain containing 1A |
|  |
| ARHGAP17 Rho GTPase activating protein 17 3.1 |
| (NM_001006634) |
|  |
| BAT2L HLA-B associated transcript 2-like -6.1 |
| (NM_013318) |
|  |
| BEND7 BEN domain containing 7 -4.1 |
| (NM_001100912) |
|  |
| C13orf37 Chromosome 13 open reading frame 37 -4.4 |
| (NM_001071775) |
|  |
| CCDC84 Coiled-coil domain containing 84 3.3 |
| (NM_198489) |
|  |
| CCDC130 Coiled-coil domain containing 130 -6.2 |
| (NM_030818) |
|  |
| CCHCR1 Coiled-coil alpha-helical rod protein 1 4.8 |
| (NM_019052) |
|  |
| CES2 Carboxylesterase 2 (intestine, liver) -5.5 |
| (NM_003869) |
|  |
| CLEC14A C-type lectin domain family 14, member A -6.4 |
| (NM_175060) |
|  |
| CPM Carboxypeptidase M 4.8 |
| (NM_001874) |
|  |
| CSNK2A2 Casein kinase 2, alpha prime polypeptide -6.5 |
| (AK125922) |
|  |
| CSRNP1 Cysteine-serine-rich nuclear protein 1 -4.5 |
| (NM_033027) |
|  |
| CTR9 Ctr9, Paf1/RNA polymerase II complex -3.5 |
| (NM_014633) component, homolog (S. cerevisiae) |
|  |
| CYB561D1 Cytochrome b-561 domain containing 1 -6.4 |
| (NM_182580) |
|  |
| DCAF4 DDB1 and CUL4 associated factor 4 3.5 |
| (NM_181340) |
|  |
| ENOX1 Ecto-NOX disulfide-thiol exchanger 1 -6.7 |
| (NM_017993) |
|  |
| EZH1 Enhancer of zeste homolog 1 (Drosophila) -5.3 |
| (NM_001991) |
|  |
| FAM101B Family with sequence similarity 101, member B -4.3 |
| (NM_182705) |
|  |
| FAM198B Family with sequence similarity 198, member B -3.6 |
| (NM_016613) |
|  |
| FBXL12 F-box and leucine-rich repeat protein 12 -6.1 |
| (NM_017703) |
|  |
| FNTA Farnesyltransferase, CAAX box, alpha -4.4 |
| (NM_002027) |
|  |
| FRAT2 Frequently rearranged in advanced T-cell -6.9 |
| (NM_012083) lymphomas 2 |
|  |
| GLTPD1 Glycolipid transfer protein domain containing 1 -3.3 |
| (NM_001029885) |
|  |
| HIST1H1C Histone cluster 1, H1c -3.3 |
| (NM_005319) |
|  |
| IER5 Immediate early response 5 -6.8 |
| (NM_016545) |
|  |
| KDM4C Lysine (K)-specific demethylase 4C -5.8 |
| (NM_015061) |
|  |
| KITLG KIT ligand -3.1 |
| (NM_000899) |
|  |
| LAT2 Linker for activation of T cells family, member 2 -5.3 |
| (NM_032464) |
|  |
| LCE1F Late cornified envelope 1F -6.5 |
| (NM_178354) |
|  |
| LGTN Ligatin -7.8 |
| (NM_006893) |
|  |
| LMNB2 Lamin B2 3.5 |
| (NM_032737) |
|  |
| LOC90246 Hypothetical protein LOC90246 3.1 |
| (NR_026954) |
|  |
| LST1 Leukocyte specific transcript 1 3.6 |
| (NM_007161) |
|  |
| MAG Myelin associated glycoprotein -4.7 |
| (NM_080600) |
|  |
| MAK10 MAK10 homolog, amino-acid N-acetyltransferase -3.2 |
| (NM_024635) subunit (S. cerevisiae) |
|  |
| MRPS6 Mitochondrial ribosomal protein S6 3.5 |
| (NM_032476) |
|  |
| NID2 Nidogen 2 (osteonidogen) -5.1 |
| (NM_007361) |
|  |
| NIPAL2 NIPA-like domain containing 2 -4.0 |
| (AK025015) |
|  |
| OR2H1 Olfactory receptor, family 2, subfamily H, -3.4 |
| (NM_030883) member 1 |
|  |
| OSGIN2 Oxidative stress induced growth inhibitor -5.2 |
| (NM_004337) family member 2 |
|  |
| PCGF5 Polycomb group ring finger 5 3.1 |
| (NM_032373) |
|  |
| PDLIM5 PDZ and LIM domain 5 -3.7 |
| (NM_006457) |
|  |
| POFUT2 Protein O-fucosyltransferase 2 -3.7 |
| (NM_133635) |
|  |
| PP14571 Similar to hCG1777210 -8.2 |
| (NR_024014) |
|  |
| PPP1CC Protein phosphatase 1, catalytic subunit, -3.5 |
| (NM_002710) gamma isoform |
|  |
| PPP1R3B Protein phosphatase 1, regulatory (inhibitor) -4.1 |
| (NM_024607) subunit 3B |
|  |
| PPP2R3A Protein phosphatase 2 (formerly 2A), 4.0 |
| (NM_002718) regulatory subunit B'', alpha |
|  |
| PRAF2 PRA1 domain family, member 2 3.4 |
| (NM_007213) |
|  |
| PRPF40B PRP40 pre-mRNA processing factor 40 -4.8 |
| (NM_001031698) homolog B (S. cerevisiae) |
|  |
| PTOV1 Prostate tumor overexpressed 1 3.3 |
| (NM_017432) |
|  |
| PTPRA Protein tyrosine phosphatase, receptor type, A -6.5 |
| (NM_002836) |
|  |
| PTPRF Protein tyrosine phosphatase, receptor type, F -8.4 |
| (NM_002840) |
|  |
| RBM18 RNA binding motif protein 18 -3.4 |
| (NM_033117) |
|  |
| RPS3A Ribosomal protein S3A -5.7 |
| (NM_001006) |
|  |
| SH3BGRL SH3 domain binding glutamic acid-rich -4.2 |
| (NM_003022) protein like |
|  |
| SLC2A11 Solute carrier family 2 (facilitated glucose 3.4 |
| (NM_030807) transporter), member 11 |
|  |
| SLC30A2 Solute carrier family 30 (zinc transporter), -4.5 |
| (NM_001004434) member 2 |
|  |
| SLC31A2 Solute carrier family 31 (copper transporters), -4.5 |
| (NM_001860) member 2 |
|  |
| SPATA7 Spermatogenesis associated 7 -3.1 |
| (NM_018418) |
|  |
| SPRR1A Small proline-rich protein 1A 3.6 |
| (NM_005987) |
|  |
| SQLE Squalene epoxidase -5.0 |
| (NM_003129) |
|  |
| STK24 Serine/threonine kinase 24 (STE20 homolog, -3.3 |
| (NM_001032296) yeast) |
|  |
| STK25 Serine/threonine kinase 25 (STE20 homolog, 3.3 |
| (NM_006374) yeast) |
|  |
| STRA6 Stimulated by retinoic acid gene 6 homolog 3.1 |
| (NM_022369) (mouse) |
|  |
| SUSD3 Sushi domain containing 3 -3.6 |
| (NM_145006) |
|  |
| TBCEL Tubulin folding cofactor E-like -5.2 |
| (NM_152715) |
|  |
| TMEM66 Transmembrane protein 66 -4.2 |
| (NM_016127) |
|  |
| TMEM87B Transmembrane protein 87B -3.3 |
| (NM_032824) |
|  |
| TMEM158 Transmembrane protein 158 -4.5 |
| (NM_015444) |
|  |
| TRIM27 Tripartite motif-containing 27 -3.6 |
| (NM_006510) |
|  |
| TROAP Trophinin associated protein (tastin) 7.0 |
| (NM_005480) |
|  |
| UCN2 Urocortin 2 -5.2 |
| (NM_033199) |
|  |
| UGDH UDP-glucose dehydrogenase 4.9 |
| (NM_003359) |
|  |
| VWC2 Von Willebrand factor C domain containing 2 3.9 |
| (NM_198570) |
|  |
| WFDC10B WAP four-disulfide core domain 10B -3.1 |
| (NM_172006) |
|  |
| ZNF726 Zinc finger protein 726 -6.5 |
| (XM_001726947) |
|  |
|  |
| Ectopic |
| ABCG2 ATP-binding cassette, sub-family G -3.9 |
| (NM_004827) (WHITE), member 2 |
|  |
| ABHD4 Abhydrolase domain containing 4 -3.8 |
| (NM_022060) |
|  |
| ADAM8 ADAM metallopeptidase domain 8 -3.1 |
| (NM_001109) |
|  |
| AHDC1 AT hook, DNA binding motif, containing 1 -3.4 |
| (NM_001029882) |
|  |
| ANKH Ankylosis, progressive homolog (mouse) -3.1 |
| (NM_054027) |
|  |
| ANKRD58 Ankyrin repeat domain 58 -3.1 |
| (NM_001105576) |
|  |
| ARL17P1 ADP-ribosylation factor-like 17 pseudogene 1 -3.9 |
| (NM_016632) |
|  |
| ATIC 5-aminoimidazole-4-carboxamide ribonucleotide -3.1 |
| (NM_004044) formyltransferase/IMP cyclohydrolase |
|  |
| BHLHE40 Basic helix-loop-helix family, member e40 -5.0 |
| (NM_003670) |
|  |
| C20orf141 Chromosome 20 open reading frame 141 -4.7 |
| (NM_080739) |
|  |
| CD47 CD47 molecule -3.4 |
| (NM_001777) |
|  |
| CDR2 Cerebellar degeneration-related protein 2, -3.1 |
| (NM_001802) 62kDa |
|  |
| CHRDL2 Chordin-like 2 -7.4 |
| (NM_015424) |
|  |
| CHST8 Carbohydrate (N-acetylgalactosamine 4-0) -3.4 |
| (NM_022467) sulfotransferase 8 |
|  |
| CIRBP Cold inducible RNA binding protein -3.1 |
| (NR_023312) |
|  |
| CLDN11 Claudin 11 -4.4 |
| (NM_005602) |
|  |
| COPZ1 Coatomer protein complex, subunit zeta 1 -3.4 |
| (NM_016057) |
|  |
| CTH Cystathionase (cystathionine gamma-lyase) -4.1 |
| (NM_001902) |
|  |
| D2HGDH D-2-hydroxyglutarate dehydrogenase -4.8 |
| (NM_152783) |
|  |
| D4S234E DNA segment on chromosome 4 (unique) -4.5 |
| (NM_014392) 234 expressed sequence |
|  |
| DCAF4 DDB1 and CUL4 associated factor 4 -3.3 |
| (NM_181340) |
|  |
| DENND1A DENN/MADD domain containing 1A -3.1 |
| (NM_020946) |
|  |
| DENND1C DENN/MADD domain containing 1C -3.3 |
| (NM_024898) |
|  |
| DIRAS3 DIRAS family, GTP-binding RAS-like 3 -4.9 |
| (NM_004675) |
|  |
| DLG4 Discs, large homolog 4 (Drosophila) -4.1 |
| (NM_001365) |
|  |
| DLK1 Delta-like 1 homolog (Drosophila) -5.1 |
| (NM_003836) |
|  |
| DNAJB5 DnaJ (Hsp40) homolog, subfamily B, member 5 -4.8 |
| (NM_012266) |
|  |
| DOK4 Docking protein 4 -3.3 |
| (NM_018110) |
|  |
| DPT Dermatopontin -3.4 |
| (NM_001937) |
|  |
| EBP Emopamil binding protein (sterol isomerase) -3.4 |
| (NM_006579) |
|  |
| EGR3 Early growth response 3 -4.8 |
| (NM_004430) |
|  |
| ELAVL1 ELAV (embryonic lethal, abnormal vision, -3.7 |
| (NM_001419) Drosophila)-like 1 (Hu antigen R) |
|  |
| ENO2 Enolase 2 (gamma, neuronal) -3.2 |
| (NM_001975) |
|  |
| FARP1 FERM, RhoGEF (ARHGEF) and pleckstrin -3.1 |
| (NM_005766) domain protein 1 (chondrocyte-derived) |
|  |
| FGR Gardner-Rasheed feline sarcoma viral -4.5 |
| (NM_001042747) (v-fgr) oncogene homolog |
|  |
| FLCN Folliculin -3.2 |
| (NM_144606) |
|  |
| FOLR2 Folate receptor 2 (fetal) -4.7 |
| (NM_000803) |
|  |
| GADD45B Growth arrest and DNA-damage-inducible, beta -3.1 |
| (NM_015675) |
|  |
| HCRT Hypocretin (orexin) neuropeptide precursor -4.8 |
| (NM_001524) |
|  |
| HSPA6 Heat shock 70kDa protein 6 (HSP70B') -4.1 |
| (NM_002155) |
|  |
| HSPB6 Heat shock protein, alpha-crystallin-related, B6 -4.6 |
| (NM_144617) |
|  |
| KIAA0467 KIAA0467 -3.1 |
| (NM_015284) |
|  |
| KIAA0652 KIAA0652 -7.4 |
| (NM_014741) |
|  |
| LGI4 Leucine-rich repeat LGI family, member 4 -5.4 |
| (NM_139284) |
|  |
| LOC100287521 Hypothetical protein LOC100287521 -3.7 |
| (XM_002343283) |
|  |
| LOC100289949 Hypothetical protein LOC100289949 -5.6 |
| (XM_002346836) |
|  |
| LOC286254 Hypothetical protein LOC286254 -3.6 |
| (AK092751) |
|  |
| LOC375295 Hypothetical protein LOC375295 -4.2 |
| (XM_001716150) |
|  |
| MAFF V-maf musculoaponeurotic fibrosarcoma -4.2 |
| (NM_012323) oncogene homolog F (avian) |
|  |
| MAP3K3 Mitogen-activated protein kinase kinase kinase 3 -3.7 |
| (NM_203351) |
|  |
| MMRN2 Multimerin 2 -3.4 |
| (NM_024756) |
|  |
| MPP1 Membrane protein, palmitoylated 1, 55kDa -3.3 |
| (NM_002436) |
|  |
| MT1L Metallothionein 1L (gene/pseudogene) -4.8 |
| (NR_001447) |
|  |
| MYH14 Myosin, heavy chain 14 -4.3 |
| (NM_001077186) |
|  |
| NANOG Nanog homeobox -4.8 |
| (NM_024865) |
|  |
| NAV3 Neuron navigator 3 -4.0 |
| (NM_014903) |
|  |
| NPBWR1 Neuropeptides B/W receptor 1 -3.1 |
| (NM_005285) |
|  |
| NSUN5 NOL1/NOP2/Sun domain family, member 5 -4.9 |
| (NM_148956) |
|  |
| NTF3 Neurotrophin 3 -5.1 |
| (NM_002527) |
|  |
| OSBP Oxysterol binding protein -3.4 |
| (NM_002556) |
|  |
| OSCAR Osteoclast associated, immunoglobulin-like -3.2 |
| (NM_206818) receptor |
|  |
| PA2G4 Proliferation-associated 2G4, 38kDa -3.5 |
| (NM_006191) |
|  |
| PCOLCE2 Procollagen C-endopeptidase enhancer 2 -3.1 |
| (NM_013363) |
|  |
| PDCD1 Programmed cell death 1 -3.8 |
| (NM_005018) |
|  |
| PDLIM5 PDZ and LIM domain 5 -3.9 |
| (NM_006457) |
|  |
| PLSCR4 Phospholipid scramblase 4 -3.5 |
| (NM_020353) |
|  |
| PRKAR2B Protein kinase, cAMP-dependent, -5.2 |
| (NM_002736) regulatory, type II, beta |
|  |
| PROP1 PROP paired-like homeobox 1 -3.2 |
| (NM_006261) regulatory, type II, beta |
|  |
| PVRL3 Poliovirus receptor-related 3 -3.6 |
| (NM_015480) |
|  |
| RAB24 RAB24, member RAS oncogene family -3.6 |
| (NM_001031677) |
|  |
| RABGEF1 RAB guanine nucleotide exchange factor (GEF)1 -4.8 |
| (NM_014504) |
|  |
| RAPGEF3 Rap guanine nucleotide exchange factor (GEF)3 -3.1 |
| (NM_001098531) |
|  |
| RASSF4 Ras association (RalGDS/AF-6) domain -4.5 |
| (NM_032023) family member 4 |
|  |
| RGL2 Ral guanine nucleotide dissociation -4.7 |
| (NM_004761) stimulator-like 2 |
|  |
| RGN Regucalcin (senescence marker protein-30) -3.8 |
| (NM_152869) |
|  |
| RHO Rhodopsin -4.0 |
| (NM_000539) |
|  |
| ROD1 ROD1 regulator of differentiation 1 (S. pombe) -3.1 |
| (NM_005156) |
|  |
| RRP7B Ribosomal RNA processing 7 homolog B -4.2 |
| (NR_002184) (S. cerevisiae) |
|  |
| SFXN3 Sideroflexin 3 -3.8 |
| (NM_030971) |
|  |
| SIGLEC11 Sialic acid binding Ig-like lectin 11 -4.1 |
| (NM_052884) |
|  |
| SIK1 Salt-inducible kinase 1 -3.5 |
| (NM_173354) |
|  |
| SLAMF9 SLAM family member 9 -3.9 |
| (NM_033438) |
|  |
| SLC22A11 Solute carrier family 22 (organic anion/urate -4.8 |
| (NM_018484) transporter), member 11 |
|  |
| SLC45A3 Solute carrier family 45, member 3 -5.1 |
| (NM_033102) |
|  |
| SNRPB2 Small nuclear ribonucleoprotein polypeptide B -4.0 |
| (NM_003092) |
|  |
| SPOCK2 Sparc/osteonectin, cwcv and kazal-like domains -4.3 |
| (NM_014767) proteoglycan (testican) 2 |
|  |
| SPRR2E Small proline-rich protein 2E -7.5 |
| (NM_001024209) |
|  |
| SUDS3 Suppressor of defective silencing 3 -3.7 |
| (NM_022491) homolog (S. cerevisiae) |
|  |
| TSHZ1 Teashirt zinc finger homeobox 1 -3.1 |
| (NM_005786) |
|  |
| UGDH UDP-glucose dehydrogenase -3.9 |
| (NM_003359) |
|  |
| UPK2 Uroplakin 2 -4.5 |
| (NM_006760) |
|  |
| VCAM1 Vascular cell adhesion molecule 1 -3.1 |
| (NM_001078) |
|  |
| YPEL1 Yippee-like 1 (Drosophila) -3.2 |
| (NM_013313) |
|  |
| ZDHHC7 Zinc finger, DHHC-type containing 7 -3.4 |
| (NM_017740) |
|  |
| ZSWIM1 Zinc finger, SWIM-type containing 1 -3.3 |
| (NM_080603) |
| _____________________________________________________________________________ |
|  |
| ^a^>3-fold at P<0.01 |
